# Dynamics of drinking water biofilm formation associated with *Legionella* spp. colonization

**DOI:** 10.1038/s41522-024-00573-x

**Published:** 2024-10-06

**Authors:** Céline Margot, William Rhoads, Marco Gabrielli, Margot Olive, Frederik Hammes

**Affiliations:** 1 Department of Environmental Microbiology, Eawag, Swiss Federal Institute of Aquatic Science and Technology, Dübendorf, Switzerland; 2 Department of Environmental Systems Science, Institute of Biogeochemistry and Pollutant Dynamics, ETH Zürich, Zürich, Switzerland

**Keywords:** Biofilms, Microbial ecology, Water microbiology

## Abstract

Understanding how *Legionella* spp. proliferate in multispecies biofilms is essential to develop strategies to control their presence in building plumbing. Here, we analyzed biofilm formation and *Legionella* spp. colonization on new plumbing material during 8 weeks. Biofilm formation was characterized by an initial increase in intact cell concentrations up to 9.5 × 10^5^ cells/cm^2^, followed by a steady decrease. We identified *Comamonas, Caulobacter, Schlegella*, *Blastomonas* and *Methyloversatili*s as pioneer genera in the biofilm formation process. Importantly, *L. pneumophila* was the dominant *Legionella* spp. and rapidly colonized the biofilms, with culturable cell concentrations peaking at 3.1 × 10^4^ MPN/cm^2^ after 4 weeks already. Moreover, several *Legionella* species co-occurred and had distinct dynamics of biofilm colonization. *Vermamoeba vermiformis* (*V. vermiformis*) was the dominant protist identified with 18S rRNA gene amplicon sequencing. Together our results highlight that biofilm formation upon introduction of new building plumbing material is a dynamic process where pathogenic *Legionella* species can be part of the earliest colonizers.

## Introduction

*Legionella* species are opportunistic pathogens causing Legionnaires’ disease upon the inhalation of contaminated aerosols^1,2^. Over the past decades, Legionnaires’ disease incidence increased worldwide^3–5^. *Legionella* spp. are found in aqueous and humid environments, including rivers, lakes, soil, compost and engineered water systems^6^. Biofilms in building plumbing drinking water systems act as a reservoir for these bacteria^7–11^, but they are considerably more difficult to access than the water phase and are sampled and studied less frequently^12^. However, as Flemming et al. stated: “*biofilm communities have emergent properties; that is, new properties that emerge in the biofilm that are not predictable from the study of free-living bacterial cells*”^13^. Thus, it is important to understand how *Legionella* spp. colonize building plumbing systems in the context of the overall biofilm microbiome.

*Legionella pneumophila* (*L. pneumophila*) is the most studied member of the genus *Legionella* and is most frequently associated with infection^2,4^. Multispecies biofilm communities are important for *L. pneumophila* attachment, growth and survival in environmental biofilms. Adhesion of *L. pneumophila* to biofilms depends on the overall biofilm structure^14^ and attachment to biofilms can be promoted by other bacteria, such as *Acidovorax*^15^. Moreover, building plumbing systems are low-nutrient environments and *L. pneumophila* rely on other organisms for survival^16–19^. For instance, *L. pneumophila* have been shown to persist within biofilms formed by *Klebsiella*, *Flavobacterium* and *Pseudomonas*^20^ and are also able to grow on bacterial debris^21,22^ and extracellular products of cyanobacteria^23^. Protozoan hosts, such as amoebae, are critical for *L. pneumophila* survival and replication in building plumbing systems^24–26^. *L. pneumophila* can resist phagocytosis and hijack protists’ metabolism in order to scavenge degraded peptides and proteins as a nutrient source^16,27^. Several laboratory- or pilot-scale studies with pre-formed biofilms showed *L. pneumophila* colonization of biofilms or replication in biofilms only in the presence of amoebae^28–31^, suggesting that the host is essential for *L. pneumophila* colonization of biofilms. Multispecies biofilm matrices and persistence in protists provide *L. pneumophila* with increased resistance to chemical^32^, antibacterial^33^ or thermal^34,35^ disinfection treatment.

Several studies reported on drinking water biofilm community composition^9,10^ and characteristics of biofilm formation^36,37^, but only few studies reported taxonomically detailed temporal dynamics of the microbial community during the initial biofilm formation process^38–40^. Specifically, the dynamics characterizing the establishment of *L. pneumophila* and other *Legionella* species with respect to the rest of the community remain unclear. In order to develop strategies to control the pathogen in building plumbing systems, it is essential to understand how *Legionella* spp. colonize these systems, particularly new plumbing materials. Some studies artificially introduced *L. pneumophila* in biofilm formation assays and pre-formed biofilms^28,29,41–45^ or focused on controlled bacterial consortia^15,20,30^. These systems showed that *L. pneumophila* successfully established in biofilms as a “secondary colonizer”^15^, but did not fully capture the events happening in real building plumbing systems, where environmental *Legionella* strains are naturally present and the incoming microbial community is diverse^46^.

To investigate (1) the dynamics of building plumbing system colonization by *Legionella* spp. and (2) general community dynamics during biofilm formation, we analyzed the biofilm microbial community on a synthetic polymeric plumbing material exposed to non-chlorinated drinking water during the first 8 weeks of biofilm formation and development. As material, we used ethylene propylene diene monomer (EPDM) rubber, which is typically used in sealing rings and which has previously been associated with excessive biofilm growth and specifically *Legionella* spp. colonization^47–49^. Using both culture-based and DNA-based methods, we quantified the kinetics of colonization of the naturally present *Legionella* spp. over time. We also analyzed microbial community dynamics using flow cytometry (FCM), 16S and 18S rRNA amplicon sequencing, highlighting microbial succession in the biofilm. Finally, we integrated our results in a conceptual model designed to improve understanding of the multispecies biofilm formation process.

## Results

### Biofilm development is characterized by two distinct phases

**Fig. 1:**
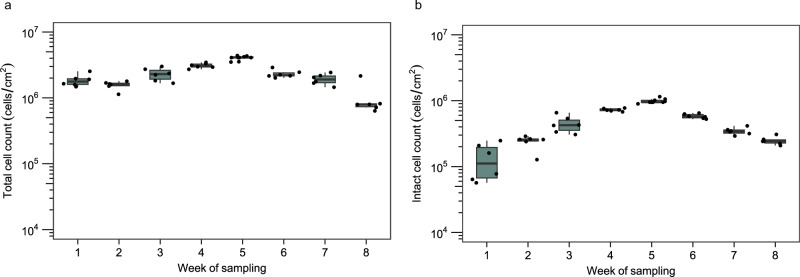
Cell counts during the first 8 weeks of biofilm development on EPDM coupons in non-chlorinated tap water at 37 °C. **a** Total cell count (TCC; *n* = 6) and **b** intact count (ICC; *n* = 6). The box shows the median and interquartile range (25th and 75th percentiles), and the whiskers show the largest value within 1.5 times the interquartile range above the 75th percentile and 1.5 times the interquartile range below the 25th percentile. Each week, TCC and ICC differed significantly from the previous week (*p* < 0.05), except weeks 1–2 and weeks 6–7 for TCC. Dots are individual data points.

Total (TCC) and intact (ICC) cell counts from the biofilms revealed two distinct development phases: an increase during the five first weeks followed by a decrease until the end of the experiment. TCC increased more than twofold from the 1st to 5th week, starting with a median concentration of 1.8 × 10^6^ cells/cm^2^ (*n* = 6) in week 1 and reaching a median concentration of 4.1 × 10^6^ cells/cm^2^ (*n* = 6) at week 5. The TCC then decreased to 8 × 10^5^ cells/cm^2^ (*n* = 6) at the end of the experiment (Fig. 1a). ICC showed a more pronounced trend, with an almost eightfold increase between week 1 and week 5 (1.2 × 10^5^ cells/cm^2^ (*n* = 6) after week 1; 9.5 × 10^5^ cells/cm^2^ (*n* = 6) after week 5). The ICC then decreased, reaching a median of 2.4 × 10^5^ cells/cm^2^ (*n* = 6) at the end of the experiment (Fig. 1b). Total organic carbon in the water phase showed an increase from 2.0 mg/L at the start of the experiment to a maximum of 3.4 mg/L on week 3, followed by a decrease to 1.4 mg/L at week 7 (Supplementary Table 1), which could indicate that less assimilable carbon was present after week 4. In the water phase, both TCC and ICC increased over time. Within the 1st week, a ca. tenfold increase in TCC and ICC was observed, reaching 1.1 × 10^6^ and 7.8 × 10^5^ cells/mL, respectively (Supplementary Fig. 1). After that, cell concentrations continued to increase, reaching a TCC and ICC of 2.6 × 10^6^ and 1.9 × 10^6^ cells/mL, respectively, at the end of the experiment.

### *Legionella* rapidly establishes in plumbing biofilms

**Fig. 2:**
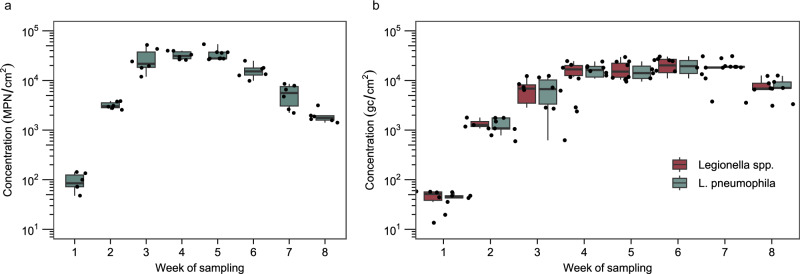
Legionella counts during the first 8 weeks of biofilm development on EPDM coupons in non-chlorinated tap water at 37 °C. **a** Culturable *L. pneumophila* measured with Legiolert (*n* = 6). **b** *Legionella* spp. and *L. pneumophila* gene copies (gc) measured with ddPCR (*n* = 6; week 8: *n* = 5). The box shows the median and interquartile range (25th and 75th percentiles), and the whiskers show the largest value within 1.5 times the interquartile range above the 75th percentile and 1.5 times the interquartile range below the 25th percentile. Each week, Legiolert values differed significantly from the previous week (*p* < 0.05), except weeks 4 and 5. For the ddPCR values, only weeks 1 and 2 varied significantly (*p* < 0.05) for both *Legionella* spp. and *L. pneumophila* gene copy numbers from 1 week to the next. However, we observed a significant increase from weeks 2 to 4 and a significant decrease from weeks 6 to 8 for both *L. pneumophila* and *Legionella* spp. Dots are individual data points.

DNA- and culture-based methods showed that indigenous *L. pneumophila* rapidly colonized the biofilms on the EPDM coupons (Fig. 2), following similar trends to the TCC and ICC data. Starting with a concentration of 80.8 MPN/L in the water on day 1, culturable *L. pneumophila* rapidly established in the biofilm, with a median concentration of 8.6 × 10^1^ MPN/cm^2^ after week 1. Peak median *L. pneumophila* biofilm counts were reached after 4 weeks, with a median value of 3.1 × 10^4^ MPN/cm^2^ (*n* = 6). The concentration then decreased to a median of 1.8 × 10^3^ MPN/cm^2^ (*n* = 6) at the end of the experiment (Fig. 2a). Digital droplet polymerase chain reaction (ddPCR) data showed a similar trend, albeit with lower median concentrations from weeks 1 to 5, which could be due to losses during the DNA extraction procedure. The less pronounced decrease between weeks 6 and 8 could be because quantitative PCR detects culturable, non-culturable and dead bacteria (Fig. 2b). The minimum percentage of *L. pneumophila* gene copies (gc) (relative to *Legionella* spp.) detected was 55%, and the median percentage over all samples was 95% (Fig. 2b), suggesting that *L. pneumophila* accounted for the majority of *Legionella* spp. in the biofilms. Moreover, the maximum percentage of *L. pneumophila* (relative to ICC) was 5.9%, measured after 3 weeks (Supplementary Fig. 2). Furthermore, we found that *L. pneumophila* established on the coupons in a spatially homogenous (Supplementary Fig. 3) and lasting manner (Supplementary Fig. 4). In the water phase, *Legionella* spp. concentrations increased over the first 2 weeks of sampling and then reached a stable concentration with a slight decrease toward the end of the experiment (Supplementary Fig. 5).

### The bacterial community continuously changed

**Fig. 3:**
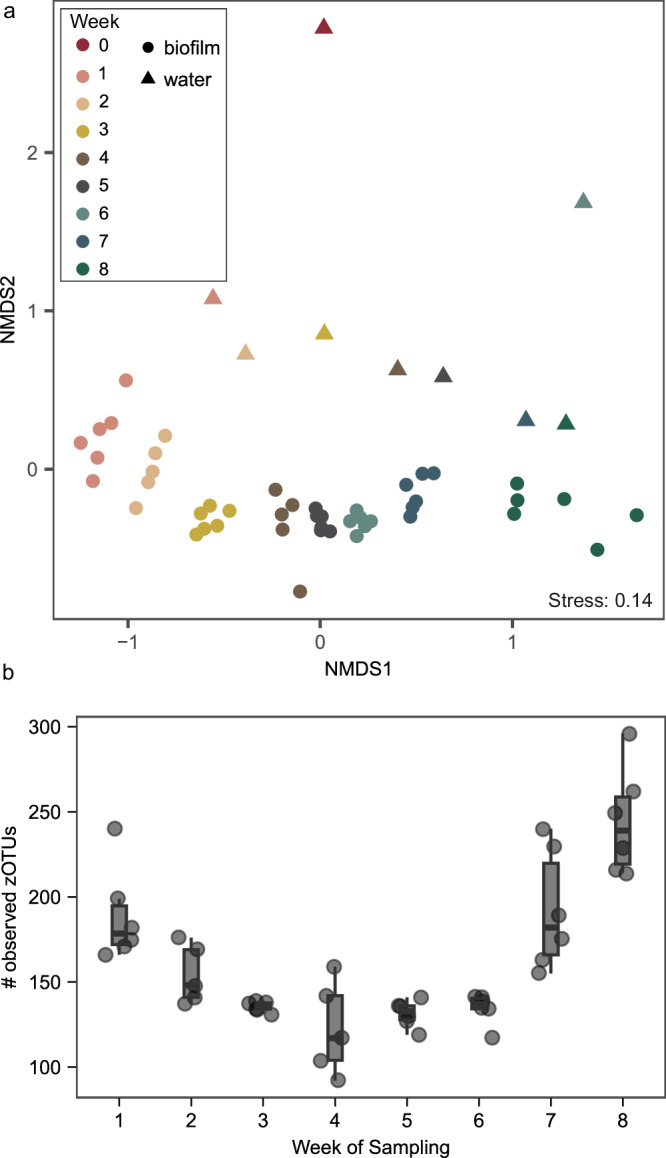
Change of the biofilm bacterial composition over time. **a** Non-metric multidimensional scaling (NMDS) plot based on Bray–Curtis dissimilarities derived from water (triangles) and biofilm (circles) samples (stress = 0.14, *n* = 6 biofilm samples and *n* = 1 water sample per timepoint, except on weeks 2 and 4 where *n* = 5 biofilms due to failed sample amplification during 16S sequencing library preparation). PERMANOVA results indicate that community composition varied significantly depending on the week of sampling (*p* < 0.001). Further pairwise comparisons between samples of each week compared to the previous week were also all significant (*p* < 0.01). **b** Total number of observed zOTUs in biofilms at each sampling point. The box shows the median and interquartile range (25th and 75th percentiles), and the whiskers show the largest value within 1.5 times the interquartile range above the 75th percentile and 1.5 times the interquartile range below the 25th percentile. Significant alpha diversity differences were found between weeks 2 and 3 and weeks 6 and 7 (*p* < 0.05). Dots are individual data points.

16S rRNA gene amplicon sequencing showed that the biofilm bacterial communities continuously changed over the 8-week period (Fig. 3a). The non-metric multidimensional scaling (NMDS) plot based on Bray–Curtis dissimilarities shows a progressive change of both biofilm and water samples. Biofilm replicate samples clustered together, indicating similar microbial composition (Fig. 3a). Water samples were distinct from biofilm samples, but the relative distance reduced over time, suggesting that the water and biofilm microbiome composition became increasingly similar. The biofilm microbial community was relatively diverse, with a median of 189 zOTUs observed after 1 week. The number of observed zOTUs in the biofilms first decreased to a median of 123 (*n* = 5) and then increased again to a median of 244 (*n* = 6) in week 8 (Fig. 3b). This trend is inverse to the TCC and ICC trends (Fig. 1). Inverse Simpson indexes showed an opposite trend when compared to the number of observed zOTUs. Inverse Simpson indexes increased until week 5, suggesting higher evenness of the community. After week 5, a slight decrease in the inverse Simpson index was observed, indicating that the newly immigrated taxa had low abundance (Supplementary Fig. 6). In the water phase, microbial diversity decreased considerably after only 1 week, with 1662 observed zOTUs on day 0 and 205 observed zOTUs after week 1 (Supplementary Fig. 7). While high diversity at the start of the experiment could in part be due to the aggregated samples (9.5% of the water coming from other contaminated outlets at the start, see “Experimental set-up” section) used to inoculate the water bath, this decrease suggests selection and growth after 1 week.

### Identifying biofilm formation pioneers

**Fig. 4:**
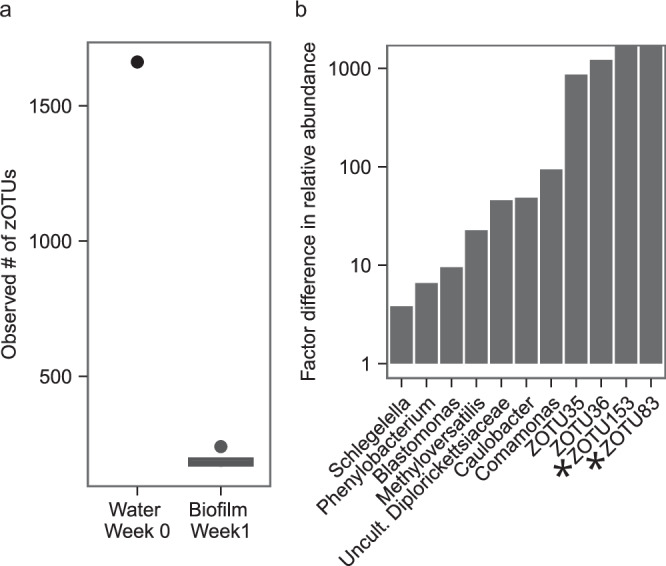
Biofilm formation pioneers. **a** Number of observed zOTUs in the water phase at the start of the experiment (*n* = 1) and in the biofilms after one week (*n* = 6). **b** Factor difference in relative abundance between the microbial community in the water at the beginning of the experiment (*n* = 1) and median relative abundance in biofilms after 1 week (*n* = 6). The asterisk (*) indicates non detection of the zOTU in the water sample at the beginning of the experiment.

A comparison of the microbial diversity and relative abundance of zOTUs in the water phase on day 0 with that in the biofilms after week 1 enabled the identification of biofilm pioneers, which we defined as biofilm members with high abundance at the beginning of biofilm formation on a new material. The observed number of zOTUs in the biofilms after 1 week (median: 189 zOTUs) was lower than the number of zOTUS in the water phase at the start of the experiment (1677 zOTUs) (Fig. 4a), suggesting selective attachment and/or growth on the EPDM coupons within the 1st week. The change in relative abundance of the most prominent bacteria (median relative abundance ≥1% across biofilm samples on week 1 and water sample on week 0) is shown in Fig. 4b. Among these, four unassigned zOTUs were found. These zOTUs were of expected nucleotide length, but could not be assigned to any reference, even when testing multiple databases. zOTU83 and zOTU153 were not detected in the water on the first day but had a median relative abundance of 1.4% and 1.2%, respectively, in the biofilm samples. Unidentified zOTU35 and zOTU36 were enriched in the biofilm after 1 week compared to the water on day 0, with an ~1000-fold increase. zOTUs belonging to the genera *Comamonas*, *Caulobacter*, *Methyloversatilis*, *Blastomonas*, *Phenylobacterium*, *Schlegella* and to the family *Diplorickettsiaceae* have been found to have a 4–50-fold change in relative abundance between start day and week 1.

### Biofilm formation is characterized by succession

**Fig. 5:**
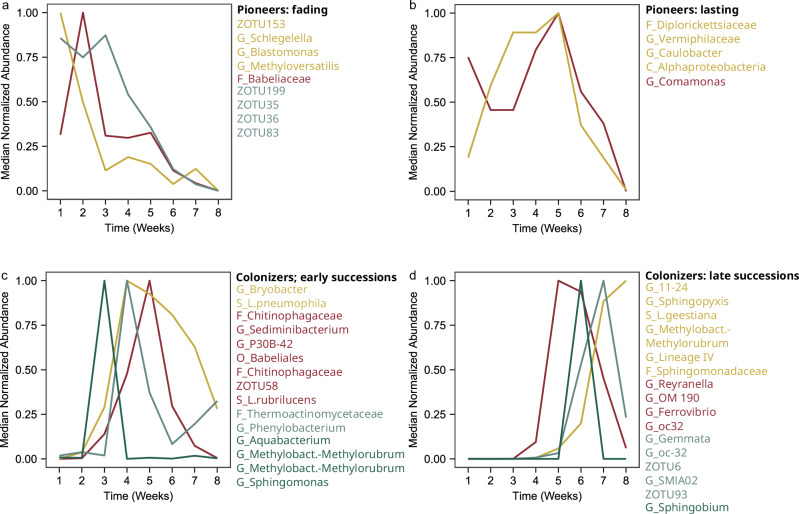
Bacterial community dynamics. The panels depict four different types of dynamics observed in zOTUs abundance. Each line represents the median normalized abundance of a group of zOTUs with similar dynamics, as determined by clustering. The zOTUS are listed to the right of each panel with the color corresponding to their dynamic (see Supplementary Figs. 8 and 9 for additional details). **a** Fading pioneers are defined as bacterial biofilm members already present after one, but whose abundances then rapidly decreased. **b** Lasting pioneers are defined as bacterial biofilm members already present after 1 week and whose concentration increased over the first 5 experimental weeks. **c** Early succession colonizers are defined as members whose abundance starts to increase within 4 weeks since the experiment started. **d** Late succession colonizers are defined as members whose abundance starts to increase after 4 weeks. Taxonomic abbreviations: S Species, G Genus, F Family, O Order, C Class.

To understand which microbial dynamics shaped continuous community shift (Fig. 3a), we calculated the scaled abundance of individual zOTUs over time using FCM TCC^50–52^. Since the number of 16S gene copies vary between different taxa, this does not necessarily present absolute abundance of taxa, but enables the detection of specific temporal trends (Supplementary Figs. 8A–H and 9A–E). Figure 5 depicts the main trends, distinguishing four different types of dynamics: fading pioneers, lasting pioneers, colonizers that contribute to early succession and colonizers that contribute to late succession. For the pioneers (previous section), unassigned zOTUs 83, 153, 35 and 36 together with *Schlegella-*, *Blastomonas-* and *Methyloversatili*s-associated zOTUs were categorized as fading pioneers (Fig. 5a), while zOTUs belonging to the *Comamonas* and *Caulobacter* genera as well as the *Diplorickettsiaceae* family belong to the lasting pioneers. Their presence was sustained until the first 6 weeks of the experiment. Importantly, *Comamonas* and *Caulobacter* were the genera with highest total prevalence across the dataset (for genus level relative abundance information, see Supplementary Fig. 10). Both genera are well known to form aquatic biofilms^53,54^ and it is thus not surprising that they showed a sustained presence, especially at the beginning of the biofilm formation process (Fig. 5b). Other members of the lasting pioneer category included a zOTU belonging to the *Vermiphilaceae* group and an unassigned Alphaproteobacteria. A zOTU belonging to the genus *Phenylobacterium* was present as a pioneer in the biofilms in week 1 already (Fig. 4b) but continued to accumulate to peak after week 4 (Fig. 5c and Supplementary Fig. 9) along with early succession colonizers. These include community members whose scaled abundance peaked between weeks 3 and 5. Among them, some *Legionella* zOTUs were assigned to species level. zOTUs assigned to *L. pneumophila* (prediction confidence: 0.97) and *Legionella rubrilucens* (*L. rubrilucens*) (prediction confidence: 0.88) displayed different dynamics (Fig. 5c). The relative abundance of both *Legionella* species increased rapidly. However, *L. pneumophila* faded slowly, while *L. rubrilucens* faded rapidly following its peak (Supplementary Fig. 8 for details). Colonizers with late succession increased in abundance only after 4 weeks or later. Several of these organisms also show a decreasing trend toward the end of the biofilm formation process. One group, however, contains members in their increasing phase. Among those, zOTUs assigned to *Legionella* *geestiana* (*L. geestiana*) (prediction confidence: 0.82) increased while the two other *Legionella* members of the community decreased (Fig. 5d).

### *Vermamoeba vermiformis* is the most abundant protist

**Fig. 6:**
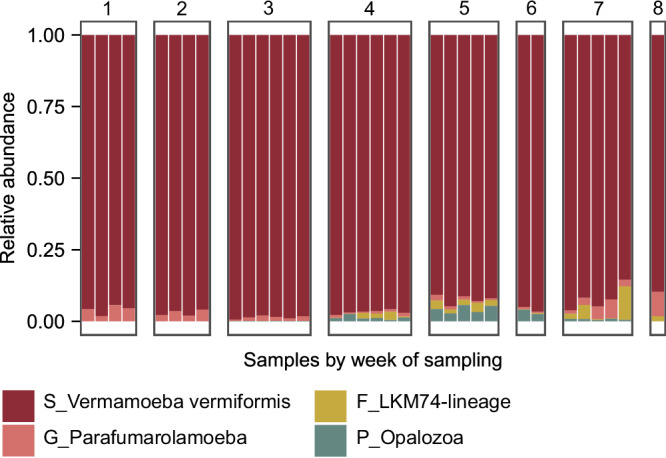
Biofilm eukaryote composition over time. For a given week, each bar represents a replicate sample. Taxonomic abbreviations: S Species, G Genus, F Family, P Phylum.

18S sequencing results indicated a high relative abundance of *V. vermiformis*, a known *Legionella* spp. host (Fig. 6)^26,55^. Interestingly, other potential *Legionella* spp. hosts were also identified in the community, including species from the *Parafumarolamoeba* genus^56^, the LKM74-lineage family^57^ and Opalozoa phylum^58^, albeit with low read count relative abundance (Fig. 6). Overall, our results indicate that protist *Legionella* spp. hosts were prominent in the drinking water biofilm under the specific experimental conditions tested here.

## Discussion

**Fig. 7:**
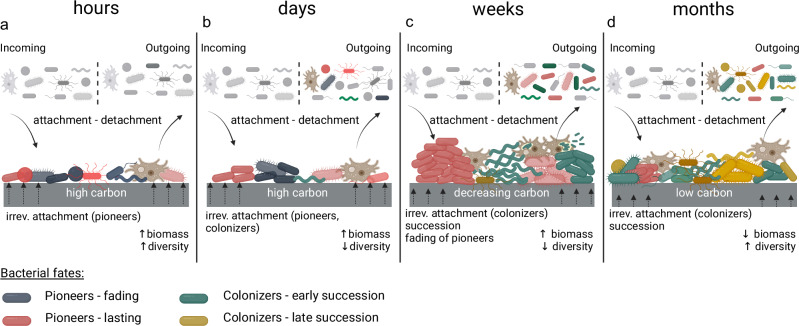
Conceptual model for multispecies biofilm development. The figure illustrates biofilm development over time, highlighting the succession and fates of bacterial pioneers and colonizers. **a** Initial attachment. Within hours, the microbes from the drinking water network attach to the surfaces of new materials, which leaks assimilable carbon. Surface-attached biomass increases. **b** Pioneer growth. High TCC and high diversity after only 7 days are ascribed to both attachment and growth of pioneer microbes. Decrease in diversity is attributed to selective attachment and growth. **c** Colonization, first succession and more growth. Within weeks, biomass continues to increase, diversity continues to decrease and succession occurs where colonizers grow and some of the pioneers start to fade. **d** Colonization, late succession and declining growth. After a month, the amount of biodegradable carbon compounds leaching from the plasticized material will most likely start to decrease. Hence, the biomass on the biofilms starts to decrease likely because of starvation and other stresses.

Here we studied initial drinking water biofilm formation, highlighting *Legionella* spp. and microbial community temporal dynamics. We identified pioneer community members establishing first in the biofilms and described the multiple subsequent community succession events governing biofilm formation. We found that *L. pneumophila* was part of one of the earliest succession of colonizers and that *V. vermiformis*, a known *Legionella* host, was omnipresent in the biofilms. Based on these findings, we propose a conceptual model for biofilm formation by complex multispecies communities on new synthetic polymer material and then discuss this conceptual model in the context of *Legionella* spp. (Fig. 7).

The knowledge gained from our study builds on the universal three-stage biofilm model proposed by Sauer et al.^59^, where we expand on (1) the dynamics of succession of biofilm pioneers and colonizers, (2) the presence of eukaryotes and (3) colonization of specific taxa with pathogenic members (i.e., *Legionella* spp.). Our conceptual model aims at summarizing the main findings of this work in the context of the current literature on biofilm formation. It is based on conditions specific to biofilm formation in building plumbing systems^60^, assuming continual dispersal of diverse bacterial communities^61,62^, high but declining carbon migration from synthetic plumbing materials^63^, and low-nutrient content in the water^64^.

### Initial attachment

Upon commissioning of a new plumbing system (e.g., a brand-new building or a simple shower hose replacement), the diverse microbes from the drinking water network attach to the surfaces of new materials within the first hours^65^. Consequently, surface-attached biomass increases. Attachment is influenced by numerous factors, including substrate properties, hydrodynamics, water chemistry and morphological characteristics of the bacteria (e.g., fimbriae or appendages)^66,67^. Within minutes, the formation of conditioning films comprising organic compounds modifies the surface properties and increases the attachment rates of the microbes^66,68^. This attachment can be reversible^69^, with microorganisms detaching in the suspended phase, or irreversible, leading to a cascade of physiological changes for the microorganisms^59,70^. While we did not study this initial attachment here, the early-stage (i.e., after 1 week) differences in the composition of biofilm samples compared to water samples observed in our study (Fig. 4a) could be explained by selective attachment of pioneer biofilm-forming bacteria and the rapid growth of these pioneers on the available substrate.

### Pioneer growth

We observed high biofilm TCC and relative high diversity (Figs. 1a and 3b) after only 7 days. We attribute this to both attachment and growth of pioneers. These microbes are most likely good biofilm formers in the studied conditions^69,71,72^, characterized by irreversible initial attachment, rapid growth and extracellular polymeric substances (EPS) production. In our study, we identified *Comamonas, Caulobacter, Methyloversatilis, Phenylobacterium Blastomonas* and *Schlegella* as pioneers, along with several unassigned taxa (Fig. 4b). All of the assigned genera have been previously found in water systems^40,41,73–76^. *Comamonas* is an excellent biofilm former and is often a pioneer biofilm member in wastewater treatment processes^54,77^. Members of the *Caulobacter* genus are known biofilm formers and efficiently attach to surfaces^53^, while members of the *Methyloversatilis*^41^, *Phenylobacterium*^78^, *Blastomonas*^79^ and *Schlegella*^41^ genera have been found to be associated with drinking water biofilms. Flexible plastic material used in building plumbing such as EPDM leach up to 0.11 μg/cm^2^ per day total carbon, which is utilized by microorganisms for growth^63,80^. Compared to the water sample on the 1st day, we observed that diversity in the biofilms after 7 days was considerably lower (Fig. 4a and Supplementary Fig. 6). This could be attributed to adaptation of the community to the experimental conditions, such as temperature and residence time, and growth on a new substrate. Moreover, diversity decrease upon growth on carbon leaching material was reported previously^81–84^ and suggests selective growth. Growth of the pioneers, formation of the EPS matrix and changes in biofilm structure create new niches. Since microorganisms are continuously supplied to the system through dispersal by the incoming water, colonizers can start to adhere to the biofilm^72^.

### Colonization, first succession and further growth

During the first weeks, we showed that biomass continues to increase (Fig. 1), diversity continues to decrease (Fig. 3b) and succession occurs where colonizers grow and some of the pioneers start to fade (Fig. 5a–d). Each community member in the biofilm potentially creates new interactions and a new niche leading to the recruitment and growth of additional colonizers^71,85^ while pioneers are replaced through competition, antagonism or predation by protists^86^. The nature of the biodegradable compounds leaching from the substrate might change over time^63^, which likely leads to the creation of new niches.

### Colonization, late succession and declining growth

We observe a decrease in total and intact cell concentrations after week 5 (Fig. 1). This decrease is accompanied by succession. This change in dynamics could be explained by several factors. We hypothesize that after a month, the amount of biodegradable carbon compounds leaching from the plasticized material could start to decrease^80^. This likely affects the community microbial metabolism, leading to a shift in the community composition, and, perhaps, different cell yields or increased detachment in the water phase^87^. Moreover, as in the previous phase, new niches might be created upon the biofilm formation process and the arrival of new colonizers. Interestingly, relative to their own abundance, all pioneers decreased over the 8-week period. This is in line with results from Brislawn et al.^88^, who found that “founder species” in biofilms of hypersaline ecosystems did not maintain high abundance throughout succession and further supports community shifts during the biofilm formation process. Succession could eventually lead to a steady-state composition^89^, which in one drinking water distribution system was shown to occur after more than 500 days^90^.

Interestingly, we did not observe the same level of dynamics when studying the eukaryotic part of the biofilm (Fig. 6). A previous study found that selective forces, time scales and dynamics shaping eukaryotic and bacterial biofilm communities are indeed different^88^, which could explain our results. However, the resolution of the eukaryotic community analysis might be low in our study because of the low biofilm surface area studied, which likely limited the eukaryotic presence. Overall, knowledge on eukaryotic communities in drinking water distribution systems remains scarce^91^; although it is known that they can affect biofilm morphology and create new niches^86^, additional studies focusing on the fate of eukaryotes and their effect on the biofilm community are needed.

The timing described in our conceptual model, as well as the community members and their order of succession reported in our study, are dictated by the experimental conditions. These will vary depending on several factors such as bulk water microbiota composition, nutrient availability, presence of disinfectant, temperature, water retention time and surface to volume ratio. It is well known that the material influences the biofilm composition and the community dynamics observed^78,92^. In this respect, EPDM rubber is one example among many different plumbing materials used in a single building^93^, and known to leach considerable quantities of assimilable organic carbon^63^ leading to biofilm growth and often supporting *Legionella* colonization^48,49^. While our results cannot be generalized for all building conditions, we expect the overall trends in the temporal dynamics and succession to be an inherent part of the biofilm formation and maturation process^39,88^. In this respect, our work proposes a framework to study such dynamics, which can be extended beyond drinking water biofilms.

Finally, the study of other biofilm characteristics such as spatial heterogeneity and the EPS matrix, could enhance the model proposed in this study. Scanning electron micrograph images of our surface-attached biofilms suggest spatial heterogeneity of the biofilm on each coupon (Supplementary Fig. 11). Spatial organization in the biofilms influences how different community members interact, and likely has an impact on the fate of individual organisms in the biofilm^81,94^. Also, microorganisms usually make up <10% of the biofilm dry mass^95^. The rest of the biofilm is made of the EPS matrix and inorganic precipitates^95^, which is the epicenter of microbial interaction (exchange of genetic material, storage of nutrients, cohesion, enzymatic activity).

*L. pneumophila* established quickly in the biofilms and was the dominant *Legionella* spp. found in the biofilms (Fig. 2). Dominance of *L. pneumophila* over other *Legionella* species in biofilms at 37 °C has been documented before^28^. Moreover, in a study testing the biofilm formation potential of 38 different *Legionella* species, *L. pneumophila* had the highest cell number on the surface of different materials at 37 °C^96^. Since this latter study was performed in broth media using pure cultures, the results cannot be directly extrapolated to low-nutrient drinking water environments, but it might indicate that *L. pneumophila* has enhanced biofilm-forming capacity at this temperature compared to other species. In our experiment, indigenous *L. pneumophila* was established in the biofilm within one week and increased in concentration by more than a 100-fold between the 1st and the 3rd week. *L. pneumophila* was not identified as a pioneer but was part of the earliest succession during the biofilm formation process, likely because it can take advantage of the conditions established by pioneer microorganisms. This suggests that *L. pneumophila* is a secondary biofilm colonizer and is consistent with previous studies^15,41^. Similar dynamics with rapid colonization of biofilms by *L. pneumophila* has been documented on other materials^97^. However, to our knowledge, this is the first time that such early establishment of indigenous *L. pneumophila* during biofilm formation on new material is described and quantified over time together with the rest of the microbial community. The omnipresence of *V. vermiformis* throughout the experimental period most probably facilitated the successful colonization and multiplication of *Legionella* spp. within biofilms (Fig. 6). Among amoebae, *V. vermiformis* is ubiquitous in drinking water systems and has been reported as a *Legionella* spp. host multiple times^25,28,41,45^. It is likely that the amoeba easily grazes on the new/young biofilms, thus providing ample nutrients for intracellular *Legionella* growth.

Two other *Legionella* species (*L. rubrilucens* and *L. geestiana)* colonized the biofilm with a relative abundance above our threshold of 1%. Co-occurrence of *Legionella* species in building plumbing systems has been documented previously^8^. In our study, while *L. rubrilucens* was also part of the early succession, *L. geestiana* was a late colonizer. These results demonstrate that different *Legionella* species can colonize the same environment and that they can show different behavior when it comes to their dynamics during biofilm formation (Fig. 5c, d).

Importantly, the increase in *Legionella* spp. concentrations in the biofilms was directly mirrored in the suspended phase of our system (Supplementary Fig. 5). Once *Legionella* spp. established in the biofilm, the concentrations in the suspended phase remained high and stable (in the order of 10^4^ gc/mL). This indicates that the biofilm acts as a reservoir of *Legionella* spp., which are then released in the planktonic phase and can be carried away to new environments, increasing the risk of infection. In this context, the identification of pioneer organisms helping *Legionella* spp. to establish in the biofilm could help to develop strategies interfering with the pathogen’s colonization of biofilms.

## Methods

### Experimental set-up

Biofilms were grown on coupons in a closed water bath (20 L) fed by non-chlorinated municipal tap water (Dübendorf, CH) (Supplementary Fig. 12). The water bath was fitted with a Plexiglass scaffold holding up to 23 biofilm coupon racks, each holding up to 22 individual coupons. Homogeneous conditions were created with baffles and a dedicated recirculation pump (0.5 L/min; Liquiport, KNF). Influent water was dosed continuously (260 mL/h; residence time = 3.5 days) and mixed water was simultaneously removed, using peristaltic pumps (Watson-Marlow). Prior to the experiment start, all components were disinfected with a 0.1% hypochlorite solution and rinsed with ultrapure water. Upon startup, the water bath was filled with 20.1 L influent drinking water and 1.9 L drinking water collected from *L. pneumophila* contaminated taps of our research institution to ensure a microbiota consistent with stagnant drinking water conditions including natural occurring *L. pneumophila* (80 MPN/L final concentration). The water bath’s temperature was set to 37 °C and, upon stabilization, six racks containing 22 coupons made of EPDM rubber (Angst & Pfister, 0.2 × 2.6 × 4.3 cm; 25 cm^2^) were inserted in positions A1, B1, C1, A5, B2 and C8 (Supplementary Fig. 12C). Three days later (day 3), the recirculation and peristaltic pumps were started, and thus fresh municipal tap water began to be fed. At weeks 2 and 4, an additional 22-coupon rack was added to the water bath in positions A2 and C2, respectively.

### Biofilm and water sampling

The biofilm attached to the EPDM coupons and 200 mL of water from the water bath were sampled every 7 days. Six coupons were retrieved each time the biofilm was sampled, taking a coupon from the top and middle of three separate racks (A1, B1 and C1). For the spatial and spatio-temporal analysis of *Legionella* spp. in biofilms, 11 eight-week-old coupons of racks A2, A5, B2, C2 and C8 were sampled. The biofilms were dispersed in 25 mL filtered (0.2 µm) tap water using an electric toothbrush (Oral-B) brushing all sides for 1 min and the edges were brushed 15 times back and forth. A new toothbrush head was used for each coupon. A clean coupon processed in the exact same way was added as technical negative control.

### Determination of total (TCC) and intact (ICC) cell counts

FCM was used to quantify TCC and ICC in dispersed biofilm and water samples. Biofilm samples were sonicated (3 × 30 s cycles, 40% power, cooling on ice between cycles) prior to FCM. Samples were diluted in filtered drinking water (0.2 µm, Steriltech) when necessary. In total, 250 µL aliquots were stained with SYBR Green I (SG, Invitrogen; 10,000× diluted in Tris buffer, pH 8) for TCC or SYBR Green–Propidium Iodide (SGPI; SG with additional propidium iodide in a final concentration of 0.3 mM) for ICC. Samples were incubated for 15 min at 37 °C and measured using a CytoFLEX flow cytometer (Beckman Coulter) as previously described^61^. A fixed gating strategy was used for all samples except for the biofilm samples on week 8 due to high background (see example in Supplementary Fig. 13).

### Determination of culturable *L. pneumophila* concentration

Legiolert (IDEXX) was used to quantify culturable *L. pneumophila*. The tests were performed according to the manufacturer’s protocol, diluting the samples in filtered drinking water (0.2 µm, Steriltech) as needed. Each weekly control (filtered drinking water) was negative (<10 MPN/L).

### DNA extraction

DNA extraction from the water and biofilm samples was performed using the FastDNA SPIN kit (MP Biomedicals) with an adapted protocol. Twenty-two milliliters of the dispersed biofilm samples and 97 mL water samples were concentrated onto 0.2 µm PCTE membrane filters (Steriltech). The filters were fragmented with a sterile scalpel and submerged in 294 µL of 1× Tris-EDTA pH 8.0 (100 mM Tris Base, 10 mM EDTA, G-Bioscience) and 6 µL lysozyme (50 mg/mL, Thermo Scientific) and incubated for 1 h (37 °C, 300 rpm). In total, 300 µL of CLS-TC buffer (FastDNA SPIN kit, MP Biomedicals) and 30 µL of proteinase K (20 mg/mL, Thermo Fischer Scientific) were added to the samples followed by incubation for 30 min (56 °C, 300 rpm). FastDNA Spin kit beads and 630 μL chloroform/Isoamyl alcohol (ratio 24:1, Brunschwig) were added to each sample. After vortexing for 5 min, the samples were centrifuged for 10 min (14,000 × *g*, 4 °C). In total, 750 μL of Binding Matrix (FastDNA SPIN kit, MP Biomedicals) was added to the upper aqueous phase. Then, instructions from the FastDNA spin kit were followed. A negative (unused filter) and a positive control (a batch of positive controls was produced from the water phase) were performed alongside each extraction round. All negative controls were below the detection limit for both the Qbit DNA quantification and the ddPCR measurement. The positive controls were used as a process control in the case of failed extraction or failed quantification of *L. pneumophila* and/or *Legionella* spp. by ddPCR, which did not happen in this study.

### Digital droplet PCR (ddPCR) measurements

Gene copy (gc) numbers of *Legionella* spp. (ssrA gene) and *L. pneumophila* (mip gene) were determined using a ddPCR duplex assay^98,99^. When necessary, samples were diluted in DNase-free water. Each 25 μL reaction contained 1X PerfeCT a Multiplex ToughMix 5X (Quantabio), 0.6 μM of ssrA and 0.4 μM of mip gene forward and reverse primers, 0.15 μM of each probe, 100 nM Fluorescein (Sigma Aldrich), and 5 μL of DNA template. Supplementary Tables 2–4 summarize primer and probe sequences, master mix composition and thermocycling conditions. For each master mix prepared, a negative control (Dnase-free water) was included, which was always negative. One ddPCR reaction positive control (genomic DNA of *L. pneumophila*, Philadelphia reference strain ATCC 33152, Centre National de Référence des Légionelles) was included per thermocycling run. A Stilla Geode instrument was used for droplet formation and PCR thermocycling. Chips were read using a mPrism6 analyzer with Crystal Reader software and droplets were analyzed with the Crystal Miner software. The limit of detection (LOD) of 12 gc/reaction (2.4 gc/μL template) has been determined previously^99^.

### 16S and 18S rRNA genes amplicon sequencing

Bacterial and protist communities were characterized using 16S rRNA and 18S rRNA gene amplicon sequencing respectively. Library preparation and Illumina sequencing were performed in collaboration with the Genetic Diversity Centre (GDC; ETH Zürich, Switzerland). A two-step PCR protocol was used to prepare the sequencing libraries. In the first PCR, the V4 region of the 16S rRNA gene and the V9 region of the 18S rRNA gene were amplified using the Bakt_515F—Bakt_805R51 primers^100^ (Supplementary Table 5) and the Euk 1391F and EUK 1510R primers^101^ (Supplementary Table 6), respectively. For 16S rRNA gene amplicon sequencing, 2.5 ng template DNA was used, except for three samples (Supplementary Table 7). For 18S rRNA gene amplicon sequencing, 4 ng template DNA was used except for five samples (Supplementary Table 8). PCR conditions and reagents are summarized in the Supplementary Information (Supplementary Tables 9–11). The PCR products were purified using the Agencourt AMPure beads (Beckman Coulter, Inc.), with a bead-to-PCR product ratio of 0.8. A second PCR was performed to attach specific Nextera v2 Index adapter (Illumina), with conditions and reagents summarized in Supplementary Tables 12 and 13. Purified PCR products were then quantified on a fluorescent microplate reader (Invitrogen) using the BR Qubit Assay (Thermo Scientific). All PCR products were pooled to equimolar concentrations into a 16S and an 18S rDNA library. For the 16S rDNA library, Agencourt AMPure beads were used to concentrate the pool threefold (bead-to-PCR product ratio of 0.7), followed by a reconditioning PCR step (for details, see Supplementary Tables 14–16) and purification. Purity and peak molarity of both libraries were assessed using the High Sensitivity D1000 ScreenTape system (Agilent 2200 TapeStation) with an average amplicon size of 290 base pairs for the 18S library and 441 base pairs for the 16S library. Both libraries were diluted to a final concentration of 2.8 nM and sequenced using the Illumina MiSeq platform using PhiX at 10%. Positive (ZYMO-1 mock community for 16S and *Acanthamoeba castellanii* standard genomic DNA (ATCC) for 18S) and negative controls (nuclease-free water and extraction negative controls) were included throughout the library preparation process. The extraction negative controls were used for the decontamination process (see Data analysis, below). The positive controls all amplified properly. For 16S rRNA gene amplicon sequencing, the microorganisms present in the commercial mock community could be properly identified. Moreover, relative abundance of DNA contaminants in the commercial positive controls was far below the 1% threshold used in the compositional temporal shift analysis. The sequencing data have been deposited in the EMBL Nucleotide Sequence Database (ENA), as described in the “Data availability” section.

### Data analysis

Statistical analyses on TCC, ICC, *L. pneumophila* Legiolert concentrations and *L. pneumophila* and *Legionella* spp. ddPCR concentrations and total number of observed zOTUs were performed using R (v4.1.1) and Rstudio (v2023.06.1). In all cases, a pairwise Wilcoxon rank sum test was performed with a Benjamini–Hochberg correction.

16S and 18S rDNA read pairs were trimmed and merged using usearch (v11.0.667)^102^ and subsequently filtered based on size and quality. UNOISE3 was used to establish amplicon sequencing variants (from here on “zero-radius Operational Taxonomic Units” (zOTUs)) after denoising and chimera removal. Taxonomic assignment was performed using SINTAX classifier^103^ based on SILVA database (v138)^104^ for 16S and the Protist Ribosomal Reference (PR2) database (V4.14.0)^105^ for 18S. Sampling depth (excluding controls) varied between 28,582 and 144,405 reads per sample for 16S rRNA gene amplicon sequencing and between 175,422 and 6,844,032 reads per sample for 18S rRNA gene amplicon sequencing (Supplementary Tables 17 and 18). Downstream analysis of zOTUs was performed using R (v4.1.1) and Rstudio (v2023.06.1). Contaminant sequences were removed using decontam (v1.20.0)^106^ following the “combined” method. For 16S we used a threshold of 0.25 and the extraction negative controls. “Before decontamination” and “after decontamination” NMDS plots and barplots of the biofilm are shown in Supplementary Fig. 14. For 18S a threshold of 0.5 and the library preparation negative controls were employed. The phyloseq (v1.44.0)^107^ and microbiome (v1.22.0)^108^ packages were used for further analysis. 16S sequences were rarefied to even sampling depth before estimating alpha and beta diversities and performing non-metric multidimensional scaling (NMDS). PERMANOVA was used to test the difference in community composition depending on the week of sampling. The compositional change analyses depicted in Figs. 4 and 5 were performed without rarefaction. For the identification of “pioneer bacteria”, the most prominent zOTUS at the start of the experiment (one water sample) and on week 1 (six biofilm samples) were selected (median relative abundance across all water and biofilm samples ≥1%). Water community composition on start date of the experiment was compared to the median relative abundance of each zOTU across all six biofilm samples after one week. For the compositional shift analysis depicted in Fig. 5, zOTUS with an RA ≥ 1% in at least one sample across all biofilm samples of our study were selected in order to retain zOTUs that were detected with a significant abundance at least once. The scaled abundances of these zOTUs were obtained by multiplying their RA with the corresponding TCC value^50^. For each zOTU, a temporal change curve was obtained, by taking the median scaled abundance of the given zOTU for each week of sampling. The zOTUs abundances were then normalized with the highest abundance value for each taxa set to 1 and the lowest value set to 0. Temporal change curves of the normalized median scaled abundance values of each zOTU were clustered based on their correlation (Tsclust package (v1.3.1)^109^) and complete linkage hierarchical clustering, selecting the number of clusters based on the silhouette index estimated through the NbClust package (v3.0.1)^110^. The defined 13 clusters were then manually classified in four different categories (fading pioneers, lasting pioneers, early succession colonizers, late succession colonizers) depending on their temporal pattern characteristics. For the protist community analysis (18S), zOTUs with an RA ≥ 1% and belonging to the Eukaryote Kingdom were selected. zOTUs whose Phylum was unassigned or belonging to Metazoa were filtered out, keeping only protistan genera. The fractions of total reads covered by this filter for each sample are listed in Supplementary Table 19. ChatGPT 3.5 was used to help generate R code for data visualization. The data analysis scripts are provided in the Supplementary Information.

### Reporting summary

Further information on research design is available in the Nature Research Reporting Summary linked to this article.

## Supplementary information

Supplemental material

Reporting Summary

## Data Availability

16S and 18S rDNA gene amplicon sequences are deposited in the EMBL Nucleotide Sequence Database (ENA), under the project accession PRJEB71916. The rest of the datasets generated during and/or analyzed during the current study are available from the corresponding author on reasonable request.

## Code availability

The underlying code for 16S and 18S rDNA gene amplicon sequence analysis for this study is provided with the Supplementary Information.
