# Supplementary material for: Dynamics of drinking water biofilm formation associated with Legionella spp. colonization
Source: NPJ Biofilms Microbiomes. 2024 Oct 6;10:101. doi: 10.1038/s41522-024-00573-x (PMC11455961; doi:10.1038/s41522-024-00573-x)
Supplement: Supplementary file 1 — Supplemental material [file 41522_2024_573_MOESM1_ESM.pdf]

SUPPLEMETARY INFORMATION for

Dynamics of drinking water biofilm formation associated with *Legionella* spp. colonization

Céline Margot[1,2], William Rhoads[1,2], Marco Gabrielli[1], Margot Olive[1], Frederik Hammes[1,*]

[1] Department of Environmental Microbiology, Eawag, Swiss Federal Institute of Aquatic Science and Technology, Dübendorf, Switzerland

[2] Department of Environmental Systems Science, Institute of Biogeochemistry and Pollutant Dynamics, ETH Zürich, 8092 Zürich, Switzerland

* Corresponding author:

Name: Frederik Hammes

Tel.: +41 58 765 5372

Email: frederik.hammes@eawag.ch

Supplementary Table 1: Total organic carbon measured in the water at each sampling point.

Sampling week	TOC
0	2
1	2.4
2	3
3	3.4
4	1.9
5	1.2
6	1.1
7	1.4
8	no value

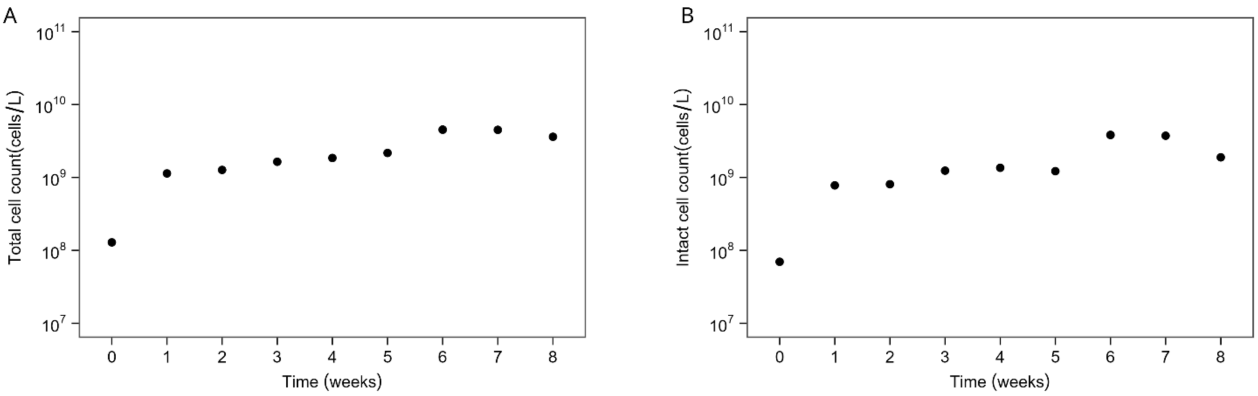

Supplementary Figure 1: Change in total and intact cell concentrations (TCC and ICC) in the water over time. A) Total cell concentrations in the water over the eight-week sampling period. B) Intact cell concentrations in the water over the eight-week sampling period.

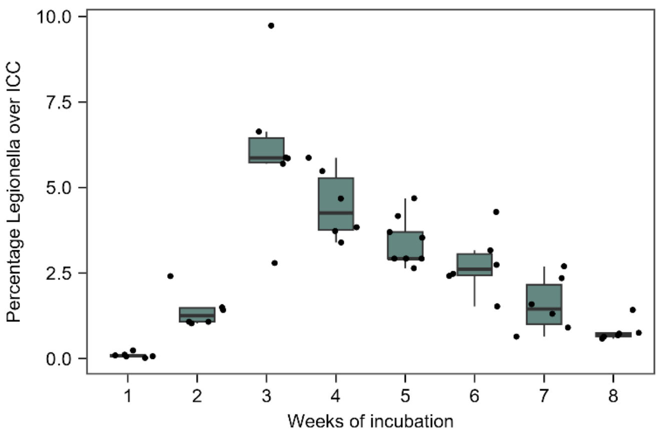

Supplementary Figure 2: Percentage culturable *L. pneumophila* over intact cells over time.

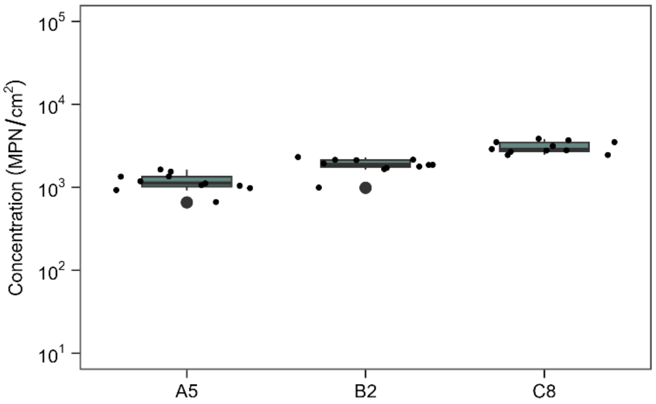

Supplementary Figure 3: Culturable *L. pneumophila* surface densities in eight-weeks old biofilms located in different positions in the experimental system.

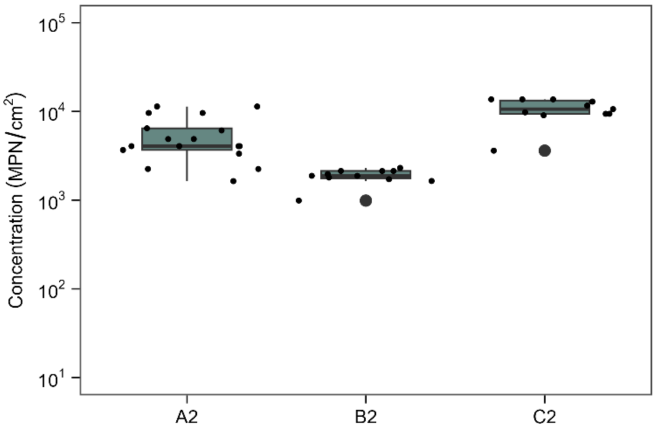

Supplementary Figure 4: Culturable *L. pneumophila* surface densities in eight-week-old biofilms where the racks of EPDM coupons (A2, B2, C2) were introduced at two-week intervals (i.e. different time points) in the experimental system (Inoculation/harvest dates: B2: 2021-04-12/2021-06-07, A2: 2021-04-26/2021-06-23, C2: 2021-05-10/2021-07-05).

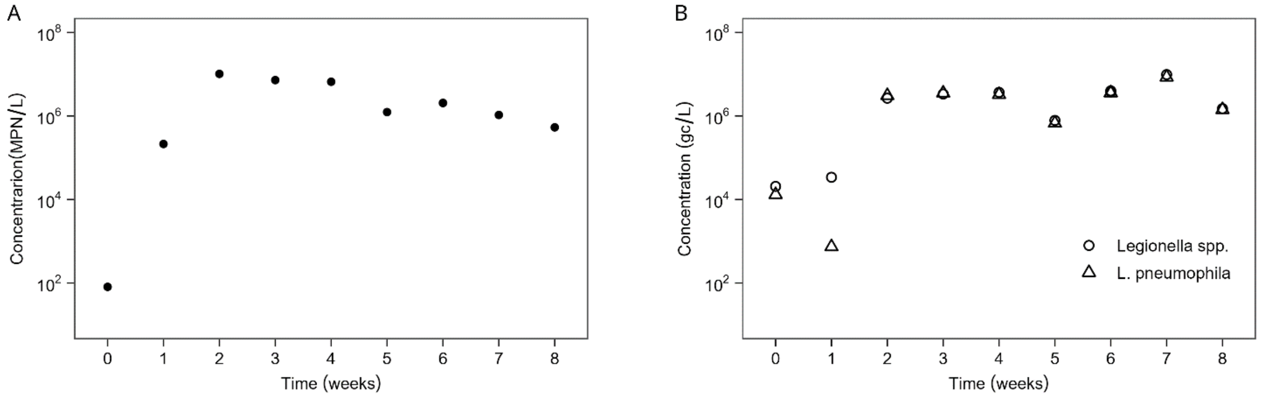

Supplementary Figure 5: *L. pneumophila* and *Legionella* spp. concentrations in the water phase over time. A) Culturable *L. pneumophila* concentrations in the water on each week of sampling. B) *Legionella* spp. and *L. pneumophila* gene copies number in the water on each week of sampling.

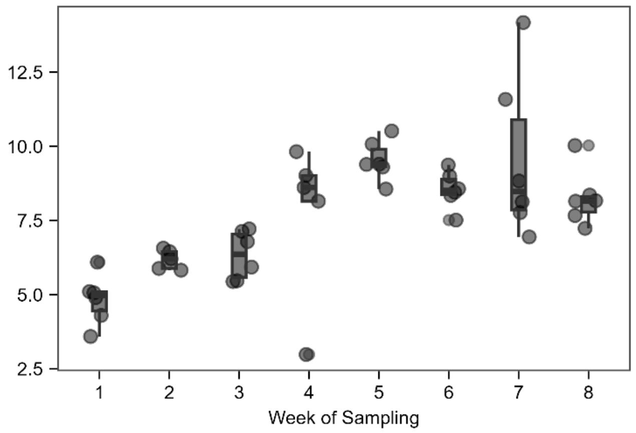

Supplementary Figure 6: Inverse Simpson indexes of the biofilm samples on each week of sampling.

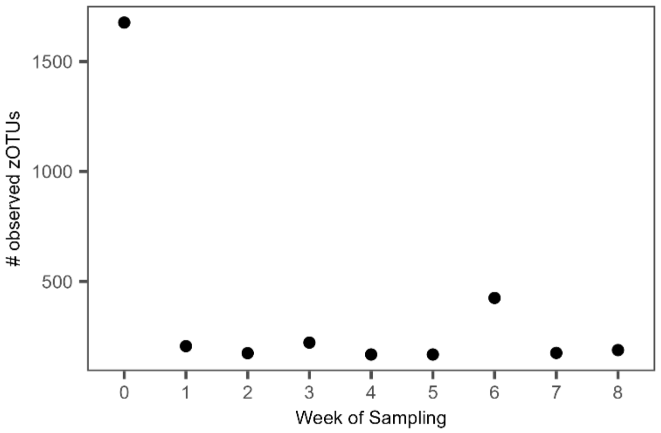

Supplementary Figure 7: Total number of observed zOTUs in the water at each sampling point.

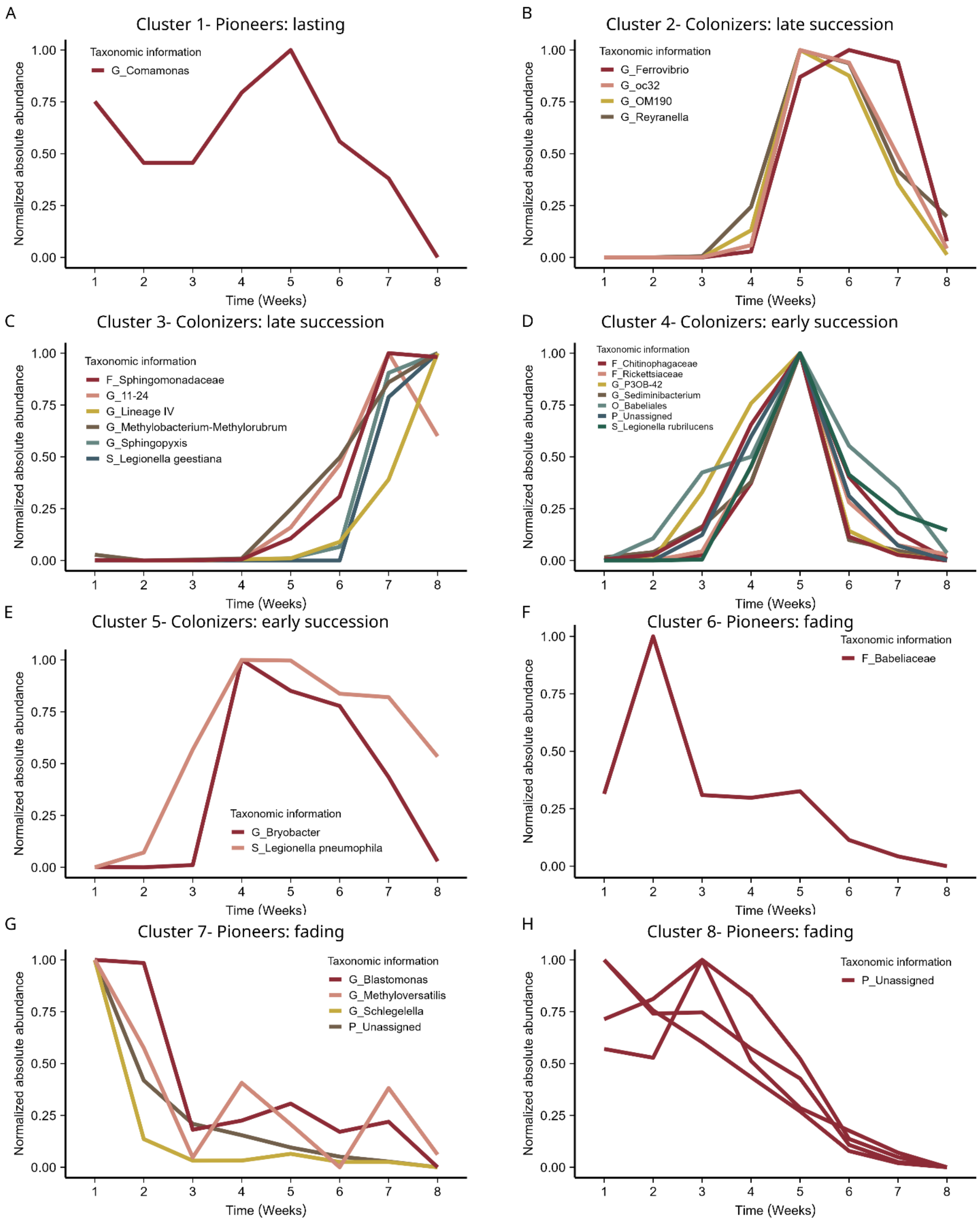

Supplementary Figure 8: Normalized scaled-abundance changes by cluster, Clusters 1 – 8 (see materials and methods for details).

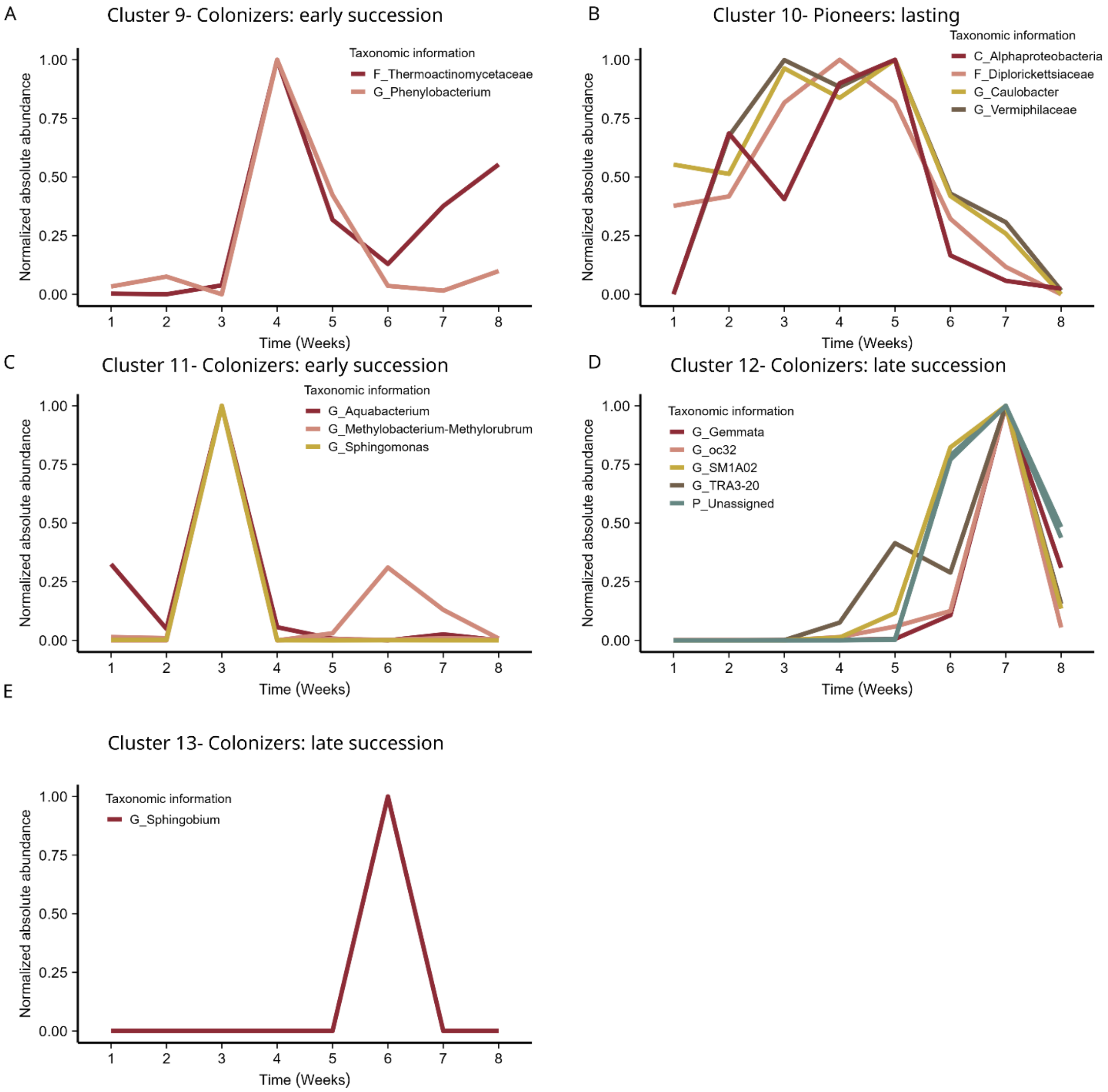

Supplementary Figure 9: Normalized scaled-abundance changes by cluster, Clusters 9 – 13 see materials and methods for details).

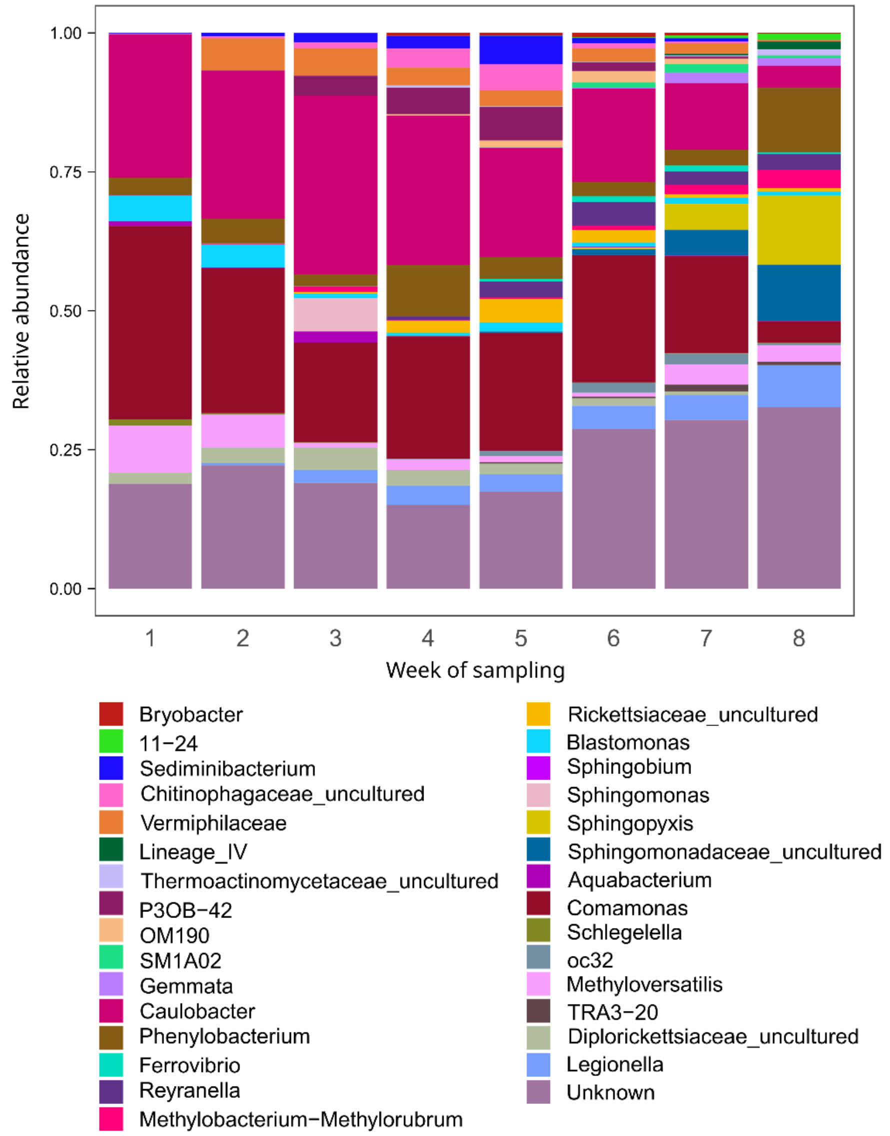

Supplementary Figure 10: Averaged relative abundance per week of sampling for each genus represented in Fig. 5.

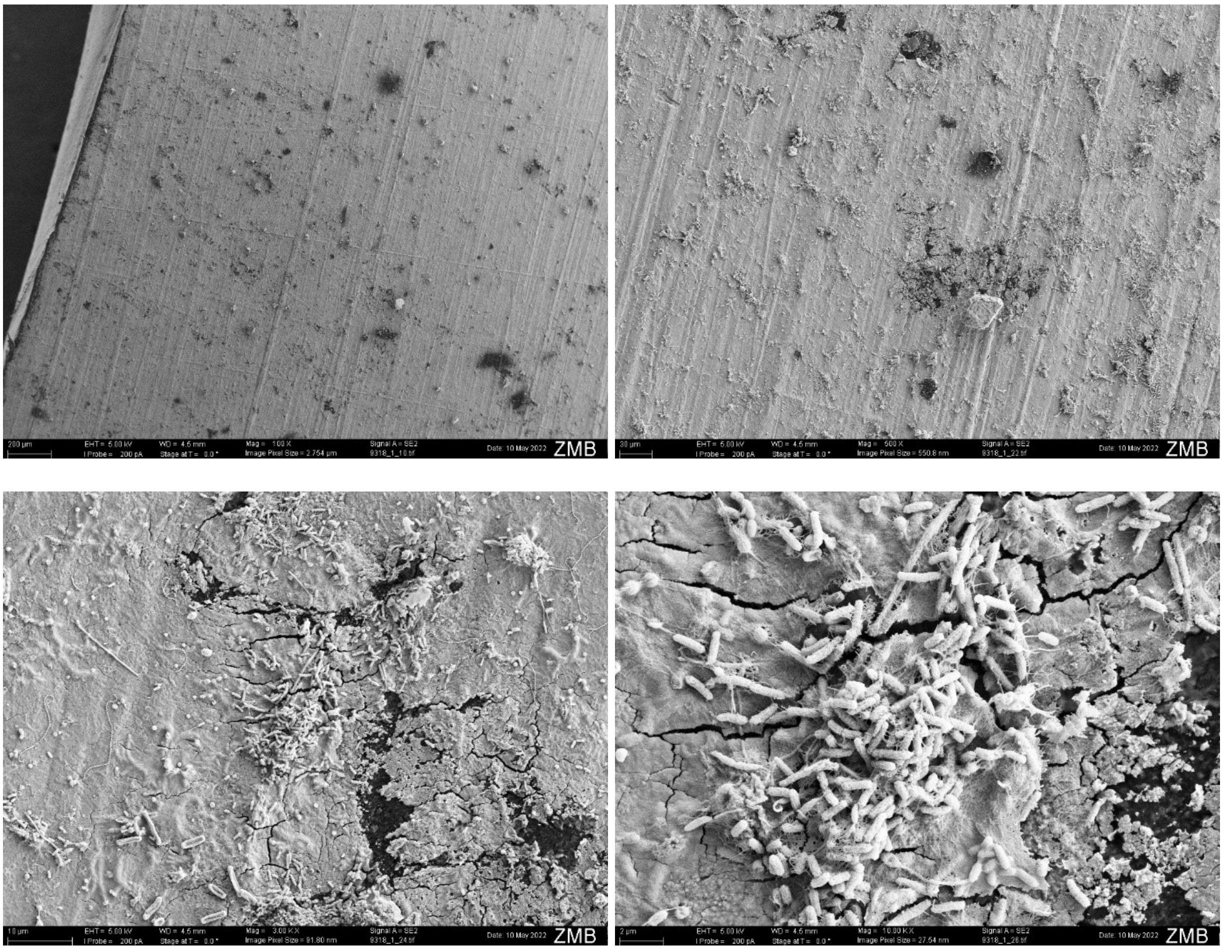

Supplementary Figure 11: Scanning electron micrographs of 12 weeks old biofilm grown in this experiment.

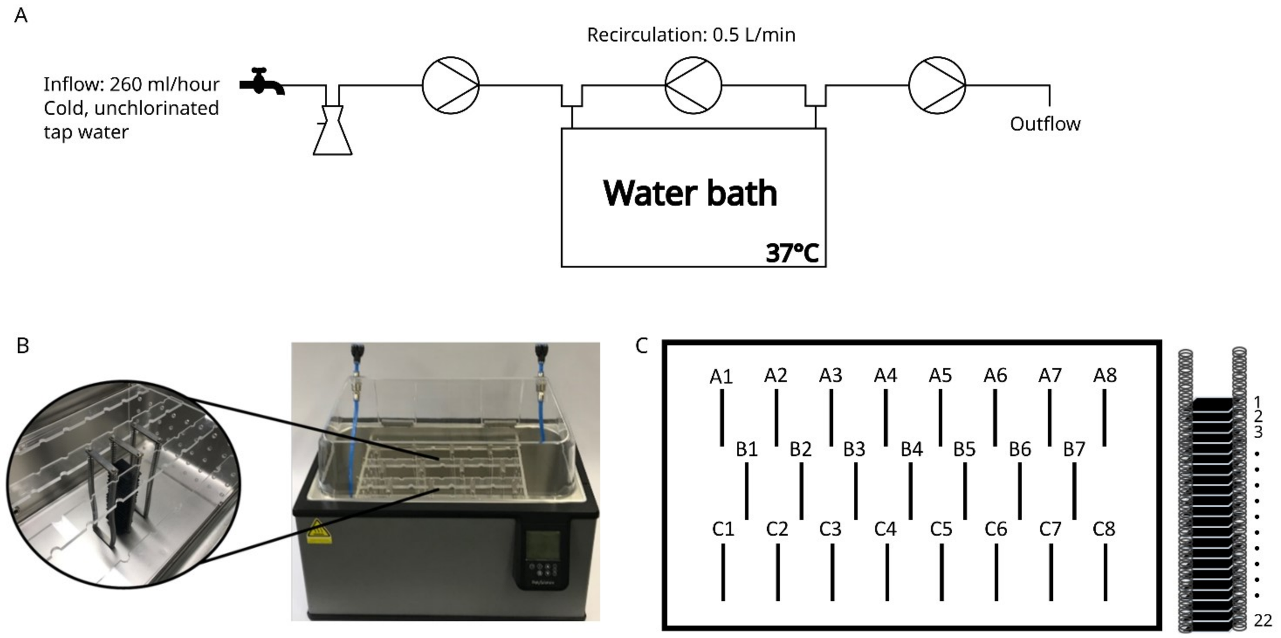

Supplementary Figure 12: Experimental set-up. A: Biofilms were grown in a water bath set at 37°C. Unchlorinated, cold municipal drinking water is dosed continuously in the water bath with a peristaltic pump to the discharge side of the recirculation pump, and mixed water is continuously removed from the suction side of the recirculating pump, respectively, using two-headed peristaltic pumps (Alitea, Watson-Marlow). Water was recirculated at 0.5 L/min, influent water consisted of cold tap water (supplied by Cooperative Water Supply Dübendorf), dosed at 260 mL/hr (residence time = 3.5 days) B: The water bath contains Plexiglas holders, which can hold up to 23 stainless racks each containing 22 coupons (note: picture taken before painting exterior of the lid). C: Top view of the racks' spatial localization in the water bath and side view of a rack with numbering.

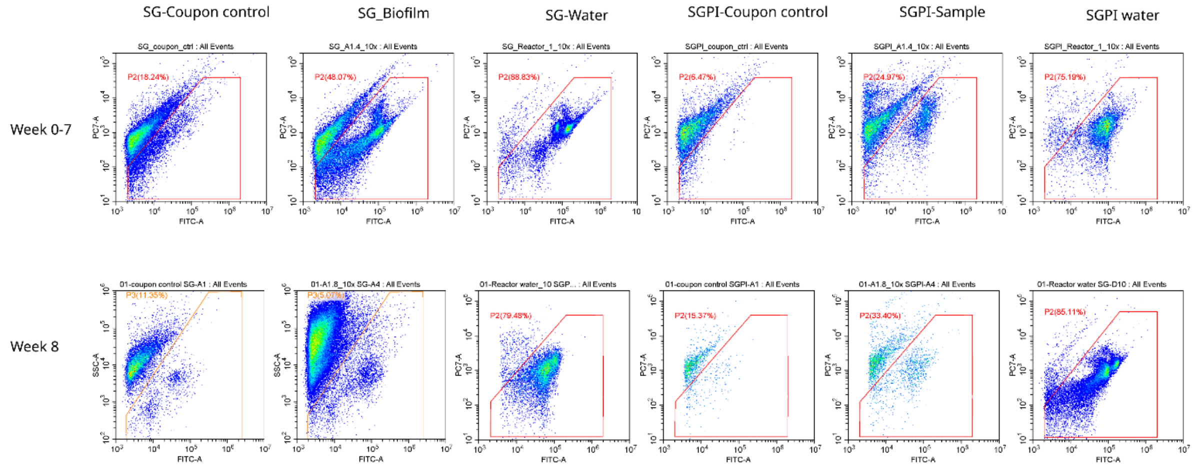

Supplementary Figure 13: Flow cytometry-gating strategy.

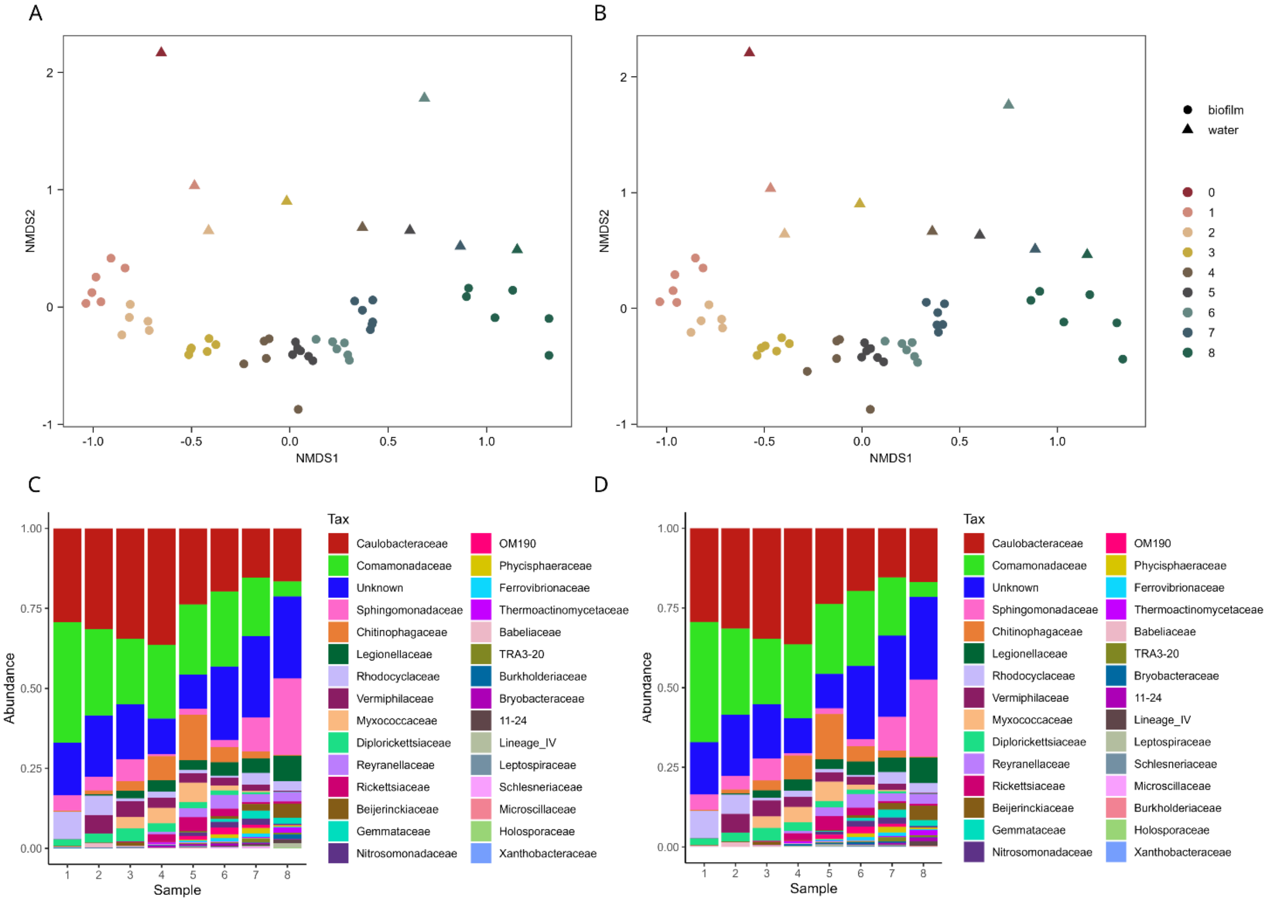

Supplementary Figure 14: Impact of decontamination on community composition. A. NMDS plot representing the biofilm and water samples before the decontamination process (stress: 0.14). B. NMDS plot representing the biofilm and water samples after the decontamination process (stress: 0.14). C. Barplot of the

average family-level composition in the biofilms before decontamination. D. Barplot of the average family-level composition in the biofilms after decontamination.

Supplementary Table 2: Primers and probes used in the duplex ddPCR assay.

Primer/Probe	Sequence
ssrA forward	GGCGACCTGGCTTC
ssrA reverse	GGTCATCGTTTGCATTTATATTTA
ssrA probe	HEX-ACGTGGGTTGCAA-BHQ1
mip forward	TTGTCTTATAGCATTGGTGCCG
mip reverse	CCAATTGAGCGCCACTCATAG
mip probe	FAM-CGGAAGCAATGGCTAAAGGCATGCA-BHQ1

Supplementary Table 3: Master mix composition for duplex ddPCR assay.

Reagent	Final conc.	Vol. for one rx (µL)
RNAse Free Water		10.395
PerfeCTa Multiplex ToughMix 5x	1x	5.4
Fluorescein 1µM	100 nM	2.7
Forward Primer 1 (20µM)	0.6 µM	0.81
Reverse Primer 1 (20µM)	0.6 µM	0.81
Probe 1 (20µM)	0.15 µM	0.2025
Forward Primer 2 (20µM)	0.4 µM	0.54
Reverse Primer 2 (20µM)	0.4 µM	0.54
Probe 2 (20µM)	0.15 µM	0.2025
Total		21.6

Supplementary Table 4: Thermocycling conditions for duplex ddPCR assay.

Step	Settings
Partitioning	12min / 40°C
Enzyme activation	10min / 95°C
45 cycles	15sec / 95°C; 60sec/55°C

Supplementary Table 5: Primers for 16S rRNA gene amplicon sequencing.

Name	Sequence
515F	GTGCCAGCMGCCGCGGTAA

805R	GGACTACHVGGGTWTCTAAT
515F_nex0	TCGTCGGCAGCGTCAGATGTGTATAAGAGACAGTCGTGCCAGCMGCCGCGGTAA
515F_nex1	TCGTCGGCAGCGTCAGATGTGTATAAGAGACAGNTCGTGCCAGCMGCCGCGGTAA
515F_nex2	TCGTCGGCAGCGTCAGATGTGTATAAGAGACAGNNTCGTGCCAGCMGCCGCGGTAA
515F_nex3	TCGTCGGCAGCGTCAGATGTGTATAAGAGACAGNNNTCGTGCCAGCMGCCGCGGTAA
805R_nex0	GTCTCGTGGGCTCGGAGATGTGTATAAGAGACAGGTGGACTACHVGGGTWTCTAAT
805R_nex1	GTCTCGTGGGCTCGGAGATGTGTATAAGAGACAGNGTGGACTACHVGGGTWTCTAAT
805R_nex2	GTCTCGTGGGCTCGGAGATGTGTATAAGAGACAGNNGTGGACTACHVGGGTWTCTAAT
805R_nex3	GTCTCGTGGGCTCGGAGATGTGTATAAGAGACAGNNNGTGGACTACHVGGGTWTCTAAT

Supplementary Table 6: Primers for 18S rRNA gene amplicon sequencing.

Name	Sequence
EUK_1391F _nex0	TCGTCGGCAGCGTCAGATGTGTATAAGAGACAGATGTACACACCGCCCGTC
EUK_1391F _nex1	TCGTCGGCAGCGTCAGATGTGTATAAGAGACAGNATGTACACACCGCCCGTC
EUK_1391F _nex2	TCGTCGGCAGCGTCAGATGTGTATAAGAGACAGNNATGTACACACCGCCCGTC
EUK_1391F _nex3	TCGTCGGCAGCGTCAGATGTGTATAAGAGACAGNNNATGTACACACCGCCCGTC
EUK_1510R _nex0	GTCTCGTGGGCTCGGAGATGTGTATAAGAGACAGTGCCTTCYGCAGGTTCACCTAC
EUK_1510R _nex1	GTCTCGTGGGCTCGGAGATGTGTATAAGAGACAGNTGCCTTCYGCAGGTTCACCTAC
EUK_1510R _nex2	GTCTCGTGGGCTCGGAGATGTGTATAAGAGACAGNNTGCCTTCYGCAGGTTCACCTAC
EUK_1510R _nex3	GTCTCGTGGGCTCGGAGATGTGTATAAGAGACAGNNNTGCCTTCYGCAGGTTCACCTAC

Supplementary Table 7: Samples with added DNA amount < 2.5 ng for 16S rRNA gene amplicon sequencing PCR1.

Sample name	Amount for PCR1 (ng)
B1.2	2.4
B1.8	2.3
C1.8	1.1

T Supplementary Table 8: Samples with added DNA amount < 4 ng for 18S rRNA gene amplicon sequencing PCR1.

Sample name	Amount for PCR1 (ng)
B1.11	2.5
A1.3	< 4
B1.4	< 4
B1.12	1.8

Supplementary Table 9: Reagents for the first step PCR reaction for 16S and 18S rRNA gene amplicon sequencing library preparation.

Reagent	Final concentration	Volume in a 25 µL reaction
2x KAPA HiFi HotStart ReadyMix 1 x 12.5 µl	1x	12.5 µL
Forward primer (10µM)	0.3 µM	0.75 µL
Reverse primer (10µM)	0.3 µM	0.75 µL
PCR grade water		as needed
Template DNA (0.1-10ng)	2.5 ng	as needed

Supplementary Table 10: Thermocycler conditions for the first step PCR of the library preparation for 16S rRNA gene amplicon sequencing library preparation.

<table>
<tr><th>Step</th><th>Temperature</th><th>Time</th><th>Cycling</th></tr>
<tr><td>1</td><td>95°C</td><td>3:00 min</td><td></td></tr>
<tr><td rowspan="3">2</td><td>98°C</td><td>0:20 min</td><td rowspan="3">28 cycles</td></tr>
<tr><td>48°C</td><td>0:15 min</td></tr>
<tr><td>72°C</td><td>0:15 min</td></tr>
</table>

3	72°C	5:00 min	

Supplementary Table 11: Thermocycler conditions for the first step PCR of the library preparation for 18S rRNA gene amplicon sequencing library preparation.

Step	Temperature	Time	Cycling
1	95°C	5:00 min	
2	98°C	0:20 min	26 cycles
	52°C	0:15 min	
	72°C	0:15 min	
3	72°C	5:00 min	

Supplementary Table 12: Reagents for the second step PCR reaction for 16S and 18S rRNA gene amplicon sequencing library preparation

Reagent	Final concentration	Volume in a 25 µL reaction
2x KAPA HiFi HotStart ReadyMix 1 x 12.5 µl	1x	12.5 µL
Nextera XT Index 1 primer (N7XX)	0.3 µM	2.5 µL
Nextera XT Index 2 primer (N7XX)	0.3 µM	2.5 µL
PCR grade water		5 µL
Template DNA (0.1-10ng)	2.5 ng	2.5 µL

Supplementary Table 13: Thermocycler conditions for the second PCR step of the library preparation for 16S and 18S rRNA gene amplicon sequencing library preparation.

Step	Temperature	Time	Cycling
1	95°C	3:00 min	
2	95°C	0:30 min	10 cycles
	55°C	0:30 min	
	72°C	0:30 min	
3	72°C	5:00 min	

Supplementary Table 14: Primer for reconditioning PCR for 16S rRNA gene amplicon sequencing library preparation.

Name	Sequence
Illumina_P7	CAA GCA GAA GAC GGC ATA CGA
Illumina_P5	AAT GAT ACG GCG ACC ACC GA

Supplementary Table 15: Reagents for conditioning PCR for 16S rRNA gene amplicon sequencing library preparation.

Reagent	Final concentration	Volume in a 25 µL reaction
2x KAPA HiFi HotStart ReadyMix 1 x 12.5 µl	1x	3.5 µL
Forward primer (10µM)	0.3 µM	1 µL
Reverse primer (10µM)	0.3 µM	1 µL
PCR grade water		2 µL
Template DNA (0.1-10ng)		12.5 µL

Supplementary Table 16: Thermocycler conditions for reconditioning PCR.

Step	Temperature	Time	Cycling
1	95°C	3:00 min	
2	98°C	0:20 min	4 cycles
	62°C	0:15 min	
	72°C	0:30 min	
3	72°C	1:00 min	

Supplementary Table 17: Number of reads per sample for 16S rRNA gene amplicon sequencing.

Sample	Library Size	Sample Type
N-03	3086	control
N-06	25275	control
C1-16	28582	biofilm
rw-1204	29174	water
rw-3105	30385	water
rw-2604	32768	water
C1-11	34691	biofilm
rw-1705	35325	water
C1-14	35479	biofilm

C1-12	36198	biofilm
rw-1005	37123	water
Mock2	37849	control
N-08	38186	control
A1-02	38542	biofilm
A1-17	38611	biofilm
C1-13	38826	biofilm
A1-13	39484	biofilm
N-02	39853	control
B1-16	39990	biofilm
A1-14	40325	biofilm
A1-15	40463	biofilm
B1-13	41081	biofilm
B1-07	41445	biofilm
C1-07	42062	biofilm
A1-11	42403	biofilm
B1-06	42421	biofilm
rw-0305	42454	water
rw-1904	42705	water
Mock1	44391	control
C1-01	45599	biofilm
A1-18	46029	biofilm
Mock3	46118	control
B1-05	46443	biofilm
A1-12	46831	biofilm
B1-03	47482	biofilm
B1-12	47918	biofilm
rw-2405	48286	water
C1-06	48801	biofilm
B1-18	48814	biofilm
B1-17	49551	biofilm
C1-15	49575	biofilm
C1-04	50020	biofilm
C1-02	50286	biofilm
C1-17	50423	biofilm
A1-16	51820	biofilm
B1-04	52622	biofilm
B1-11	52683	biofilm
C1-05	53143	biofilm
A1-03	53243	biofilm
A1-05	53648	biofilm
B1-15	53990	biofilm
A1-06	56603	biofilm
A1-07	57582	biofilm
C1-03	58743	biofilm
N-09	62415	control
A1-08	66491	biofilm

rw-0706	67472	water
N05	68129	control
B1-01	70728	biofilm
B1-08	73859	biofilm
A1-04	85822	biofilm
rn-2306	98183	control
C1-18	109063	biofilm
N-04	109075	control
N-07	110215	control
A1-01	120059	biofilm
rn-1510	121999	control
N-01	128248	control
N-10	129588	control
rC1-08	144405	biofilm
n-2501	157759	control
n-1510	170387	control
n-2110	187517	control
n-28-10	294017	control
n-2306	345786	control

Supplementary Table 18: Number of reads per sample for 18S rRNA gene amplicon sequencing.

Sample	Library Size	Sample Type
A1-02	263889	biofilm
A1-03	274041	biofilm
A1-04	206050	biofilm
A1-05	241101	biofilm
A1-06	2080421	biofilm
A1-07	233219	biofilm
A1-08	254573	biofilm
A1-11	206015	biofilm
A1-12	202393	biofilm
A1-13	288274	biofilm
A1-14	479250	biofilm
A1-16	211732	biofilm
A1-17	188258	biofilm
B1-01	181857	biofilm
B1-03	391161	biofilm
B1-04	182537	biofilm
B1-05	232514	biofilm
B1-07	6844032	biofilm
B1-11	240268	biofilm
B1-12	183201	biofilm
B1-13	268435	biofilm
B1-14	232343	biofilm
B1-15	178902	biofilm

B1-17	221783	biofilm
C1-03	271480	biofilm
C1-04	189921	biofilm
C1-05	224863	biofilm
C1-07	268875	biofilm
C1-11	295912	biofilm
C1-12	190644	biofilm
C1-13	293695	biofilm
C1-14	193695	biofilm
C1-15	175422	biofilm
NTC-01	143	control
NTC-02	395	control
NTC-03	58	control
NTC-04	215	control
NTC-05	99	control
NTC-06	4405	control
NTC-07	413	control
NTC-08	850	control
NTC-09	87	control
NTC-10	68	control
NTC-11	18	control
NTC-12	33	control

Supplementary Table 19: Fraction of 18S rRNA gene amplicon sequencing reads kept after filtering protists with an RA ⩾ 1%.

**SampleID**	**Fraction of reads kept**
A-1-02	0.419093
A-1-03	0.865658
A-1-04	0.320291
A-1-05	0.278099
A-1-06	0.277484
A-1-07	0.265845
A-1-08	0.009822
A-1-11	0.40197
A-1-12	0.425642
A-1-13	0.396822
A-1-14	0.402779
A-1-16	0.298175
A-1-17	0.241811

B-1-01	0.904917
B-1-03	0.352421
B-1-04	0.369642
B-1-05	0.322555
B-1-07	0.099269
B-1-11	0.383808
B-1-12	0.453671
B-1-13	0.349499
B-1-14	0.269578
B-1-15	0.18835
B-1-17	0.162383
C-1-03	0.326283
C-1-04	0.278692
C-1-05	0.354727
C-1-07	0.187125
C-1-11	0.390572
C-1-12	0.362881
C-1-13	0.269449
C-1-14	0.201416
C-1-15	0.230265

Temporal compositional analysis

Céline Margot

2023-09-04

Introduction

The code below contains all steps performed for the 16S rRNA gene amplicon sequencing analysis and figure production for our paper “Dynamics of drinking water biofilm formation associated with Legionella spp. colonization”.

Setup

Data Import

Load the files including a mapfile containing the DNA concentrations before pooling.

```
#unzip("p734_run220225_16S_Results (1).zip")

otufile.zotu    <- "Sequencing/e_OTU/p734_run220225_16S_ZOTU_Count_Sintax.txt"
mapfile.zotu    <- "Masterfile3.txt"
treefile.zotu   <- "Sequencing/e_OTU/p734_run220225_16S_ZOTU_MSA.tre"
refseqfile.zotu <- "Sequencing/e_OTU/p734_run220225_16S_ZOTU.fa"

d.zotu <- import_qiime(otufilename = otufile.zotu,
                       mapfilename = mapfile.zotu,
                       treefilename = treefile.zotu)
```

```
## Processing map file...
## Processing otu/tax file...
## Reading file into memory prior to parsing...
## Detecting first header line...
## Header is on line 1
## Converting input file to a table...
## Defining OTU table...
## Parsing taxonomy table...
## Processing phylogenetic tree...
##  Sequencing/e_OTU/p734_run220225_16S_ZOTU_MSA.tre ...
```

```
d.zotu
```

```
## phyloseq-class experiment-level object
## otu_table()   OTU Table:         [ 4752 taxa and 272 samples ]
## sample_data() Sample Data:       [ 272 samples by 25 sample variables ]
## tax_table()   Taxonomy Table:    [ 4752 taxa by 7 taxonomic ranks ]
## phy_tree()    Phylogenetic Tree: [ 4752 tips and 4751 internal nodes ]
```

Remove samples which aren’t needed or are part of another experiment

```
# Select ExperimentType: Reactor, LibraryPrep and Extraction
ReactorLibraryExtraction <-
  subset_samples(
    d.zotu,
    ExperimentType == "Reactor" |
    ExperimentType == "Extraction" |
    ExperimentType == "LibraryPrep"
  )

# Remove toothbrush controls
ReactorLibraryExtraction <-
  subset_samples(ReactorLibraryExtraction,
                 !(SampleCategory == "toothbrush" &
                   SampleType == "control")) # remove toothbrush control

# Remove filtered water controls
ReactorLibraryExtraction <-
  subset_samples(
    ReactorLibraryExtraction,
    X.SampleID != "f-0706" &
    X.SampleID != "f-2306" &
    X.SampleID != "f-C2") # remove filtered water controls

# Remove glass control
ReactorLibraryExtraction <-
  subset_samples(ReactorLibraryExtraction,
                 !(SampleCategory == "glass" & SampleType == "control")) # remove gc

# Remove
ReactorLibraryExtraction <-
  subset_samples(ReactorLibraryExtraction,
                 X.SampleID != "A-n" & X.SampleID != "A-p")

# Remove control(s)
ReactorLibraryExtraction <-
  subset_samples(ReactorLibraryExtraction, X.SampleID != "ctrl")

# Remove
ReactorLibraryExtraction <-
  subset_samples(ReactorLibraryExtraction, X.SampleID != "rGDC")

# Remove sample with zero counts after sub-setting
ReactorLibraryExtraction <-
  prune_taxa(taxa_sums(ReactorLibraryExtraction) > 0,
             ReactorLibraryExtraction)
```

After subsetting the data we have to remove zeros.

```
RLE <- prune_taxa(taxa_sums(RLE_PositiveConcentration) > 0, RLE_PositiveConcentration)
RLE <- prune_samples(sample_sums(RLE) > 0, RLE)
```

Sample labels

Now that we have a dataset ready to use, let's first visualize sample read numbers to see the distribution of sequencing sampling depth. For this, we add a column to the dataset which specifies which controls are library prep negatives, which controls are library prep positive, which controls are extraction negative and which controls are extraction positive and finally which control are process control negatives.

Positive controls

We have included three commercial positive controls during the library preparation.

We are using the positive control samples to determine a base level for the counts.

```
Positive          <- subset_samples(RLE, ControlType == "LibraryPrepPos")
Positive          <- prune_taxa(taxa_sums(Positive) > 0, Positive)
Positive_relabund <- transform(Positive, "compositional")
df_positive       <- psmelt(Positive_relabund)

ggplot(df_positive, (aes(x = OTU, y = Abundance))) +
  geom_col() +
  geom_hline(yintercept = 0.01, col = "red", linetype = 2) +
  facet_wrap(~X.SampleID, scales = "free_y") + theme(legend.position = "none")
```

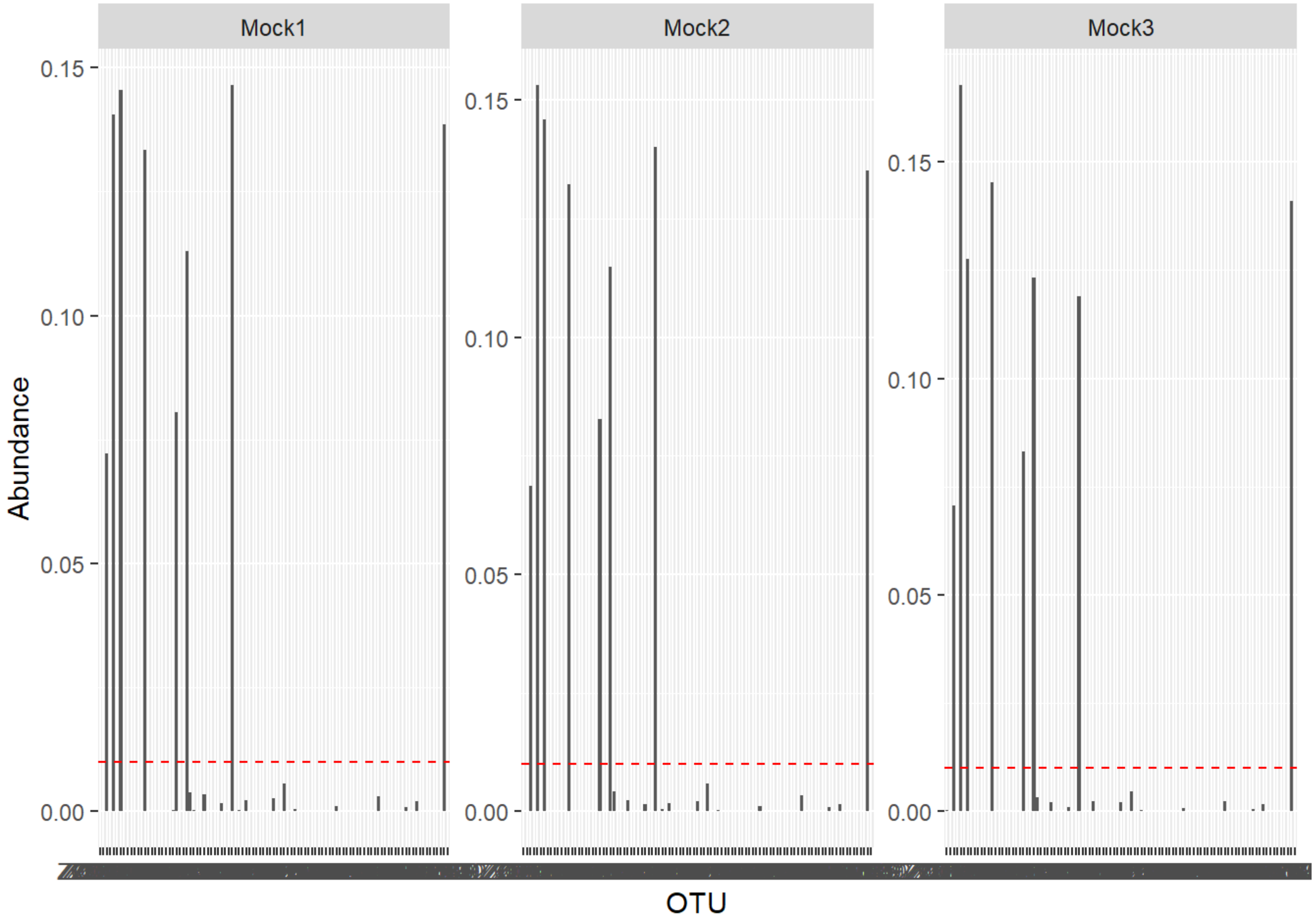

Negative controls

We have 7 DNA extraction negative controls included in our study do determine possible type and level of contamination.

Here we compare the negative controls to the rest of the samples using NMDS.

```
x <- subset_samples(RLE, ControlType == "NotControl" |
                         ControlType == "ExtractionNeg")

x <- subset_samples(x, SampleCategory == "temporal"|
                       ControlType    == "ExtractionNeg" |
                       SampleType     == "water")

# Remove sequencing replicates and samples which aren't part of this study, as well as B1-14
whose amplification failed.
x <- subset_samples(x, X.SampleID != "rA1-05" &
                       X.SampleID != "rrA1-05" &
                       X.SampleID != "rrrA1-05" &
                       X.SampleID != "rB1-02" &
                       X.SampleID != "rrw-C2" &
                       X.SampleID != "rw-2306" &
                       X.SampleID != "rw-C2" &
                       X.SampleID != "rrrw-2405" &
                       X.SampleID != "rrw-2405" &
                       X.SampleID != "rw-0212" &
                       X.SampleID != "rw-0308" &
                       X.SampleID != "rw-0311" &
                       X.SampleID != "rw-0610" &
                       X.SampleID != "B1-14")

ord_x   <- ordinate(x, "NMDS", "jaccard")
```

```
## Square root transformation
## Wisconsin double standardization
## Run 0 stress 0.111955
## Run 1 stress 0.1113946
## ... New best solution
## ... Procrustes: rmse 0.02887813  max resid 0.1578658
## Run 2 stress 0.1093591
## ... New best solution
## ... Procrustes: rmse 0.04685479  max resid 0.260601
## Run 3 stress 0.1110918
## Run 4 stress 0.1113942
## Run 5 stress 0.1091802
## ... New best solution
## ... Procrustes: rmse 0.02688419  max resid 0.1504666
## Run 6 stress 0.1111375
## Run 7 stress 0.1093592
## ... Procrustes: rmse 0.02687788  max resid 0.1507961
## Run 8 stress 0.1275269
## Run 9 stress 0.1186292
## Run 10 stress 0.1120796
## Run 11 stress 0.1206983
## Run 12 stress 0.111395
## Run 13 stress 0.1117132
## Run 14 stress 0.1119559
## Run 15 stress 0.1200349
## Run 16 stress 0.1093591
## ... Procrustes: rmse 0.02714854  max resid 0.150753
## Run 17 stress 0.1117132
## Run 18 stress 0.1143386
## Run 19 stress 0.1132247
## Run 20 stress 0.1208335
## *** Best solution was not repeated -- monoMDS stopping criteria:
##      2: no. of iterations >= maxit
##     18: stress ratio > sratmax
```

```
p_ord_x <- plot_ordination(x, ord_x, type = "samples",
                                   color="ControlType",
                                   shape="SampleType") +
  geom_point(size = 2) +
  theme_few()
```

Sampling effort

```
# Put sample_data into a ggplot-friendly data.frame
df_RLE             <- as.data.frame(sample_data(RLE))
df_RLE$LibrarySize <- sample_sums(RLE)
df_RLE             <- df_RLE[order(df_RLE$LibrarySize),]
df_RLE$Index       <- seq(nrow(df_RLE))

ggplot(data = df_RLE, aes(x = Index, y = LibrarySize, color = ControlType)) +
  geom_point()
```

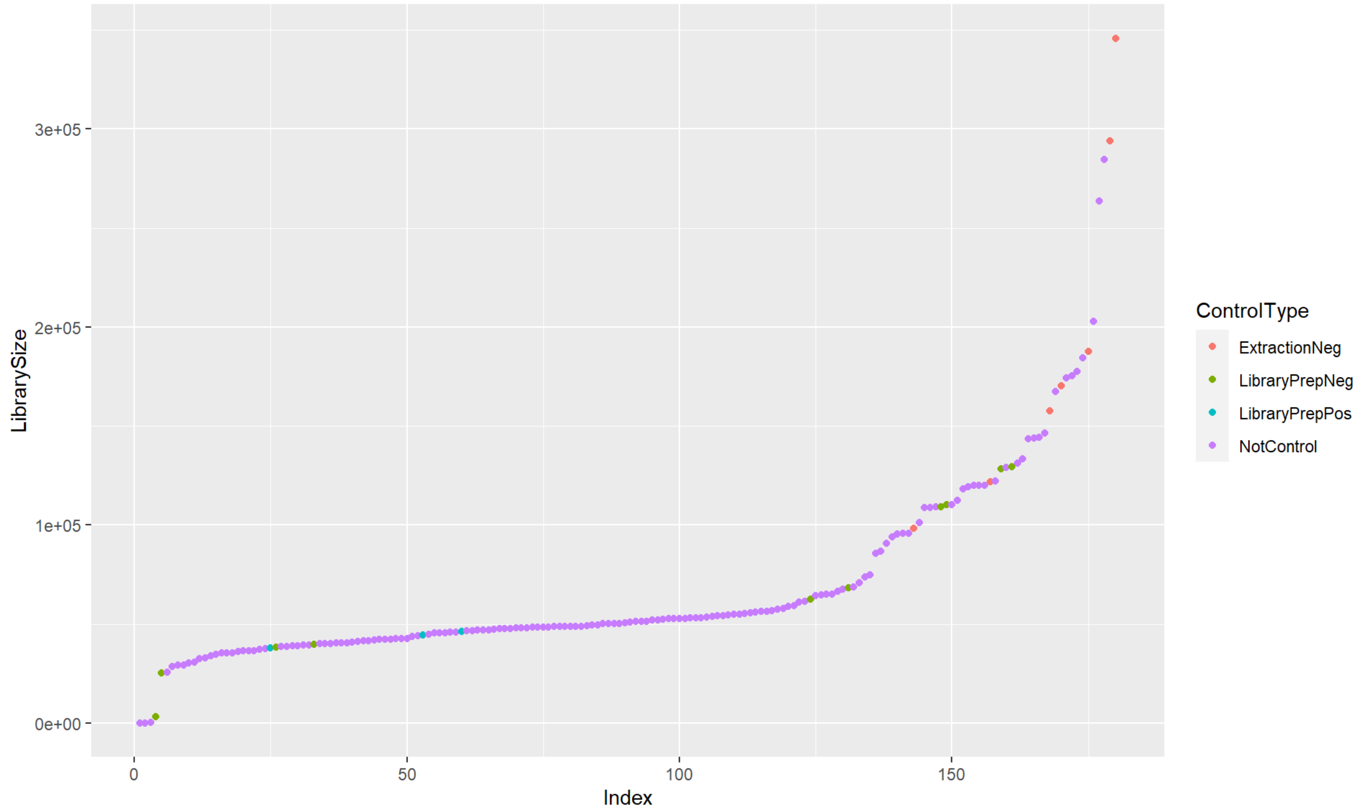

Decontamination

We use the Decontam package to remove potential contaminants using the concentration before pooling and the DNA extraction negative controls.

Compare the frequency, prevalence and combined methods on the whole dataset using the extraction controls as negatives and a threshold of 0.1.

Before proceeding, we want to remove the library preparation negatives and the triplicate mock community and coupon controls.

```
probcols <-
  data.frame(
    row.names = rownames(RLE_f),
    prob.f = RLE_f$p.freq,
    prob.p = RLE_p$p.prev,
    prob.c = RLE_c$p
  )
# TAX2 <- cbind(TAX2[,colnames(TAXannotate)], probcols[TAX2$Id,])
# Crude comparison of frequency and prevalence contaminant assignment
table(probcols$prob.f < 0.1, probcols$prob.p < 0.1)
```

```
##
##          FALSE  TRUE
##   FALSE   3075   165
##   TRUE      95    20
```

```
table(probcols$prob.f < 0.1, probcols$prob.c < 0.1)
```

```
##
##         FALSE TRUE
##   FALSE  3135   54
##   TRUE     72   37
```

```
table(probcols$prob.p < 0.1, probcols$prob.c < 0.1)
```

```
##
##         FALSE TRUE
##   FALSE  3137   33
##   TRUE     70   58
```

Decontamination method comparison

```
TAX <- data.frame(tax_table(bw_decontam))
TAX2 <- cbind(TAX, probcols)
TAXannotate <- data.frame(tax_table(bw_decontam))
rownames(TAXannotate) <- TAXannotate$Id

tt.class <- probcols[!is.na(TAX2$prob.c), ]
TAXann <-
  rbind(
    cbind(tt.class, Score = tt.class$prob.f, Method = "Frequency"),
    cbind(tt.class, Score = tt.class$prob.p, Method = "Prevalence"),
    cbind(tt.class, Score = tt.class$prob.c, Method = "Combined")
  )

TAXann$Method <-
  factor(TAXann$Method, levels = c("Frequency", "Prevalence", "Combined"))

histo <- ggplot(TAXann, aes(x = Score))
histo <- histo +
  geom_histogram() +
  labs(x = 'decontam Score', y = 'Number ASVs') +
  facet_wrap( ~ Method, nrow = 1) +
  theme(legend.position = "bottom")
histo
```

```
## `stat_bin()` using `bins = 30`. Pick better value with `binwidth`.
```

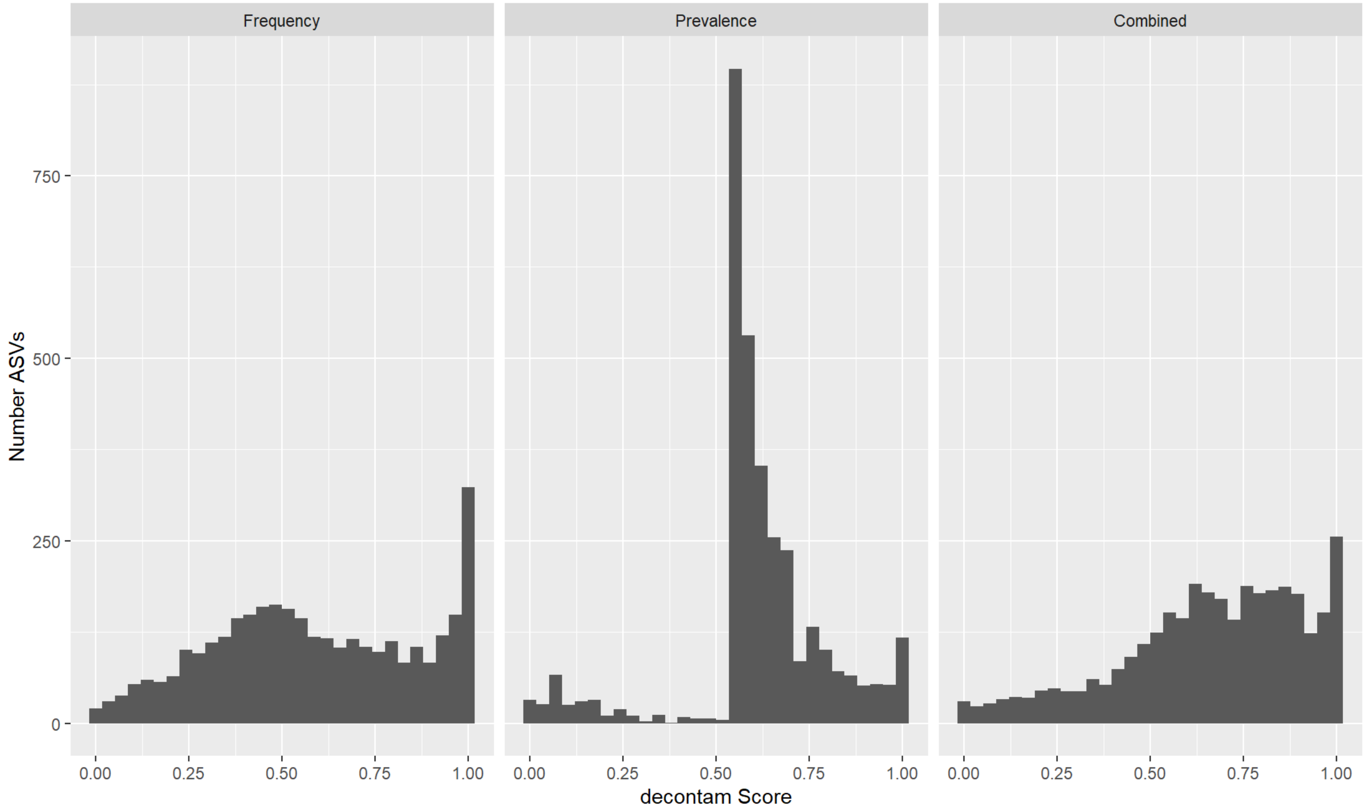

Decontamination with threshold

Decontamination using a decontamination threshold of 0.25 on the whole dataset and the ‘combined’ method. Negative control is the extraction control

```
sample_data(bw_decontam)$is.neg <-
  sample_data(bw_decontam)$ControlType == "ExtractionNeg"
contaminants_bw <- isContaminant(
  bw_decontam,
  method = "combined",
  threshold = 0.25,
  neg = "is.neg",
  conc = "DnaQuant"
)
head(contaminants_bw)
```

	freq <dbl>	prev <int>	p.freq <dbl>	p.prev <dbl>	p <dbl>	contaminant <lgl>
ZOTU3862	3.218412e-06	16	0.6958016	0.7563067	0.8640846	FALSE
ZOTU3398	2.313690e-06	9	0.5043481	0.6632110	0.7008053	FALSE
ZOTU4158	1.955909e-06	10	0.4376468	0.6781320	0.6573005	FALSE
ZOTU2304	5.269204e-06	34	0.9945998	0.9017047	0.9944855	FALSE
ZOTU3863	2.688209e-06	8	0.6644981	0.6477010	0.7932412	FALSE
ZOTU1295	1.405457e-05	12	0.1286363	0.7062817	0.3087662	FALSE

6 rows

```
table(contaminants_bw$contaminant)
```

```
##
## FALSE  TRUE
##  4090   264
```

264 (5.449%) out of 4090 sequences were identified as contaminants.

Let’s remove the contaminants from our phyloseq object

Save (and load) dataset (cleaned, filtered and decontaminated)

```
#save.image("2024-01-15_16SDecontaminated.Rdata")
load("2024-01-15_16SDecontaminated.Rdata")
```

Temporal analysis

Here I begin the analysis that will make up our paper. 6 biofilm samples were collected for 8 weeks, along with a water sample each week.

Data Normalization

We are testing a possible relationship between sampling effort and alpha diversity. We use Bray-Curties dissimilarity.

```
BCdist      <- distance(All_temporal, method = "bray")
librarySize <- sample_sums(All_temporal)
libSizedist <- dist(librarySize)
plot(libSizedist, BCdist)
```

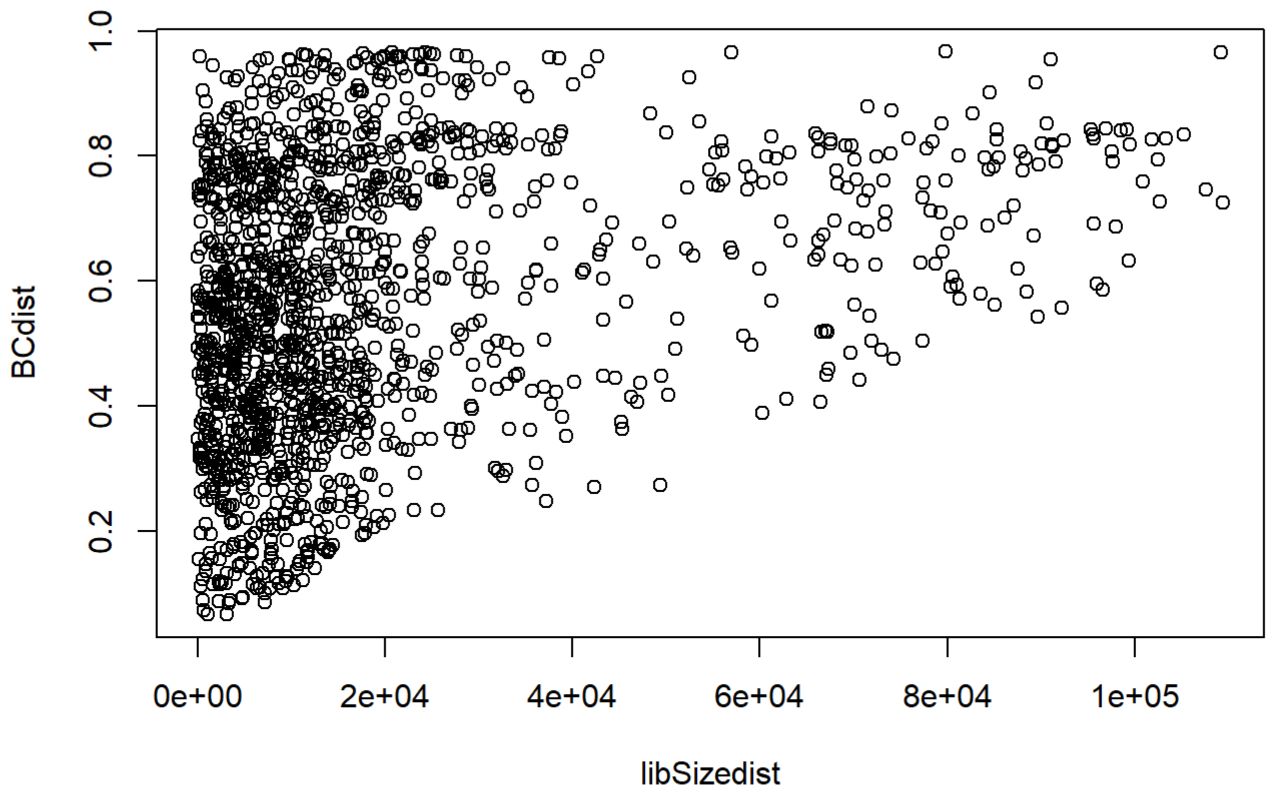

Slight trend which suggests rarefaction would be needed before ordination.

Rarefaction

```
All_temporal_rarefied <-
  rarefy_even_depth(
    All_temporal,
    sample.size = min(sample_sums(All_temporal)),
    rngseed = TRUE,
    replace = TRUE,
    trimOTUs = TRUE,
    verbose = TRUE
  )
```

```
## `set.seed(TRUE)` was used to initialize repeatable random subsampling.
```

```
## Please record this for your records so others can reproduce.
```

```
## Try `set.seed(TRUE); .Random.seed` for the full vector
```

```
## ...
```

```
## 1755OTUs were removed because they are no longer
## present in any sample after random subsampling
```

```
## ...
```

```
#save.image("2024-01-15_16SRarefied.Rdata")
load("2024-01-15_16SRarefied.Rdata")
```

Ordination with Legend

```
custom.col = c(
  "#8D2B36",
  "#CF897A",
  "#DAB489",
  "#C4AA3F",
  "#715F4B",
  "#4B4A4C",
  "#658782",
  "#3f5c6a",
  "#24604D"
)
ord_All_temporal_BRAY <-
  ordinate(All_temporal_rarefied, "NMDS", "bray")
```

```
## Square root transformation
## Wisconsin double standardization
## Run 0 stress 0.1128255
## Run 1 stress 0.1306651
## Run 2 stress 0.136851
## Run 3 stress 0.1245822
## Run 4 stress 0.1490899
## Run 5 stress 0.1285092
## Run 6 stress 0.112605
## ... New best solution
## ... Procrustes: rmse 0.003680608  max resid 0.01755779
## Run 7 stress 0.1193769
## Run 8 stress 0.1273267
## Run 9 stress 0.1103698
## ... New best solution
## ... Procrustes: rmse 0.02584021  max resid 0.1089677
## Run 10 stress 0.1171185
## Run 11 stress 0.120215
## Run 12 stress 0.138179
## Run 13 stress 0.1105153
## ... Procrustes: rmse 0.004930038  max resid 0.02087876
## Run 14 stress 0.1327609
## Run 15 stress 0.1377969
## Run 16 stress 0.1281309
## Run 17 stress 0.1328539
## Run 18 stress 0.1331236
## Run 19 stress 0.1286799
## Run 20 stress 0.1126205
## *** Best solution was not repeated -- monoMDS stopping criteria:
##     20: stress ratio > sratmax
```

```
p_ord_All_temporal_BRAY = plot_ordination(
  All_temporal_rarefied,
  ord_All_temporal_BRAY,
  type = "samples",
  color = "WeekOfSampling",
  shape = "SampleType"
) + geom_point(size = 2)  + theme_few()
print(p_ord_All_temporal_BRAY)
```

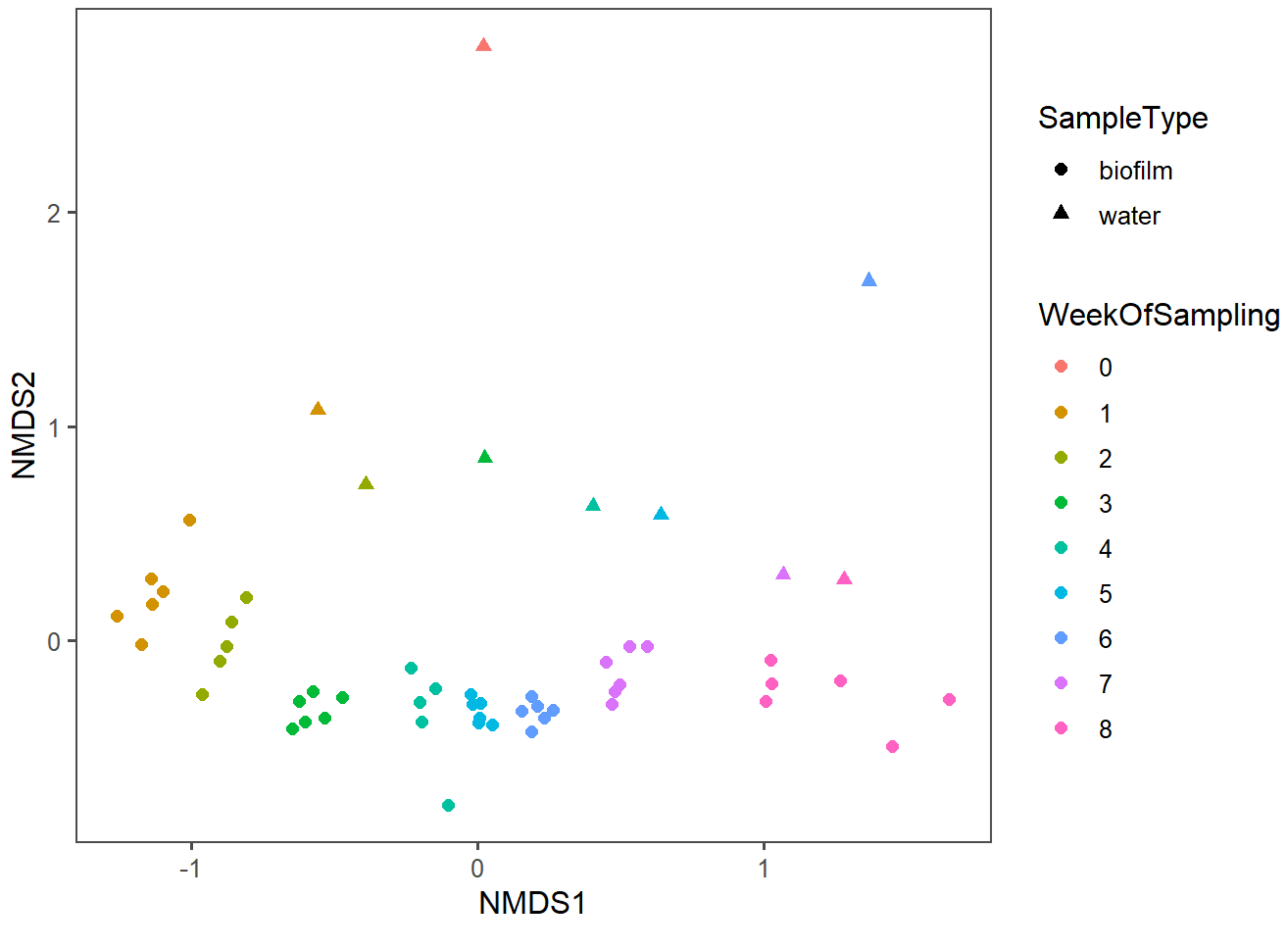


```
# Apply custom colors to the plot and define text sizes
p_ord_All_temporal_BRAY_withL <-
  p_ord_All_temporal_BRAY + scale_color_manual(values = custom.col) +
  theme(
    axis.text    = element_text(size = 7, color = "black"),
    axis.title   = element_text(size = 7, color = "black"),
    axis.ticks   = element_line(color = "black", linewidth = 0.25),
    legend.text  = element_text(size = 7),
    legend.title = element_blank(),
    # Remove the legend title for both legends
    legend.key.size = unit(4, "mm")
  )
print(p_ord_All_temporal_BRAY_withL)
```

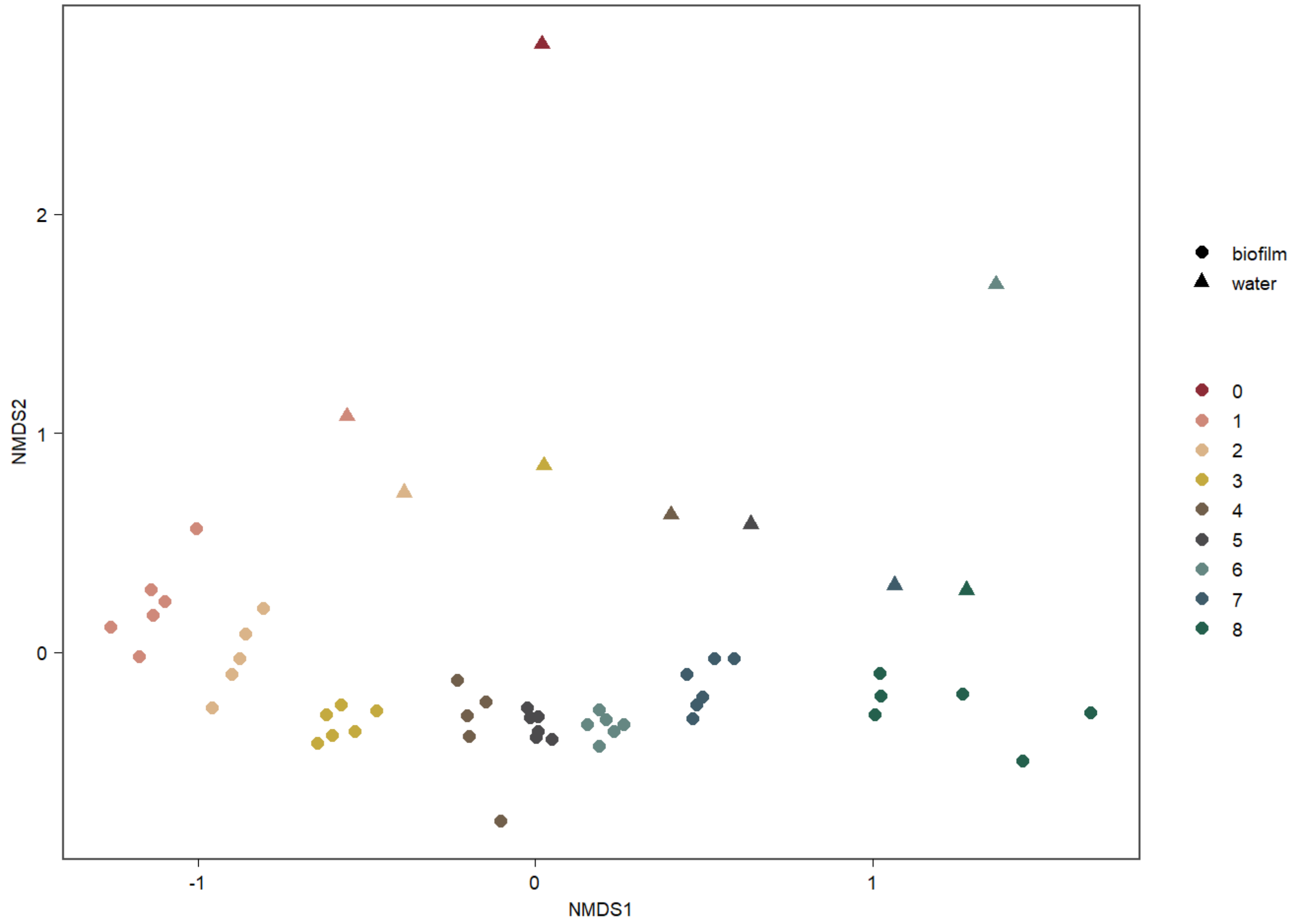


```
ggsave(
  filename = "p_ord_All_temporal_BRAY_withL.pdf",
  dpi = 600,
  p_ord_All_temporal_BRAY_withL ,
  width = 88,
  height = 90,
  units = "mm"
)
```

Ordination without Legend

```
ord_All_temporal_BRAY    <- ordinate(All_temporal_rarefied, "NMDS", "bray")
```

```
## Square root transformation
## Wisconsin double standardization
## Run 0 stress 0.1128255
## Run 1 stress 0.1142986
## Run 2 stress 0.1201463
## Run 3 stress 0.1294683
## Run 4 stress 0.1246006
## Run 5 stress 0.1126509
## ... New best solution
## ... Procrustes: rmse 0.007546591  max resid 0.0346577
## Run 6 stress 0.1100576
## ... New best solution
## ... Procrustes: rmse 0.02625829  max resid 0.1093909
## Run 7 stress 0.1171146
## Run 8 stress 0.1102887
## ... Procrustes: rmse 0.004181568  max resid 0.02570578
## Run 9 stress 0.130465
## Run 10 stress 0.1339186
## Run 11 stress 0.1102886
## ... Procrustes: rmse 0.004176402  max resid 0.02563757
## Run 12 stress 0.1202474
## Run 13 stress 0.1147917
## Run 14 stress 0.130465
## Run 15 stress 0.1203642
## Run 16 stress 0.1174693
## Run 17 stress 0.1329616
## Run 18 stress 0.1105153
## ... Procrustes: rmse 0.009398604  max resid 0.05410876
## Run 19 stress 0.1201449
## Run 20 stress 0.144606
## *** Best solution was not repeated -- monoMDS stopping criteria:
##     18: stress ratio > sratmax
##      2: scale factor of the gradient < sfgrmin
```

```
p_ord_All_temporalr_BRAY <- plot_ordination(All_temporal_rarefied, ord_All_temporal_BRAY, typ
e = "samples", color = "WeekOfSampling", shape = "SampleType") +
  geom_point(size = 2) +
  theme_classic()

# Apply custom colors to the plot and define text sizes
p_ord_All_temporalr_BRAY<- p_ord_All_temporalr_BRAY + scale_color_manual(values = custom.col)
+
  theme_few()+
  theme(
    axis.text = element_text(size = 7),
    axis.title = element_text(size = 7),
    legend.text = element_text(size = 7),
    legend.title = element_blank(),  # Remove the legend title for both legends
    legend.key.size = unit(4, "mm"),
    legend.position = "none"  # Remove the legend
  )

print(p_ord_All_temporalr_BRAY)
```

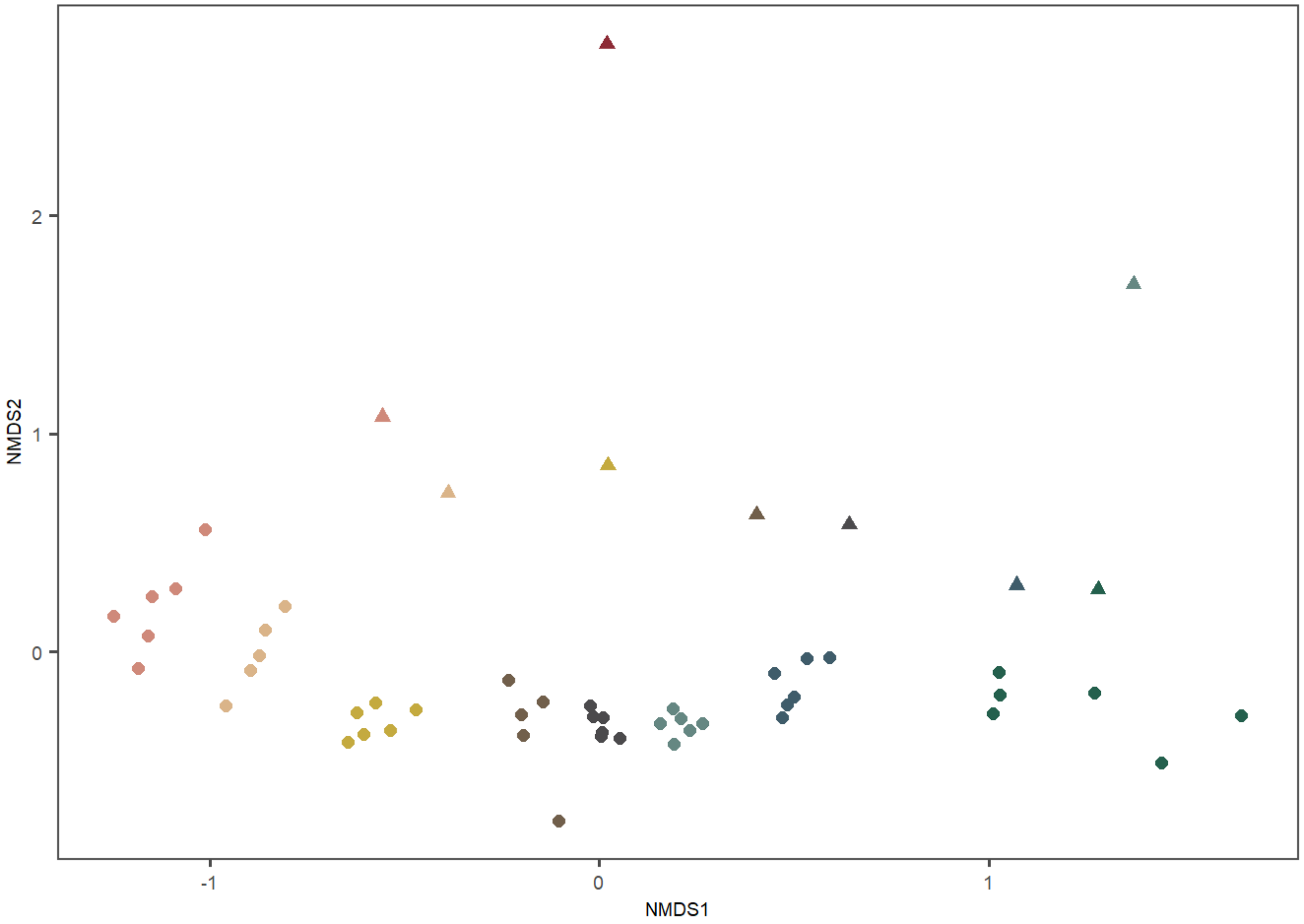


```
# Save the plot
ggsave(filename = "p_ord_All_temporalr_BRAY.pdf", dpi=600, p_ord_All_temporalr_BRAY, width =
88, height = 90, units = "mm")
```

The ordination shows the temporality of the data. Community composition evolves over time. When plotted using Jaccard distance, we obtain a very similar NMDS plot, indicating that it is not only the proportions of different taxa that change, but also the composition.

Alpha diversities

Here, for biofilm and water samples separately, I examine the evolution of richness and evenness in the data over time.

Biofilm samples

Subset biofilm samples and inspect library sizes

```
decontam_biofilm <-
  subset_samples(All_temporal, SampleType == "biofilm")
# Put sample_data into a ggplot-friendly data.frame
temporal_depth <-
  as.data.frame(sample_data(decontam_biofilm))
temporal_depth$LibrarySize <- sample_sums(decontam_biofilm)
temporal_depth  <-
  temporal_depth[order(temporal_depth$LibrarySize), ]
temporal_depth$Index <- seq(nrow(temporal_depth))

ggplot(data = temporal_depth , aes(x = X.SampleID, y = LibrarySize, color = WeekOfSampling))
+
  geom_point()
```

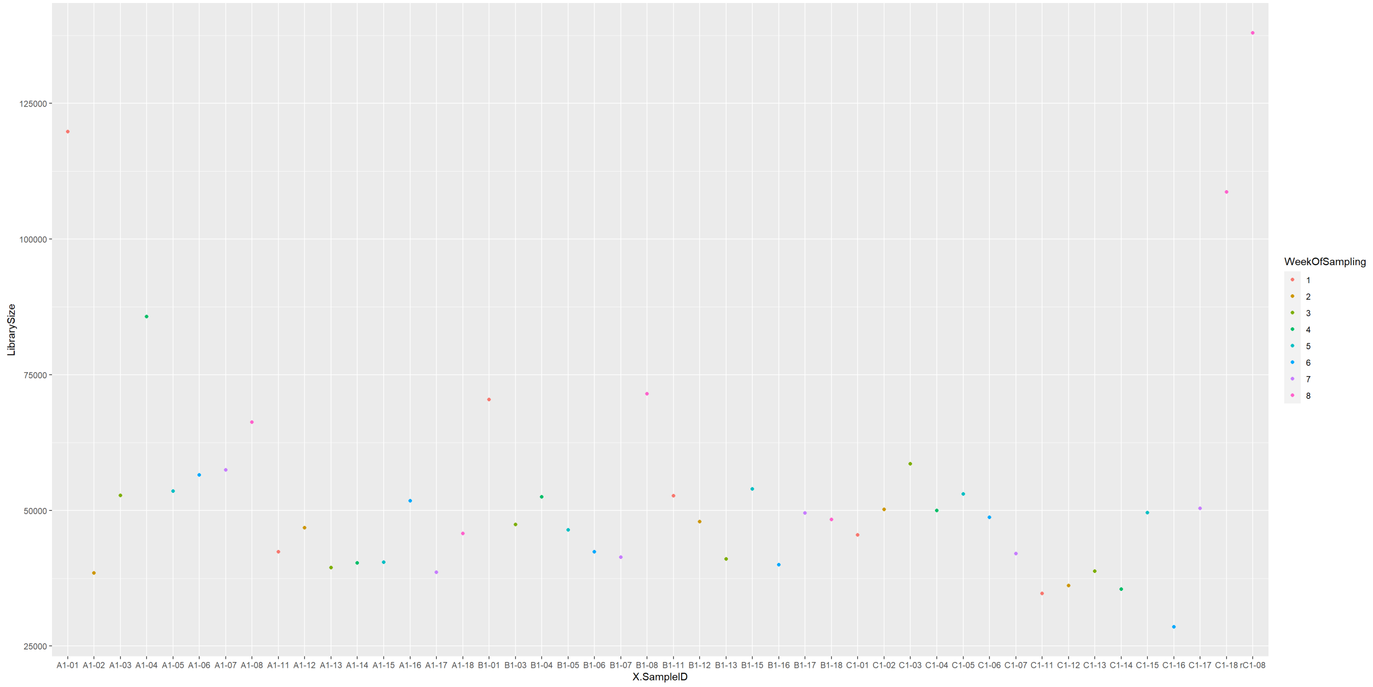

4 samples with high sequencing depth.

Use rarefied dataset for alpha diversity calculations.

```
biofilm        <- subset_samples(All_temporal_rarefied, SampleType == "biofilm")
biofilm_pruned <- prune_taxa(taxa_sums(biofilm) > 0, biofilm)
plot_richness(biofilm_pruned,  x = "WeekOfSampling")
```

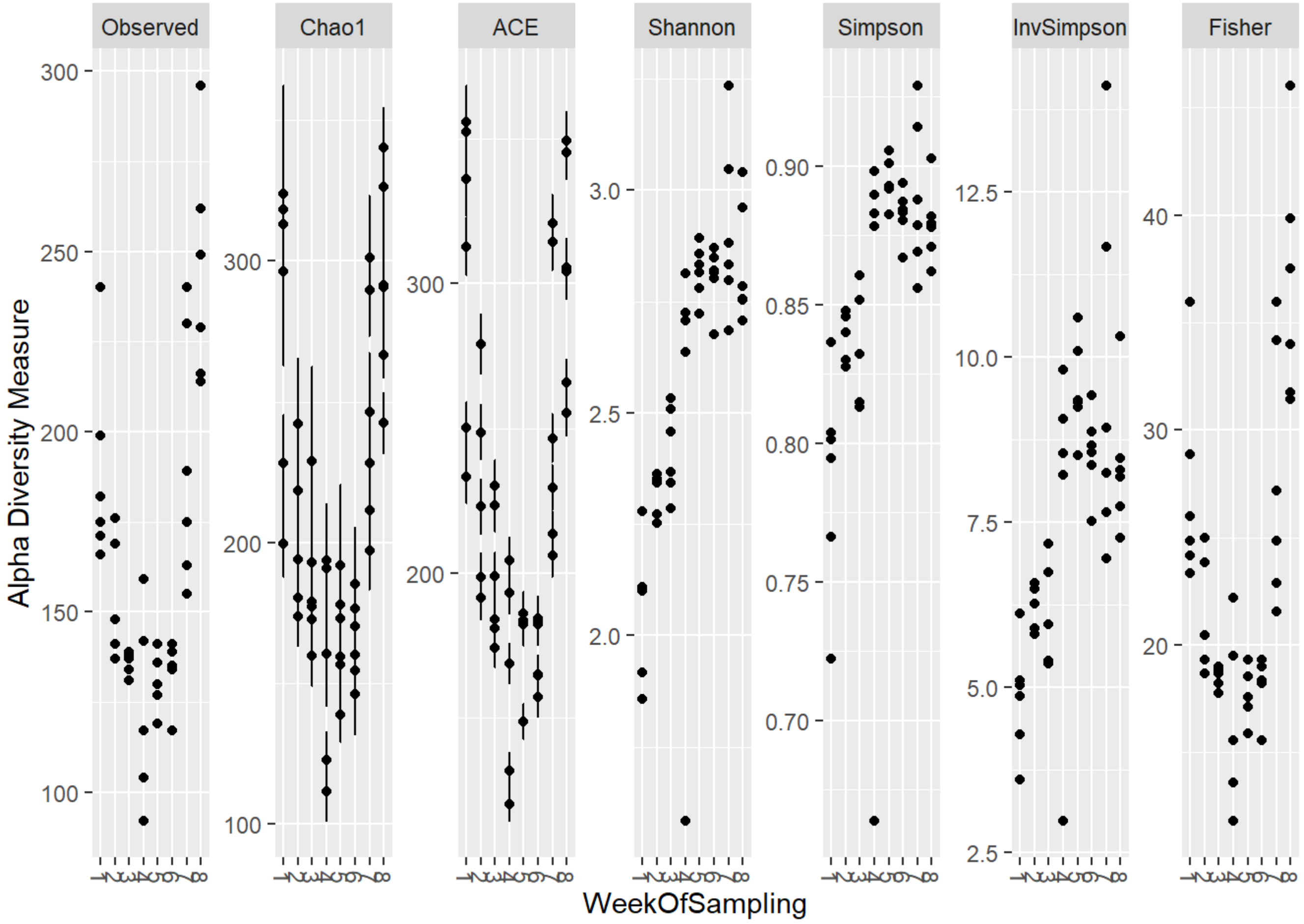
Observed
Chao1
ACE
Shannon
Simpson
InvSimpson
Fisher
Alpha Diversity Measure
WeekOfSampling

Observed

```
#observed OTUS biofilm
observed_biofilm <-
  boxplot_alpha(
    biofilm_pruned,
    x_var = "WeekOfSampling",
    index = "observed",
    fill.colors = c(
      "black",
      "black",
      "black",
      "black",
      "black",
      "black",
      "black",
      "black"
    ),
    outlier.fill = NA
  ) +
  labs(x = "Week of Sampling",
       y = "# observed zOTUs") +
  #theme(text = element_text(size = 18)) +
  theme_few()
# Remove the legend

# Apply custom colors to the plot and define text sizes
observed_biofilm <- observed_biofilm +
  theme(
    axis.text = element_text(size = 7, color = "black"),
    axis.title = element_text(size = 7, color = "black"),
    axis.ticks = element_line(color = "black", linewidth = 0.25),
    legend.text = element_text(size = 7),
    legend.title = element_blank(),
    # Remove the legend title for both legends
    legend.key.size = unit(4, "mm"),
    legend.position = "none"  # Remove the legend

  )
observed_biofilm
```

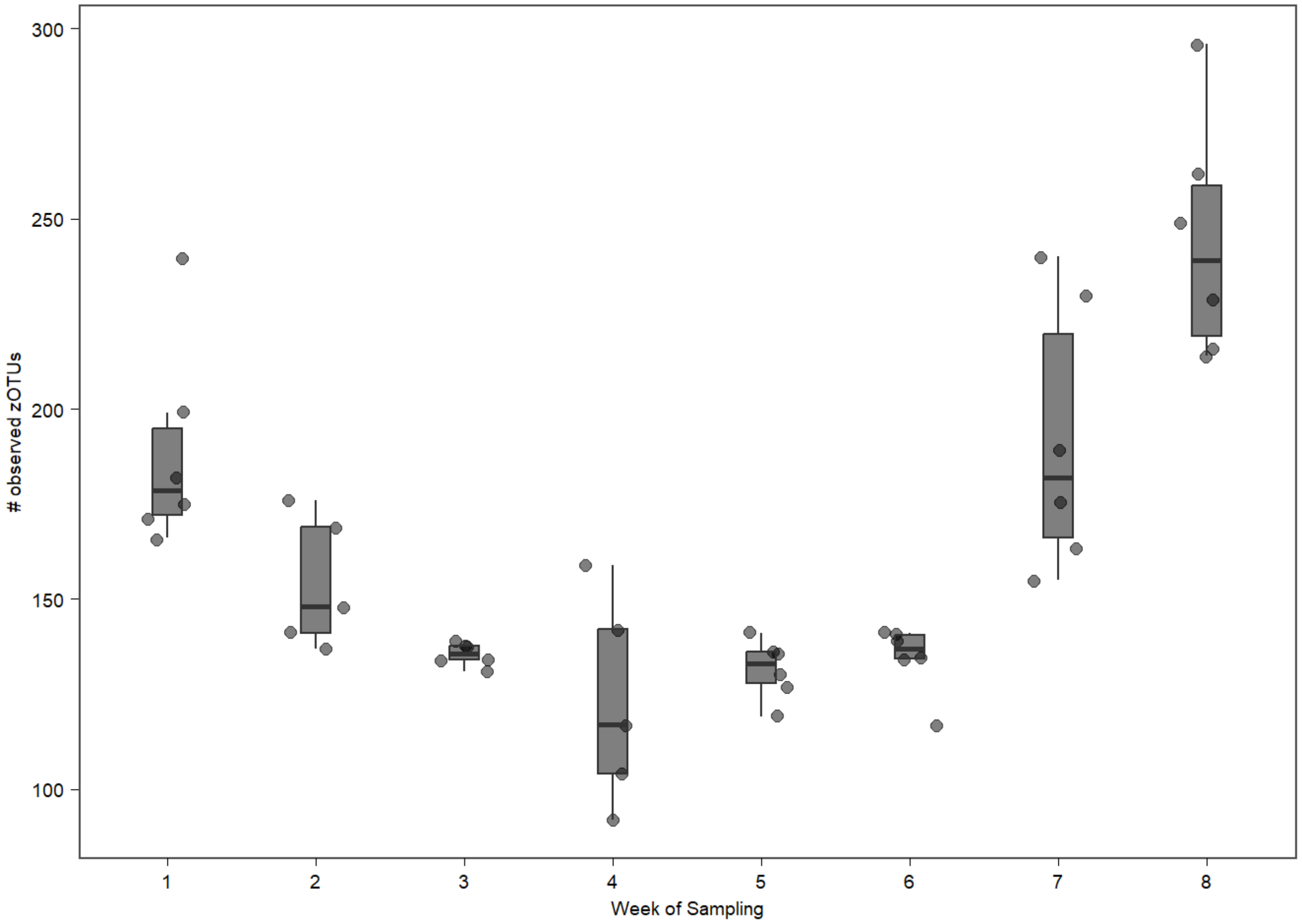


```
ggsave(
  filename = "observed_biofilm.pdf",
  dpi = 600,
  observed_biofilm,
  width = 88,
  height = 60,
  units = "mm"
)
```

InvSimpson

```
simpson_biofilm <-
  boxplot_alpha(
    biofilm_pruned,
    x_var = "WeekOfSampling",
    index = "inverse_simpson",
    fill.colors = c(
      "black",
      "black",
      "black",
      "black",
      "black",
      "black",
      "black",
      "black"
    ),
    outlier.fill = "white"
  ) +
  labs(x = "Week of Sampling",
       y = "Inverse Simpson") +
  #theme(text = element_text(size = 18)) +
  theme_few() # Remove the legend

# Apply custom colors to the plot and define text sizes
simpson_biofilm <- simpson_biofilm +
  theme(
    axis.text = element_text(size = 7, color = "black"),
    axis.title = element_text(size = 7, color = "black"),
    axis.ticks = element_line(color = "black", linewidth = 0.25),
    legend.text = element_text(size = 7),
    legend.title = element_blank(),
    # Remove the legend title for both legends
    legend.key.size = unit(4, "mm"),
    legend.position = "none"  # Remove the legend
  )
simpson_biofilm
```

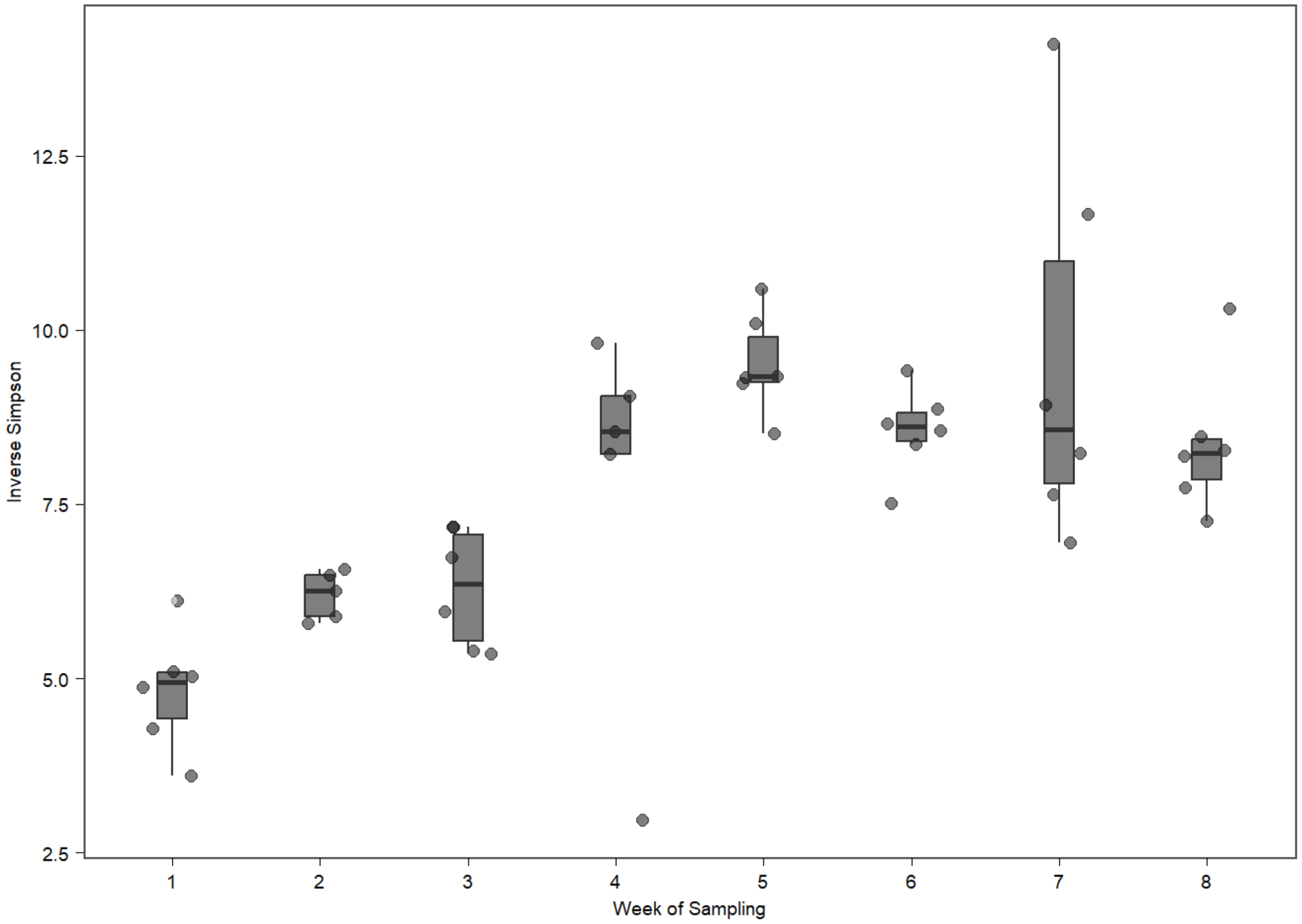


```
ggsave(
  filename = "simpson_biofilm.tiff",
  dpi = 600,
  simpson_biofilm,
  width = 90,
  height = 40,
  units = "mm"
)
```

Comparison Simpson vs observed (to make dure boxplot_alpha plots the same as plot_richness)

```
p = plot_richness(
  biofilm_pruned,
  "WeekOfSampling",
  color = NULL,
  shape = NULL,
  title = NULL,
  scales = "free_y",
  nrow = 1,
  shsi = NULL,
  measures = c("Observed", "Simpson"),
  sortby = NULL
) + geom_boxplot() + geom_jitter()
p
```

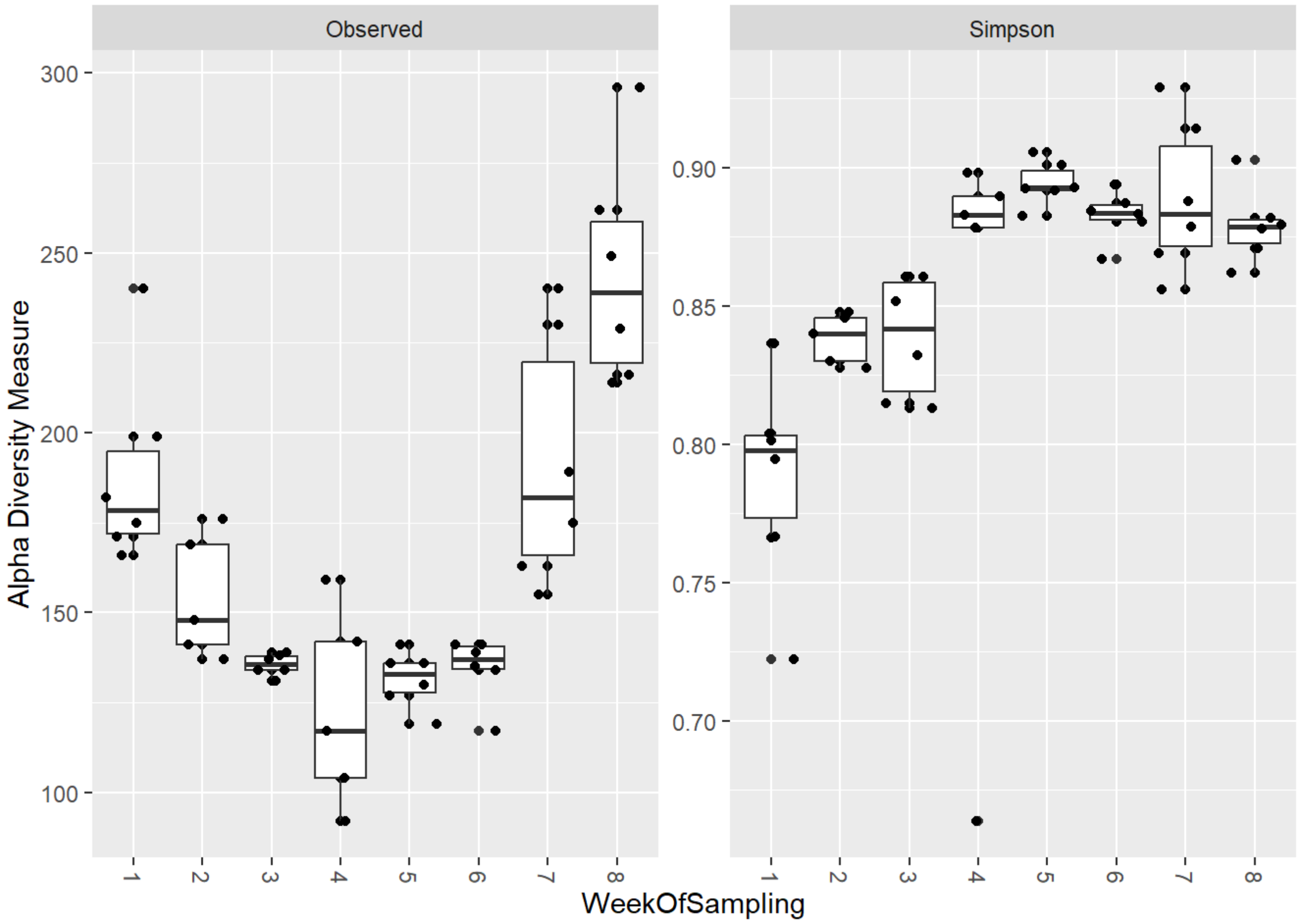

Water samples

```
water <- subset_samples(All_temporal_rarefied, SampleType == "water")
water_pruned <- prune_taxa(taxa_sums(water) > 0, water) #is this necessary?
```

Observed

```
observed_water <- plot_richness(water_pruned, x = "WeekOfSampling", measures=c("Observed"), t
itle = NULL) +
  labs(x = "Week of Sampling", y = "# observed zOTUs") +
  theme_classic() +
  theme(
    text = element_text(size = 7),
    legend.position = "none",
    panel.background = element_blank(),  # Remove panel background
    strip.background = element_blank(),  # Remove strip (facet label) background
    strip.text = element_blank()  # Remove strip (facet label) text
  )
observed_water
```

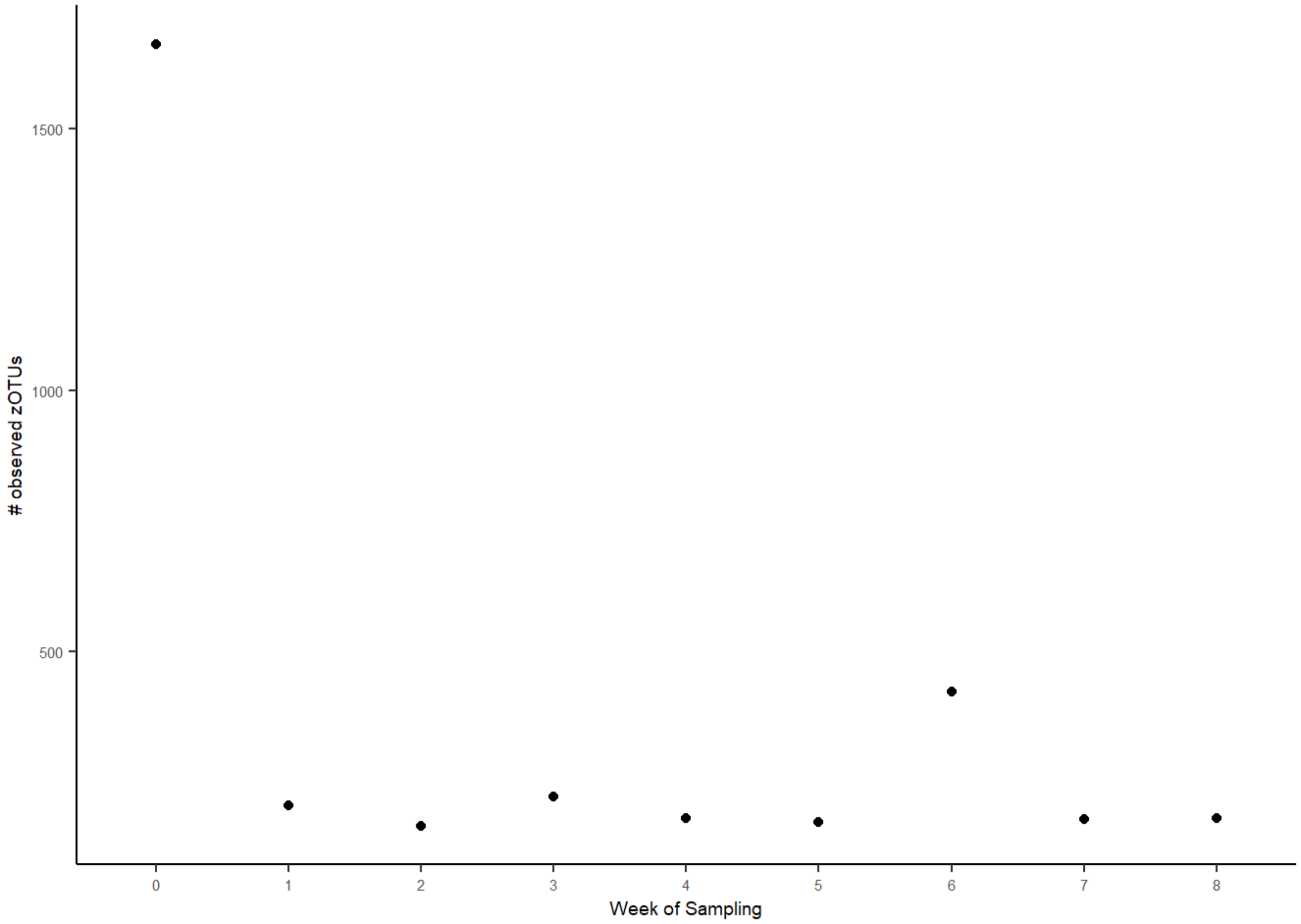


```
ggsave(filename = "observed_water.tiff", observed_water, dpi=600, width = 90, height = 60, un
its = "mm")
```

InvSimpson

```
isimpson_water <- plot_richness(water_pruned, x = "WeekOfSampling", measures=c("InvSimpson"),
title = NULL) +
  labs(x = "Week of Sampling", y = "Inverse Simpson") +
  theme_classic() +
  theme(
    text = element_text(size = 7),
    legend.position = "none",
    panel.background = element_blank(),  # Remove panel background
    strip.background = element_blank(),  # Remove strip (facet label) background
    strip.text = element_blank()  # Remove strip (facet label) text
  )
isimpson_water
```

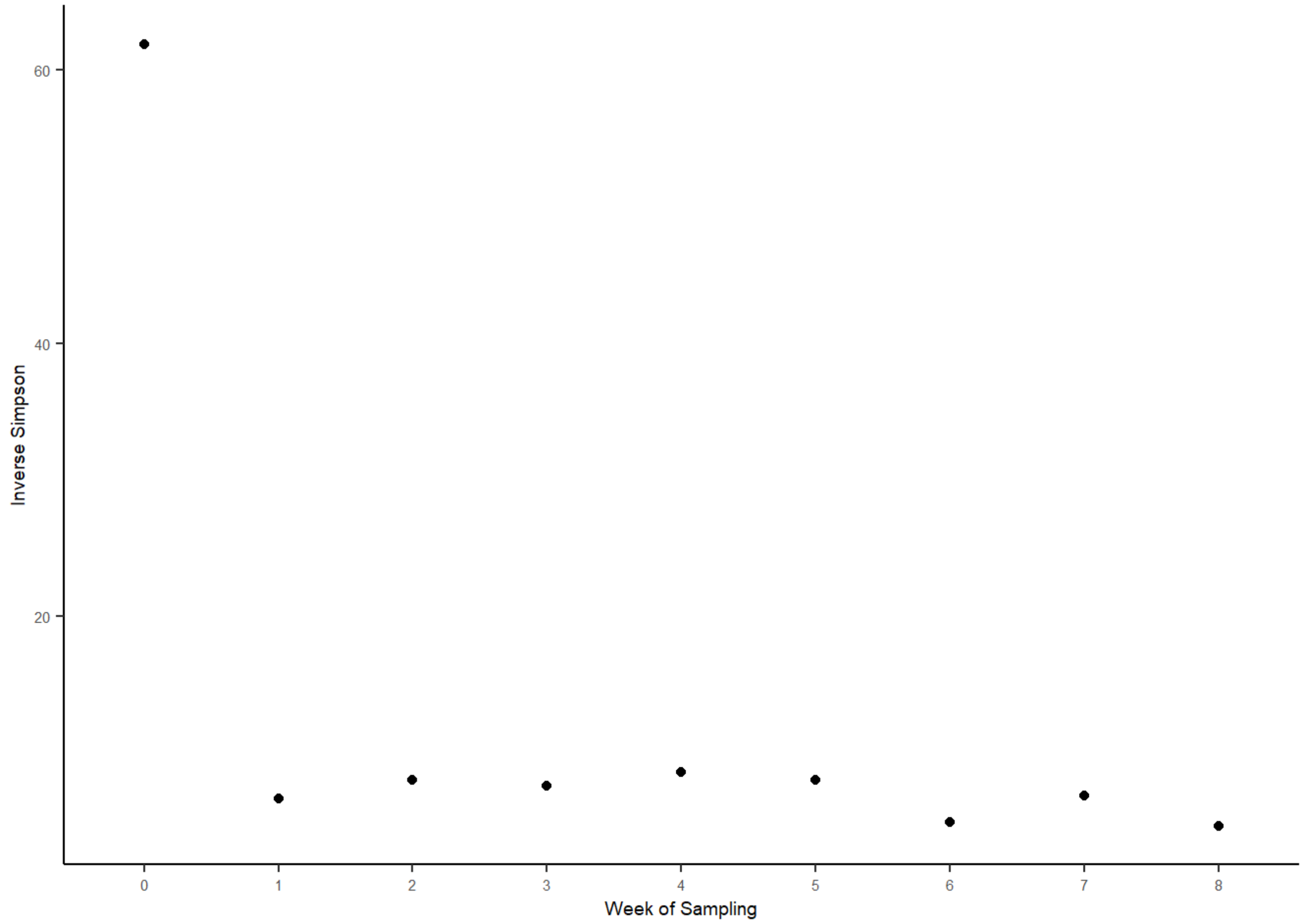


```
ggsave(filename = "isimpson_water.tiff", isimpson_water, dpi=600, width = 90, height = 60, un
its = "mm")
```

Boxplots diversity drop between water on week 0 and biofilms on week 1

Boxplot with number of zOTUs

```
WB1 <-
  subset_samples(
    All_temporal_rarefied,
    WeekOfSampling == "0" | (WeekOfSampling == "1" &
                               SampleType == "biofilm")
  )
WB1 <- prune_taxa(taxa_sums(WB1) > 0, WB1)
BW_richness <-
  as_tibble(estimate_richness(WB1), rownames = "Sample")
W_richness <- BW_richness %>% filter(Sample == "rw.1204")
B_richness <- BW_richness %>% filter(Sample != "rw.1204")

barplotBW <- ggplot() +
  geom_point(aes("Water \nWeek 0", Observed), data = W_richness) +
  geom_boxplot(aes("Biofilm \nWeek1", Observed), data = B_richness) +
  scale_x_discrete("", limits = c("Water \nWeek 0", "Biofilm \nWeek1")) +
  scale_y_continuous("Observed # of zOTUs") +
  theme_few() +
  theme(
    axis.text = element_text(size = 7, color = "black"),
    axis.title = element_text(size = 7, color = "black"),
    axis.ticks = element_line(color = "black", linewidth = 0.25)
  )

barplotBW
```

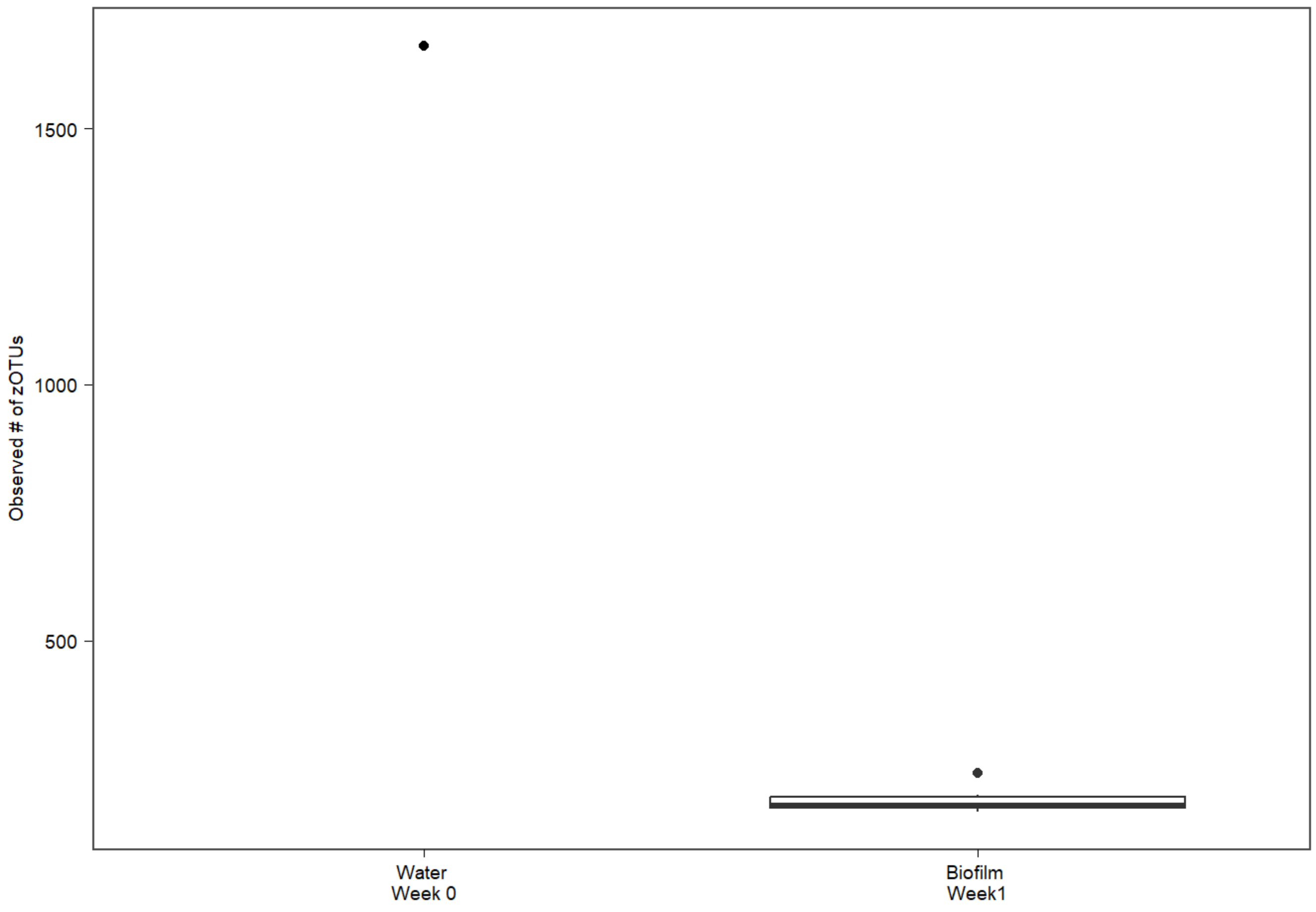

```
ggsave(
  filename = "barplotBW.pdf",
  dpi = 600,
  barplotBW,
  width = 38,
  height = 70,
  units = "mm"
)
```

Compositional evolution analysis at the ZOTU level

As we saw a temporal pattern with the NMDS plot, we went further and decided to look at the dynamics for a subset of the zOTUS of the dataset. To do this, we keep taxa that have a relative abundance > or = to 1% in at least one of the biofilm samples, the rest are merged. We then transform the relative abundance to absolute abundance using the median total cell count (flow cytometry) measured in each week of sampling. This allows us to have an evolutionary pattern over the 8 weeks of biofilm formation analysis. We then take the median of the absolute abundance for the 6 biofilm replicates we have each week. We use hierarchical clustering to cluster the evolution curves by similarity. We determine the optimal number of clusters: 13 clusters. For each zOTU, we normalise the absolute abundance between 0 and 1 (each zOTU has its absolute abundance normalised to itself).

We then plot each cluster in turn and classify the clusters into 4 different groups:

- initial: cluster containing the zOTUs present at high abundance (relative to themselves) at the beginning of the analysis.
- early: cluster containing the zOTUS present on the first week, but not at their highest abundance
- middle: clusters with zOTUs whose abundance start to increase after week2
- late: clusters with zOTUs whose abundance starts to increase after week 4

1. Subset temporal samples to contain all biofilms and the water phase on week 0 and subset biofilms only.

```
BtemporalW <-
  subset_samples(All_temporal,
                 SampleType == "biofilm" | DateOfSampling == "12.04.2021")
Btemporal <- subset_samples(All_temporal, SampleType == "biofilm")
```

Get total read counts for samples

```
dat_tot <- data.table::data.table(as(sample_data(Btemporal), "data.frame"), TotalReads = samp
le_sums(Btemporal), keep.rownames = TRUE)
```

2. Only keep taxa with RA > or = to 1%

```
Btemporal_relabund <- transform(Btemporal, "compositional")
superior <- function(x){
  x >= 0.01
}

f_superior=filterfun_sample(superior)
whsuperior=genefilter_sample(Btemporal_relabund ,f_superior,A=1)
Name= names(whsuperior[!whsuperior])
merged=merge_taxa(Btemporal_relabund, Name, archetype=1)
ntaxa(merged)
```

```
## [1] 48
```

```
ntaxa(Btemporal_relabund)
```

```
## [1] 4090
```

We can see that we go from 4129 taxa to 48 taxa when applying the 1% filter.

```
#GET LIST OF ZOTUS

#FILTER dat_tot BASED ON LIST OF ZOTUS
#z=prune_taxa(whsuperior,Btemporal_relabund )
#dat_fil <- data.table(as(sample_data(z), "data.frame"), KeptReads = sample_sums(z), keep.row
names = TRUE)

#dat_tot <- dat_tot %>% select(X.SampleID, TotalReads)
#dat_fil <- dat_fil %>% select(X.SampleID, KeptReads)

#dat <- dat_tot %>% left_join(dat_fil) %>% mutate(PercKept = KeptReads/TotalReads*100)
#> min(dat$KeptReads)
#[1] 0.9075711
#> max(dat$KeptReads)
#[1] 0.9848684
```

3. convert to dataframe and create a new column for the absolute abundance

Absolute abundance is obtained by multiplying the relative abundance with the median total cell count

```
df_Btemporal <- psmelt(merged)
df_Btemporal$AA <- df_Btemporal$Abundance * df_Btemporal$SG *1000
```

Plot of the evolution

```
ggplot(df_Btemporal, (aes(x=WeekOfSampling, y=AA, group=WeekOfSampling)))+
  geom_boxplot()+
  facet_wrap(~OTU, scales = "free_y")
```

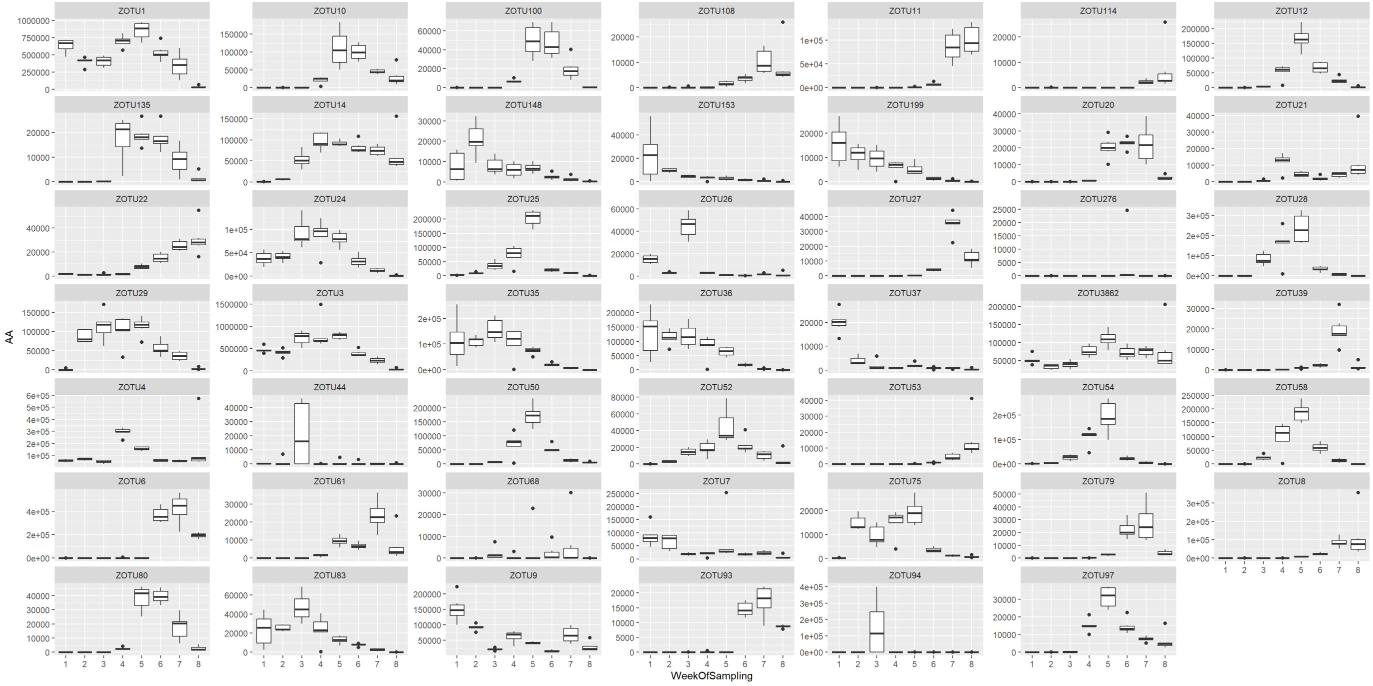

4. Take the median abundance for each samples

```
library(dplyr)

# Group by 'OTU' and 'WeekOfSampling', calculate the median of 'AA' for each group
grouped_df <- df_Btemporal %>%
  group_by(OTU, WeekOfSampling) %>%
  summarise(medianAA = median(AA))
```

```
## `summarise()` has grouped output by 'OTU'. You can override using the `.groups`
## argument.
```

```
# Display the resulting dataframe
print(grouped_df)
```

```
## # A tibble: 384 × 3
## # Groups:   OTU [48]
##    OTU    WeekOfSampling medianAA
##    <chr>  <fct>             <dbl>
##  1 ZOTU1  1              671960.
##  2 ZOTU1  2              419972.
##  3 ZOTU1  3              419980.
##  4 ZOTU1  4              707215.
##  5 ZOTU1  5              882556.
##  6 ZOTU1  6              507614.
##  7 ZOTU1  7              355791.
##  8 ZOTU1  8               32045.
##  9 ZOTU10 1                  61.3
## 10 ZOTU10 2                 153.
## # ℹ 374 more rows
```

5. calculate distance with correlation method.

```
#install.packages("TSclust")
library(TSclust)
```

```
## Lade nötiges Paket: pdc
```

```
## Lade nötiges Paket: cluster
```

```
## Registered S3 method overwritten by 'quantmod':
##   method            from
##   as.zoo.data.frame zoo
```

```
library(tidyr)

df <- grouped_df

pivot_df <- pivot_wider(data = df, names_from = OTU, values_from = medianAA) # Pivot the data
frame to wide format

# Rename the 'WeekOfSampling' column to 'Week'
colnames(pivot_df)[colnames(pivot_df) == "WeekOfSampling"] <- "Week"

# Print the pivoted dataframe
print(pivot_df)
```

```
## # A tibble: 8 × 49
##   Week    ZOTU1   ZOTU10 ZOTU100 ZOTU108  ZOTU11 ZOTU114  ZOTU12 ZOTU135 ZOTU14
##   <fct>   <dbl>    <dbl>   <dbl>   <dbl>   <dbl>   <dbl>   <dbl>   <dbl>  <dbl>
## 1 1     671960.     61.3    39.9    45.0   291.        0     175.    29.1   338.
## 2 2     419972.    153.     35.2    42.3    90.4       0     339.     0    6714.
## 3 3     419980.    641.     43.8    43.8   199.        0    4509.   228.  51262.
## 4 4     707215.  25597.   6425.     77.6   578.        0   61135. 21222.  89853.
## 5 5     882556. 105009.  49084.   1431.   1127.        0  163655. 18091.  89752.
## 6 6     507614.  98309.  43008.   4069.   6295.        0   66071. 16538.  75404.
## 7 7     355791.  43749.  17425.   8744.  85435.    2167.  22162.  9245.  73848.
## 8 8      32045.  20699.    664.   5261.  94247.    2742.   1355.   615.  47936.
## # ℹ 39 more variables: ZOTU148 <dbl>, ZOTU153 <dbl>, ZOTU199 <dbl>,
## #   ZOTU20 <dbl>, ZOTU21 <dbl>, ZOTU22 <dbl>, ZOTU24 <dbl>, ZOTU25 <dbl>,
## #   ZOTU26 <dbl>, ZOTU27 <dbl>, ZOTU276 <dbl>, ZOTU28 <dbl>, ZOTU29 <dbl>,
## #   ZOTU3 <dbl>, ZOTU35 <dbl>, ZOTU36 <dbl>, ZOTU37 <dbl>, ZOTU3862 <dbl>,
## #   ZOTU39 <dbl>, ZOTU4 <dbl>, ZOTU44 <dbl>, ZOTU50 <dbl>, ZOTU52 <dbl>,
## #   ZOTU53 <dbl>, ZOTU54 <dbl>, ZOTU58 <dbl>, ZOTU6 <dbl>, ZOTU61 <dbl>,
## #   ZOTU68 <dbl>, ZOTU7 <dbl>, ZOTU75 <dbl>, ZOTU79 <dbl>, ZOTU8 <dbl>, …
```

```
pivot_df <- pivot_df[, -which(names(pivot_df) == "Week")]
pivot_df <- pivot_df[, -which(names(pivot_df) == "ZOTU3862")] #these are the merged taxa unde
r "Name"
# Print the modified dataframe
print(pivot_df)
```

```
## # A tibble: 8 × 47
##     ZOTU1   ZOTU10 ZOTU100 ZOTU108  ZOTU11 ZOTU114 ZOTU12 ZOTU135 ZOTU14 ZOTU148
##     <dbl>    <dbl>   <dbl>   <dbl>   <dbl>   <dbl>  <dbl>   <dbl>  <dbl>   <dbl>
## 1 671960.     61.3    39.9    45.0   291.        0 1.75e2    29.1   338.   6311.
## 2 419972.    153.     35.2    42.3    90.4       0 3.39e2     0    6714.  19641.
## 3 419980.    641.     43.8    43.8   199.        0 4.51e3   228.  51262.   6210.
## 4 707215.  25597.   6425.     77.6   578.        0 6.11e4 21222.  89853.   5972.
## 5 882556. 105009.  49084.   1431.   1127.        0 1.64e5 18091.  89752.   6535.
## 6 507614.  98309.  43008.   4069.   6295.        0 6.61e4 16538.  75404.   2407.
## 7 355791.  43749.  17425.   8744.  85435.    2167. 2.22e4  9245.  73848.   1021.
## 8  32045.  20699.    664.   5261.  94247.    2742. 1.35e3   615.  47936.    193.
## # ℹ 37 more variables: ZOTU153 <dbl>, ZOTU199 <dbl>, ZOTU20 <dbl>,
## #   ZOTU21 <dbl>, ZOTU22 <dbl>, ZOTU24 <dbl>, ZOTU25 <dbl>, ZOTU26 <dbl>,
## #   ZOTU27 <dbl>, ZOTU276 <dbl>, ZOTU28 <dbl>, ZOTU29 <dbl>, ZOTU3 <dbl>,
## #   ZOTU35 <dbl>, ZOTU36 <dbl>, ZOTU37 <dbl>, ZOTU39 <dbl>, ZOTU4 <dbl>,
## #   ZOTU44 <dbl>, ZOTU50 <dbl>, ZOTU52 <dbl>, ZOTU53 <dbl>, ZOTU54 <dbl>,
## #   ZOTU58 <dbl>, ZOTU6 <dbl>, ZOTU61 <dbl>, ZOTU68 <dbl>, ZOTU7 <dbl>,
## #   ZOTU75 <dbl>, ZOTU79 <dbl>, ZOTU8 <dbl>, ZOTU80 <dbl>, ZOTU83 <dbl>, …
```

```
D1 <- diss(pivot_df, "COR")
summary(D1)
```

```
## Class: dist
## Distance matrix by lower triangle : d21, d22, ..., d2n, d32, ...
## Size: 47
## Labels: ZOTU1 ZOTU10 ZOTU100 ZOTU108 ZOTU11 ZOTU114 ZOTU12 ZOTU135 ZOTU14 ZOTU148 ZOTU153
ZOTU199 ZOTU20 ZOTU21 ZOTU22 ZOTU24 ZOTU25 ZOTU26 ZOTU27 ZOTU276 ZOTU28 ZOTU29 ZOTU3 ZOTU35 Z
OTU36 ZOTU37 ZOTU39 ZOTU4 ZOTU44 ZOTU50 ZOTU52 ZOTU53 ZOTU54 ZOTU58 ZOTU6 ZOTU61 ZOTU68 ZOTU7
ZOTU75 ZOTU79 ZOTU8 ZOTU80 ZOTU83 ZOTU9 ZOTU93 ZOTU94 ZOTU97
## call: as.dist.default(m = (distances))
## method:
## Euclidean matrix (Gower 1966): TRUE
```

6. Plot the dendrogram

```
C1 <- hclust(D1, method = )
plot(C1)
```

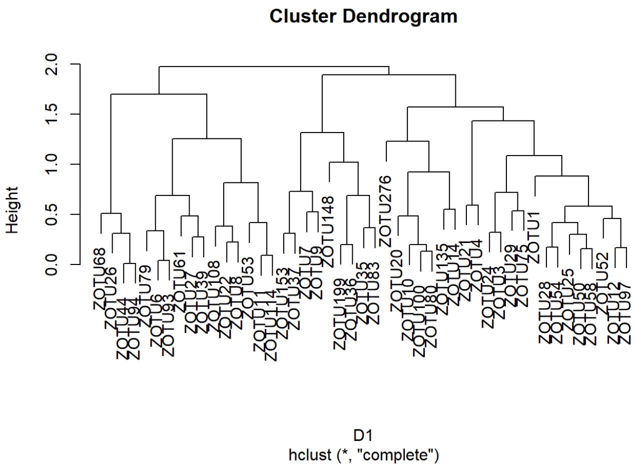

6b Select optimal number of clusters

```
library(NbClust)

clust_opt <- NbClust(diss = D1, distance = NULL, method = "complete", index = "silhouette")
```

```
##
##  Only frey, mcclain, cindex, sihouette and dunn can be computed. To compute the other indi
ces, data matrix is needed
```

```
print(clust_opt$Best.nc)
```

```
## Number_clusters     Value_Index
##         13.0000          0.4635
```

```
plot(seq(2,15),clust_opt$All.index)
```

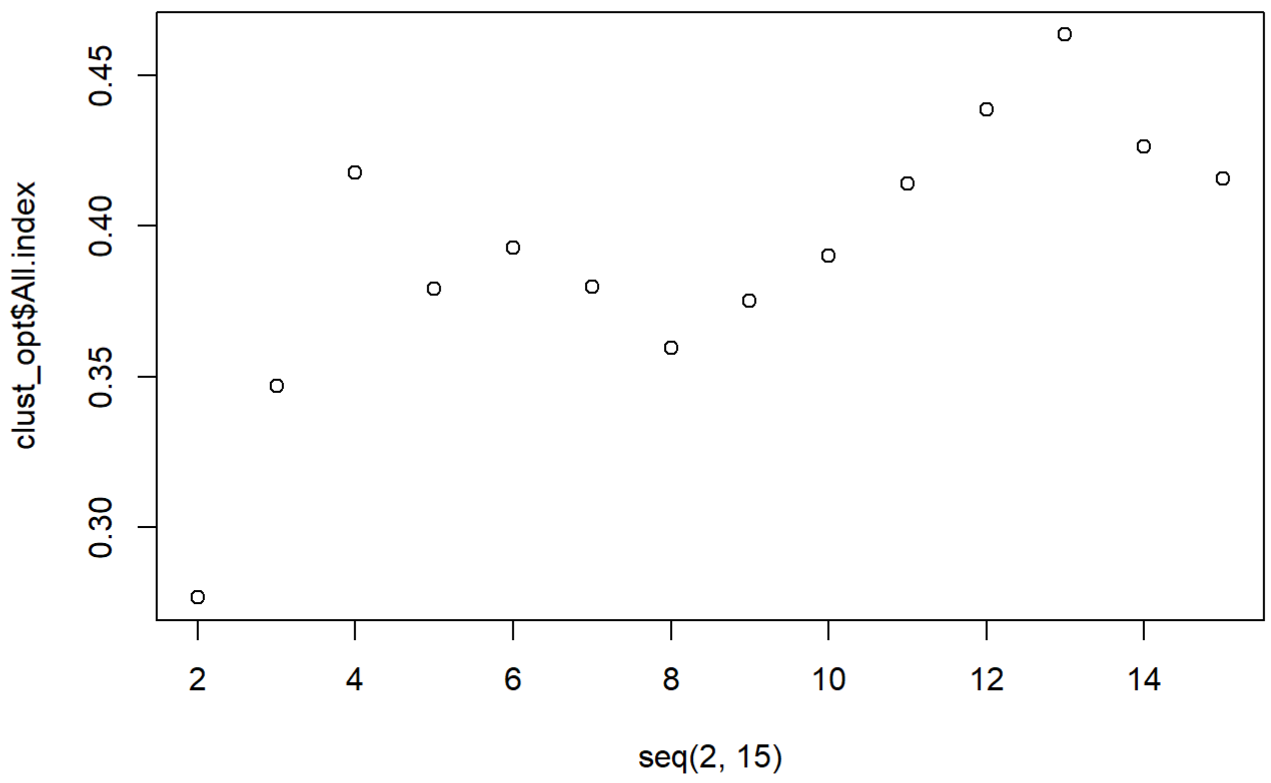

13 is the optimal number of clusters

7. seT number of clusters

```
cut_avg <- cutree(C1, k = 13) # Use value provided by NbClust
```

8. remove the merged taxa below 1%

```
filtered_df <- df %>%
  filter(OTU != "ZOTU3862")
```

9. Add cluster number to the data

```
# Specific values for each OTU
specific_values <- cut_avg

# Create a named vector for the mapping
otu_specific_mapping <-
  setNames(specific_values, unique(filtered_df$OTU))

# Add SpecificValue to the original dataframe
filtered_df$SpecificValue <- otu_specific_mapping[filtered_df$OTU]

print(filtered_df)
```

```
## # A tibble: 376 × 4
## # Groups:   OTU [47]
##    OTU    WeekOfSampling medianAA SpecificValue
##    <chr>  <fct>             <dbl>         <int>
##  1 ZOTU1  1              671960.              1
##  2 ZOTU1  2              419972.              1
##  3 ZOTU1  3              419980.              1
##  4 ZOTU1  4              707215.              1
##  5 ZOTU1  5              882556.              1
##  6 ZOTU1  6              507614.              1
##  7 ZOTU1  7              355791.              1
##  8 ZOTU1  8               32045.              1
##  9 ZOTU10 1                  61.3             2
## 10 ZOTU10 2                 153.              2
## # ℹ 366 more rows
```

10. Normalize abundances

```
# Normalize values between 0 and 1
normalized_df <- filtered_df%>%
  mutate(
    Normalized_AA = (medianAA - min(medianAA)) / (max(medianAA) - min(medianAA))
  )

print(normalized_df)
```

```
## # A tibble: 376 × 5
## # Groups:   OTU [47]
##    OTU    WeekOfSampling medianAA SpecificValue Normalized_AA
##    <chr>  <fct>             <dbl>         <int>         <dbl>
##  1 ZOTU1  1              671960.              1      0.752
##  2 ZOTU1  2              419972.              1      0.456
##  3 ZOTU1  3              419980.              1      0.456
##  4 ZOTU1  4              707215.              1      0.794
##  5 ZOTU1  5              882556.              1      1
##  6 ZOTU1  6              507614.              1      0.559
##  7 ZOTU1  7              355791.              1      0.381
##  8 ZOTU1  8               32045.              1      0
##  9 ZOTU10 1                  61.3             2      0
## 10 ZOTU10 2                 153.              2      0.000871
## # ℹ 366 more rows
```

11. Plot by clusters

```
taxInfo <- df_Btemporal %>% select(OTU, Phylum, Class, Order, Family, Genus, Species) %>% dis
tinct()
normalized_df <- normalized_df %>% left_join(taxInfo, by = "OTU")
```

All cluster in one graph

```
ggplot(normalized_df, (aes(x=WeekOfSampling, y=Normalized_AA, group=OTU, color=OTU)))+
  geom_line()+
  facet_wrap(~SpecificValue, scales = "free_y")
```

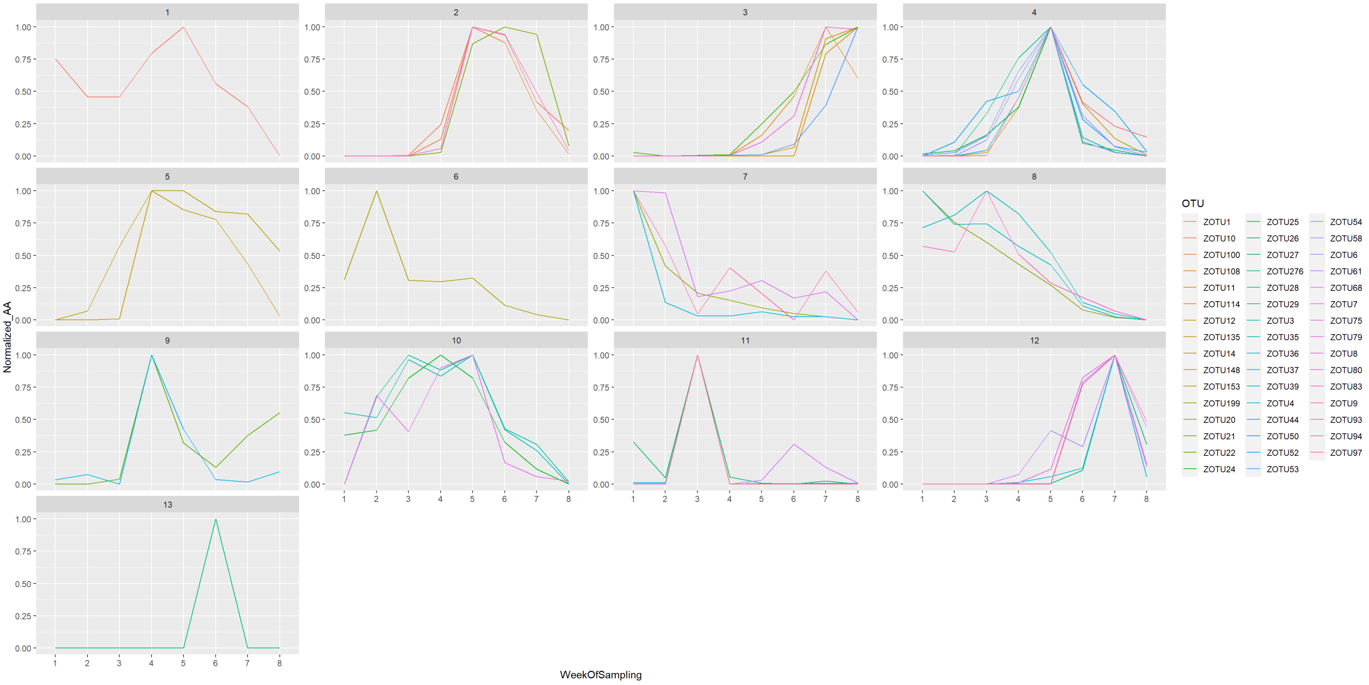


```
normalized_df %>% group_by(SpecificValue) %>% summarise(n_OTU = length(unique(OTU)))
```

SpecificValue	n_OTU
<int>	<int>
1	1

SpecificValue <int>	n_OTU <int>
2	4
3	6
4	8
5	2
6	1
7	4
8	4
9	2
10	4

1-10 of 13 rows Previous **1** 2 Next

Plot 4 graphs with the clusters according to subjective temporal dynamics (ie. initial, early, middle, later)

```
summary_data <- normalized_df %>%
  group_by(SpecificValue, WeekOfSampling) %>%
  summarize(median_abundance = median(Normalized_AA))
```

```
## `summarise()` has grouped output by 'SpecificValue'. You can override using the
## `.groups` argument.
```

```
initial=subset(summary_data , SpecificValue=="7" | SpecificValue=="8" |SpecificValue=="6"  )
early=subset(summary_data ,  SpecificValue=="1" | SpecificValue=="10" )
middle=subset(summary_data , SpecificValue=="11" | SpecificValue=="4" |SpecificValue=="5"|Spe
cificValue=="9")
late=subset(summary_data , SpecificValue=="2" | SpecificValue=="12" |SpecificValue=="13"|Spec
ificValue=="3")

custom.col <- c("#8D2B36", "#C4AA3F", "#658782","#24604D")

p_initial= ggplot(initial, aes(x = WeekOfSampling, y = median_abundance, group = SpecificValu
e, color=as.factor(SpecificValue))) +
  geom_line() +
  scale_color_manual(values = custom.col)+
  labs(x = "Time (Weeks)", y = "Median Normalized Abundance", color = "SpecificValue") +
  theme_few()+
  theme(
    axis.text = element_text(size = 7, color = "black"),
    axis.title = element_text(size = 7, color = "black"),
    axis.ticks = element_line(color = "black", linewidth = 0.25),
    legend.position = "none"  # Remove the legend
  )

p_initial
```

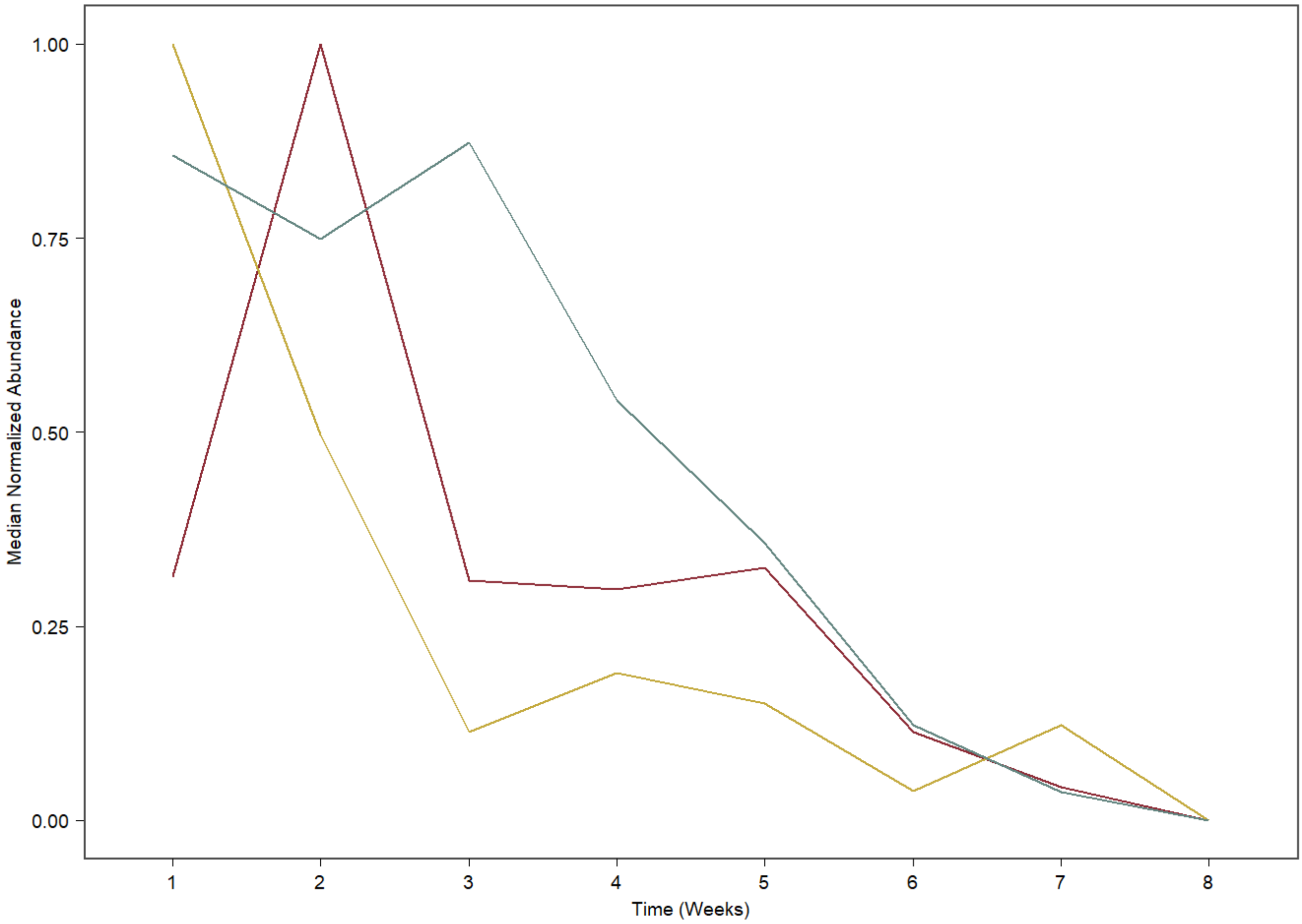


```
ggsave(
  filename = "p_initial.pdf",
  dpi = 600,
  plot = p_initial,
  width = 55,
  height = 55,
  units = "mm"
)

p_early= ggplot(early, aes(x = WeekOfSampling, y = median_abundance, group = SpecificValue, c
olor=as.factor(SpecificValue))) +
  geom_line() +
  scale_color_manual(values = custom.col)+
  labs(x = "Time (Weeks)", y = "Median Normalized Abundance", color = "SpecificValue") +
  theme_few()+
  theme(
    axis.text = element_text(size = 7, color = "black"),
    axis.title = element_text(size = 7, color = "black"),
    axis.ticks = element_line(color = "black", linewidth = 0.25),
    legend.position = "none"  # Remove the legend
  )
p_early
```

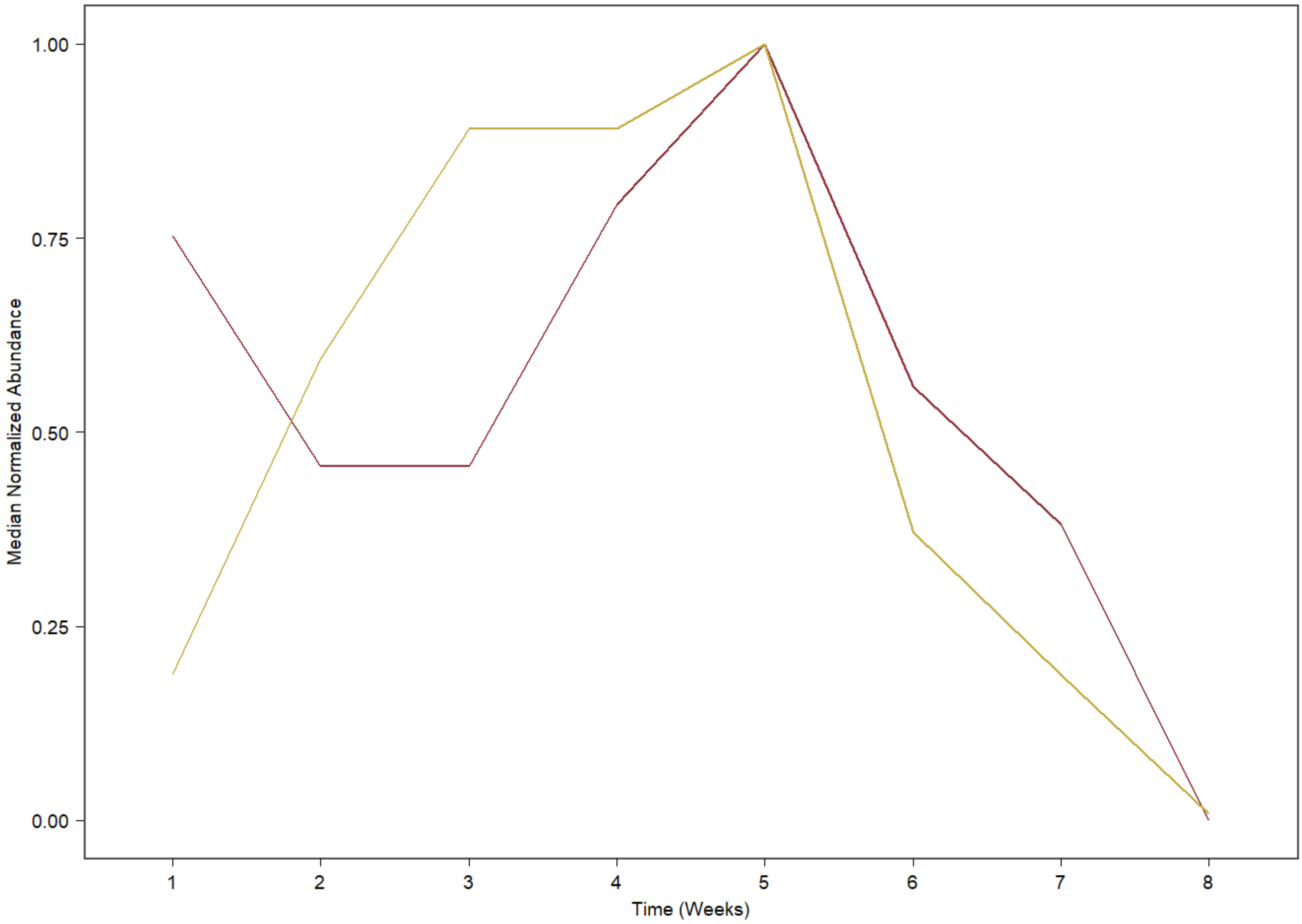


```
ggsave(
  filename = "p_early.pdf",
  dpi = 600,
  plot = p_early,
  width = 55,
  height = 55,
  units = "mm"
)

p_middle= ggplot(middle, aes(x = WeekOfSampling, y = median_abundance, group = SpecificValue,
color=as.factor(SpecificValue))) +
  geom_line() +
  scale_color_manual(values = custom.col)+
  labs(x = "Time (Weeks)", y = "Median Normalized Abundance", color = "SpecificValue") +
  theme_few()+
  theme(
    axis.text = element_text(size = 7, color = "black"),
    axis.title = element_text(size = 7, color = "black"),
    axis.ticks = element_line(color = "black", linewidth = 0.25),
    legend.position = "none"  # Remove the legend
  )
p_middle
```

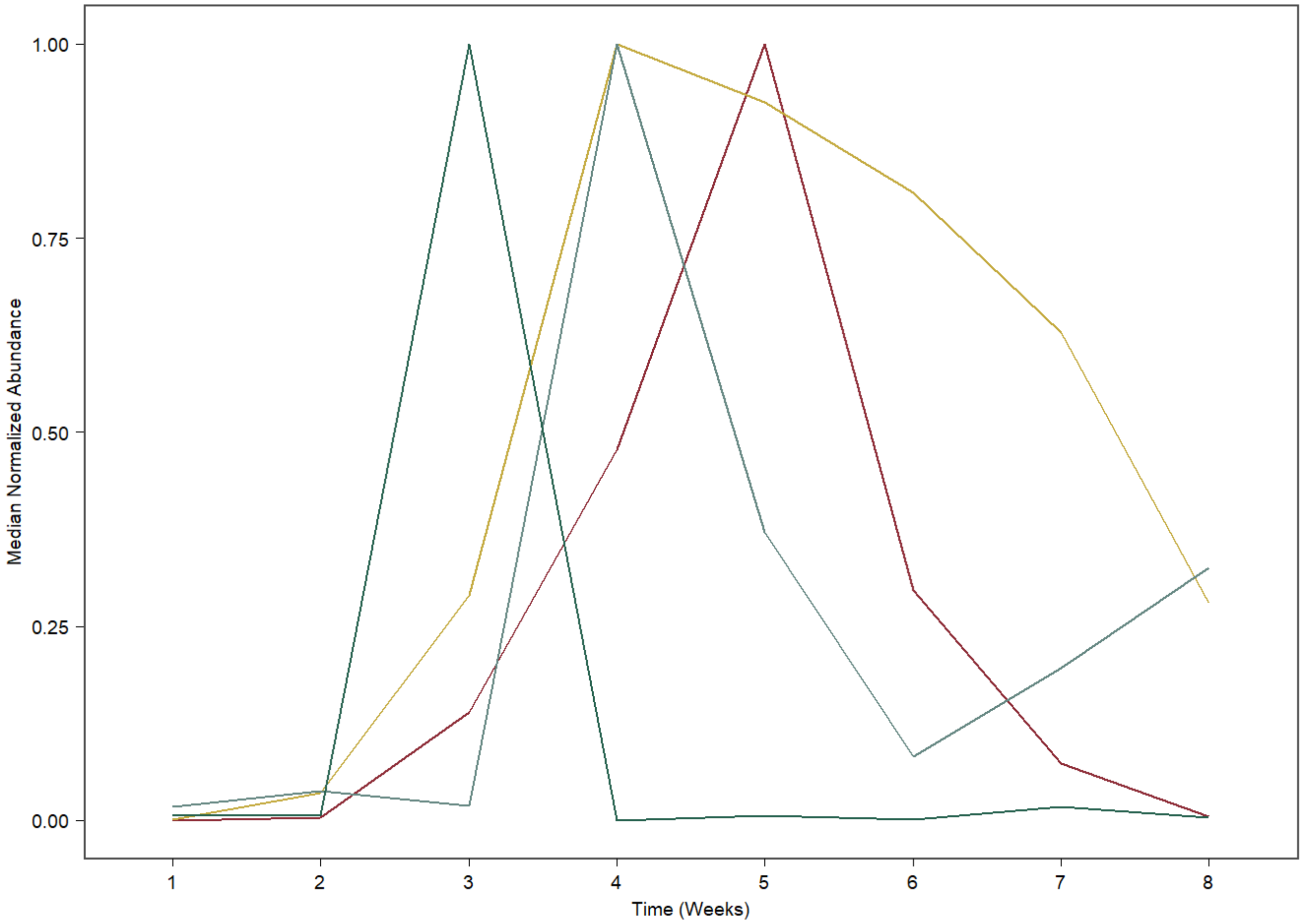


```
ggsave(
  filename = "p_middle.pdf",
  dpi = 600,
  plot = p_middle,
  width = 55,
  height = 55,
  units = "mm"
)

p_late= ggplot(late, aes(x = WeekOfSampling, y = median_abundance, group = SpecificValue, col
or=as.factor(SpecificValue))) +
  geom_line() +
  scale_color_manual(values = custom.col)+
  labs(x = "Time (Weeks)", y = "Median Normalized Abundance", color = "SpecificValue") +
  theme_few()+
  theme(
    axis.text = element_text(size = 7, color = "black"),
    axis.title = element_text(size = 7, color = "black"),
    axis.ticks = element_line(color = "black", linewidth = 0.25),
    legend.position = "none"  # Remove the Legend
  )
p_late
```

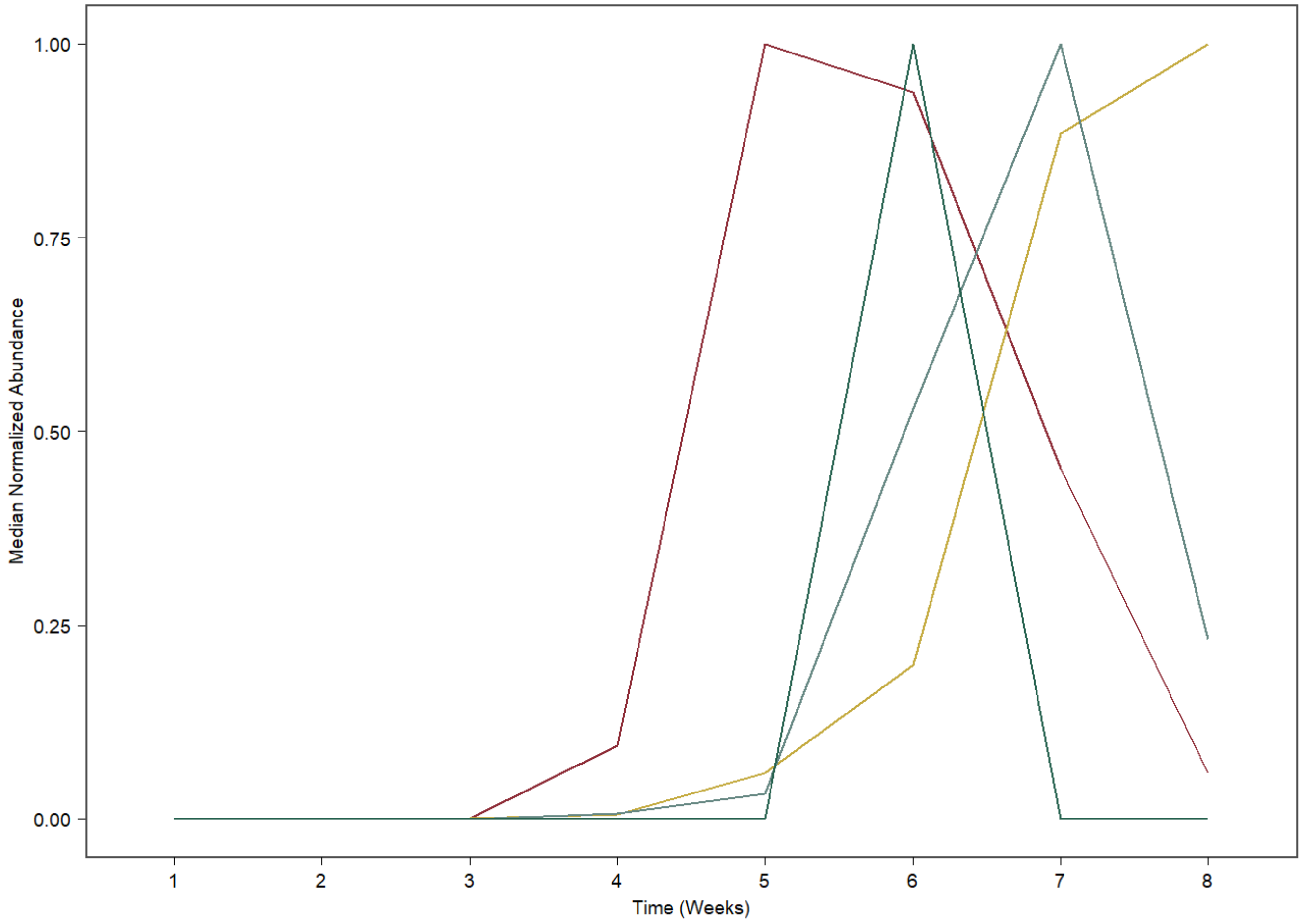


```
ggsave(
  filename = "p_late.pdf",
  dpi = 600,
  plot = p_late,
  width = 55,
  height = 55,
  units = "mm"
)
```

Graph below shows that the three different Legionella spp. have different dynamics during the biofilm formation process.

```
legio <- subset(normalized_df, Genus=="Legionella")

p_legio <- ggplot(legio, (aes(x=WeekOfSampling, y=Normalized_AA, group=OTU, color=Species)))+
  geom_line()+
  scale_color_manual(values = custom.col)+
  labs(x = "Time (Weeks)", y = "Normalized absolute abundance", color = "Species") +
  theme_few()+
  theme(
    axis.text = element_text(size = 7, color = "black"),
    axis.title = element_text(size = 7, color = "black"),
    axis.ticks = element_line(color = "black", linewidth = 0.25)
  )
p_legio
```

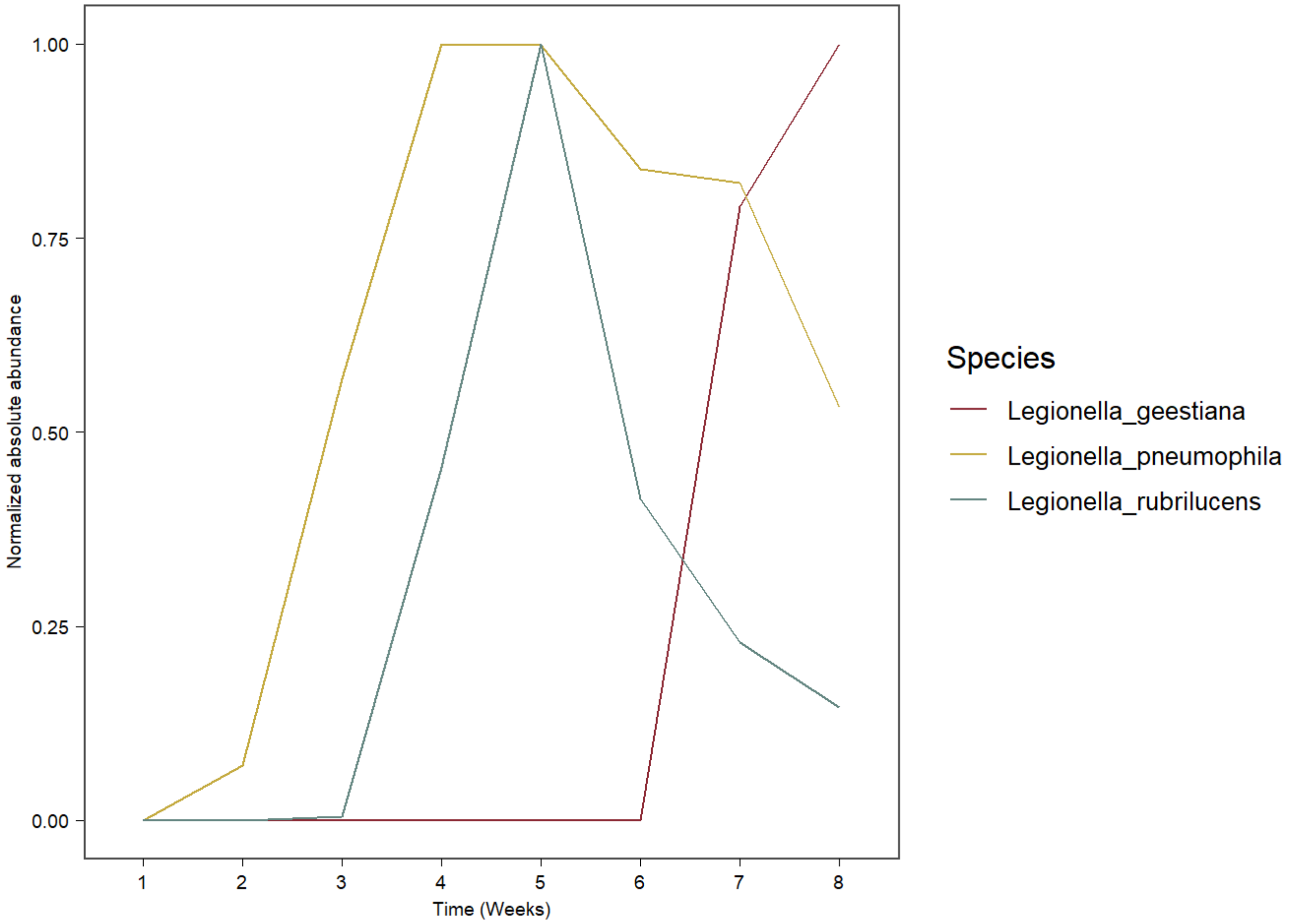


```
ggsave(
  filename = "p_legio.tiff",
  dpi = 600,
  plot = p_legio,
  width = 120,
  height = 60,
  units = "mm"
)
```

plot individual cluster (for supplements)

In the graphs below, we plot the evolution patterns of zOTUs by cluster. This will be part of the supplementary material of the paper.

```
tax_levels <- c("P_", "C_", "O_", "F_", "G_", "S_")

normalized_df <- normalized_df %>% ungroup() %>%
  mutate(Phylum = ifelse(is.na(Phylum), "Unassigned", Phylum),
         Species = ifelse(stringr::str_detect(Species, "uncultured"), NA, Species),
         Genus = ifelse(stringr::str_detect(Genus, "uncultured"), NA, Genus),
         tax = paste(Phylum, Class, Order, Family, Genus, Species),
         NA_nums = stringr::str_count(tax, pattern = "NA"),
         label = stringr::word(tax, 6 - NA_nums, sep = " "),
         label = gsub("_", " ", label),
         label = paste0(tax_levels[6 - NA_nums], label))
```

```
custom.col <- c("#8D2B36", "#CF897A",  "#C4AA3F", "#715F4B",  "#658782", "#3f5c6a", "#24604
D")

subset_cluster1 <- subset(normalized_df, SpecificValue == "1")

C1=ggplot(subset_cluster1, aes(x = WeekOfSampling, y = Normalized_AA,  color = label, legend=
label, group = OTU)) +
  geom_line(size=1) +
  scale_color_manual("Taxonomic information", values = custom.col) +  # Use custom colors for
lines
  xlab(bquote(Time~(Weeks)))+
  ylab(bquote(Normalized~absolute~abundance))+
  theme_classic()+
  ggtitle("Cluster 1")+
  theme(
  title = element_text(size = 7, color="black"),
  axis.text = element_text(size = 7, color="black"),
  axis.title = element_text(size = 7, color="black"),
  axis.ticks= element_line(color = "black", linewidth=0.25),
  legend.text = element_text(size = 6),
  legend.title = element_text(size = 6),
  legend.key.size = unit(3, "mm"),
  legend.position = c(0.20, 0.90)
)
```

```
## Warning: Using `size` aesthetic for lines was deprecated in ggplot2 3.4.0.
## ℹ Please use `linewidth` instead.
## This warning is displayed once every 8 hours.
## Call `lifecycle::last_lifecycle_warnings()` to see where this warning was
## generated.
```

```
ggsave(filename = "C1.tiff", dpi=600, C1, width = 90, height = 60, units = "mm")
```

```
subset_cluster2 <- subset(normalized_df, SpecificValue == "2")

C2=ggplot(subset_cluster2, aes(x = WeekOfSampling, y = Normalized_AA,  color = label, legend=
label, group = OTU)) +
  geom_line(size=1) +
  scale_color_manual("Taxonomic information", values = custom.col) +  # Use custom colors for
lines
  xlab(bquote(Time~(Weeks)))+
  ylab(bquote(Normalized~absolute~abundance))+
  theme_classic()+
  ggtitle("Cluster 2")+
  theme(
  title = element_text(size = 7, color="black"),
  axis.text = element_text(size = 7, color="black"),
  axis.title = element_text(size = 7, color="black"),
  axis.ticks= element_line(color = "black", linewidth=0.25),
  legend.text = element_text(size = 6),
  legend.title = element_text(size = 6),
  legend.key.size = unit(3, "mm"),
  legend.position = c(0.20, 0.80)
)

ggsave(filename = "C2.tiff", dpi=600, C2, width = 90, height = 60, units = "mm")
```

```
subset_cluster3 <- subset(normalized_df, SpecificValue == "3")

C3=ggplot(subset_cluster3, aes(x = WeekOfSampling, y = Normalized_AA,  color = label, legend=
label, group = OTU)) +
  geom_line(size=1) +
  scale_color_manual("Taxonomic information", values = custom.col) +  # Use custom colors for
lines
  xlab(bquote(Time~(Weeks)))+
  ylab(bquote(Normalized~absolute~abundance))+
  theme_classic() +
  ggtitle("Cluster 3") +
  theme(
  title = element_text(size = 7, color="black"),
  axis.text = element_text(size = 7, color="black"),
  axis.title = element_text(size = 7, color="black"),
  axis.ticks= element_line(color = "black", linewidth=0.25),
  legend.text = element_text(size = 6),
  legend.title = element_text(size = 6),
  legend.key.size = unit(3, "mm"),
  legend.position = c(0.3, 0.7)
)

ggsave(filename = "C3.tiff", dpi=600, C3, width = 90, height = 60, units = "mm")
```

```
subset_cluster4 <- subset(normalized_df, SpecificValue == "4")

C4=ggplot(subset_cluster4, aes(x = WeekOfSampling, y = Normalized_AA,  color = label, legend=
label, group = OTU)) +
  geom_line(size=1) +
  scale_color_manual("Taxonomic information", values = custom.col) +  # Use custom colors for
lines
  xlab(bquote(Time~(Weeks)))+
  ylab(bquote(Normalized~absolute~abundance))+
  theme_classic() +
  ggtitle("Cluster 4") +
  theme(
  title = element_text(size = 7, color="black"),
  axis.text = element_text(size = 7, color="black"),
  axis.title = element_text(size = 7, color="black"),
  axis.ticks= element_line(color = "black", linewidth=0.25),
  legend.text = element_text(size = 5),
  legend.title = element_text(size = 5),
  legend.key.size = unit(2, "mm"),
  legend.position = c(0.2, 0.8)
)

ggsave(filename = "C4.tiff", dpi=600, C4, width = 90, height = 60, units = "mm")
```

```
subset_cluster5 <- subset(normalized_df, SpecificValue == "5")

C5=ggplot(subset_cluster5, aes(x = WeekOfSampling, y = Normalized_AA,  color = label, legend=
label, group = OTU)) +
  geom_line(size=1) +
  scale_color_manual("Taxonomic information", values = custom.col) +  # Use custom colors for
lines
  xlab(bquote(Time~(Weeks)))+
  ylab(bquote(Normalized~absolute~abundance))+
  theme_classic() +
  ggtitle("Cluster 5")+
  theme(
  title = element_text(size = 7, color="black"),
  axis.text = element_text(size = 7, color="black"),
  axis.title = element_text(size = 7, color="black"),
  axis.ticks= element_line(color = "black", linewidth=0.25),
  legend.text = element_text(size = 6),
  legend.title = element_text(size = 6),
  legend.key.size = unit(3, "mm"),
  legend.position = c(0.6, 0.2)
)


ggsave(filename = "C5.tiff", dpi=600, C5, width = 90, height = 60, units = "mm")
```

```
subset_cluster6 <- subset(normalized_df, SpecificValue == "6")

C6=ggplot(subset_cluster6, aes(x = WeekOfSampling, y = Normalized_AA,  color = label, legend=
label, group = OTU)) +
  geom_line(size=1) +
  scale_color_manual("Taxonomic information", values = custom.col) +  # Use custom colors for
lines
  xlab(bquote(Time~(Weeks)))+
  ylab(bquote(Normalized~absolute~abundance))+
  theme_classic()+
  theme(
  title = element_text(size = 7, color="black"),
  axis.text = element_text(size = 7, color="black"),
  axis.title = element_text(size = 7, color="black"),
  axis.ticks= element_line(color = "black", linewidth=0.25),
  legend.text = element_text(size = 6),
  legend.title = element_text(size = 6),
  legend.key.size = unit(3, "mm"),
  legend.position = c(0.8, 0.9)
)

ggsave(filename = "C6.tiff", dpi=600, C6, width = 90, height = 60, units = "mm")
```

```
subset_cluster7 <- subset(normalized_df, SpecificValue == "7")

C7=ggplot(subset_cluster7, aes(x = WeekOfSampling, y = Normalized_AA, color = label, legend=l
abel, group = OTU)) +
  geom_line(size=1) +
  scale_color_manual("Taxonomic information", values = custom.col) +  # Use custom colors for
lines
  xlab(bquote(Time~(Weeks)))+
  ylab(bquote(Normalized~absolute~abundance))+
  theme_classic()+
  ggtitle("Cluster 7")+
  theme(
  title = element_text(size = 7, color="black"),
  axis.text = element_text(size = 7, color="black"),
  axis.title = element_text(size = 7, color="black"),
  axis.ticks= element_line(color = "black", linewidth=0.25),
  legend.text = element_text(size = 6),
  legend.title = element_text(size = 6),
  legend.key.size = unit(3, "mm"),
  legend.position = c(0.8, 0.8)
)


ggsave(filename = "C7.tiff", dpi=600, C7, width = 90, height = 60, units = "mm")
```

```
subset_cluster8 <- subset(normalized_df, SpecificValue == "8")

C8=ggplot(subset_cluster8, aes(x = WeekOfSampling, y = Normalized_AA,  color = label, legend=
label, group = OTU)) +
  geom_line(size=1) +
  scale_color_manual("Taxonomic information", values = custom.col) +  # Use custom colors for
lines
  xlab(bquote(Time~(Weeks)))+
  ylab(bquote(Normalized~absolute~abundance))+
  theme_classic()+
  ggtitle("Cluster 8")+
  theme(
  title = element_text(size = 7, color="black"),
  axis.text = element_text(size = 7, color="black"),
  axis.title = element_text(size = 7, color="black"),
  axis.ticks= element_line(color = "black", linewidth=0.25),
  legend.text = element_text(size = 6),
  legend.title = element_text(size = 6),
  legend.key.size = unit(3, "mm"),
  legend.position = c(0.8, 0.9)
)

ggsave(filename = "C8.tiff", dpi=600, C8, width = 90, height = 60, units = "mm")
```

```
subset_cluster9 <- subset(normalized_df, SpecificValue == "9")

C9=ggplot(subset_cluster9, aes(x = WeekOfSampling, y = Normalized_AA, color = label, legend=l
abel, group = OTU)) +
  geom_line(size=1) +
  scale_color_manual("Taxonomic information", values = custom.col) +  # Use custom colors for
lines
  xlab(bquote(Time~(Weeks)))+
  ylab(bquote(Normalized~absolute~abundance))+
  theme_classic()+
  ggtitle("Cluster 9")+
  theme(
  title = element_text(size = 7, color="black"),
  axis.text = element_text(size = 7, color="black"),
  axis.title = element_text(size = 7, color="black"),
  axis.ticks= element_line(color = "black", linewidth=0.25),
  legend.text = element_text(size = 6),
  legend.title = element_text(size = 6),
  legend.key.size = unit(3, "mm"),
  legend.position = c(0.8, 0.9)
)

ggsave(filename = "C9.tiff", dpi=600, C9, width = 90, height = 60, units = "mm")
```

```
subset_cluster10 <- subset(normalized_df, SpecificValue == "10")

C10=ggplot(subset_cluster10, aes(x = WeekOfSampling, y = Normalized_AA,  color = label, legen
d=label, group = OTU)) +
  geom_line(size=1) +
  scale_color_manual("Taxonomic information", values = custom.col) +  # Use custom colors for
lines
  xlab(bquote(Time~(Weeks)))+
  ylab(bquote(Normalized~absolute~abundance))+
  theme_classic()+
  ggtitle("Cluster 10")+
  theme(
  title = element_text(size = 7, color="black"),
  axis.text = element_text(size = 7, color="black"),
  axis.title = element_text(size = 7, color="black"),
  axis.ticks= element_line(color = "black", linewidth=0.25),
  legend.text = element_text(size = 6),
  legend.title = element_text(size = 6),
  legend.key.size = unit(3, "mm"),
  legend.position = c(0.85, 0.9)
)
ggsave(filename = "C10.tiff", dpi=600, C10, width = 90, height = 60, units = "mm")
```

```
subset_cluster11 <- subset(normalized_df, SpecificValue == "11")

C11=ggplot(subset_cluster11, aes(x = WeekOfSampling, y = Normalized_AA,  color = label, legen
d=label, group = OTU)) +
  geom_line(size=1) +
  scale_color_manual("Taxonomic information", values = custom.col) +  # Use custom colors for
lines
  xlab(bquote(Time~(Weeks)))+
  ylab(bquote(Normalized~absolute~abundance))+
  theme_classic()+
  ggtitle("Cluster 11")+
  theme(
  title = element_text(size = 7, color="black"),
  axis.text = element_text(size = 7, color="black"),
  axis.title = element_text(size = 7, color="black"),
  axis.ticks= element_line(color = "black", linewidth=0.25),
  legend.text = element_text(size = 6),
  legend.title = element_text(size = 6),
  legend.key.size = unit(3, "mm"),
  legend.position = c(0.75, 0.9)
)
ggsave(filename = "C11.tiff", dpi=600, C11, width = 90, height = 60, units = "mm")
```

```
subset_cluster12 <- subset(normalized_df, SpecificValue == "12")

C12=ggplot(subset_cluster12, aes(x = WeekOfSampling, y = Normalized_AA,  color = label, legen
d=label, group = OTU)) +
  geom_line(size=1) +
  scale_color_manual("Taxonomic information", values = custom.col) +  # Use custom colors for
lines
  xlab(bquote(Time~(Weeks)))+
  ylab(bquote(Normalized~absolute~abundance))+
  theme_classic()+
  ggtitle("Cluster 12")+
  theme(
  title = element_text(size = 7, color="black"),
  axis.text = element_text(size = 7, color="black"),
  axis.title = element_text(size = 7, color="black"),
  axis.ticks= element_line(color = "black", linewidth=0.25),
  legend.text = element_text(size = 6),
  legend.title = element_text(size = 6),
  legend.key.size = unit(3, "mm"),
  legend.position = c(0.2, 0.75)
)
ggsave(filename = "C12.tiff", dpi=600, C12, width = 90, height = 60, units = "mm")
```

```
subset_cluster13 <- subset(normalized_df, SpecificValue == "13")

C13=ggplot(subset_cluster13, aes(x = WeekOfSampling, y = Normalized_AA,  color = label, legen
d=label, group = OTU)) +
  geom_line(size=1) +
  scale_color_manual("Taxonomic information", values = custom.col) +  # Use custom colors for
lines
  xlab(bquote(Time~(Weeks)))+
  ylab(bquote(Normalized~absolute~abundance))+
  theme_classic()+
  ggtitle("Cluster 13")+
  theme(
  title = element_text(size = 7, color="black"),
  axis.text = element_text(size = 7, color="black"),
  axis.title = element_text(size = 7, color="black"),
  axis.ticks= element_line(color = "black", linewidth=0.25),
  legend.text = element_text(size = 6),
  legend.title = element_text(size = 6),
  legend.key.size = unit(3, "mm"),
  legend.position = c(0.2, 0.9)
)
ggsave(filename = "C13.tiff", dpi=600, C13, width = 90, height = 60, units = "mm")
```

Comparing D0 water and week 1 biofilm

In this last section, we compare the water sample on Day 0 of the reactor commissioning with the biofilms after one week, in order to identify the pioneer genuses that started the biofilm formation process.

```
BW <- subset_samples(BtemporalW, WeekOfSampling=="0"|WeekOfSampling=="1")
BW <- prune_taxa(taxa_sums(BW )>0, BW )
BW_relabund <- transform(BW, "compositional")
```

```
df_BW <- psmelt(BW_relabund)
df_BW <- df_BW %>% group_by(OTU) %>% mutate(medianRA = median(Abundance)) %>% filter(medianRA
>= 0.01)
```

```
ggplot(df_BW, (aes(x=SampleType, y=Abundance, group=SampleType)))+
  geom_boxplot()+
  facet_wrap(~OTU, scales = "free_y")
```

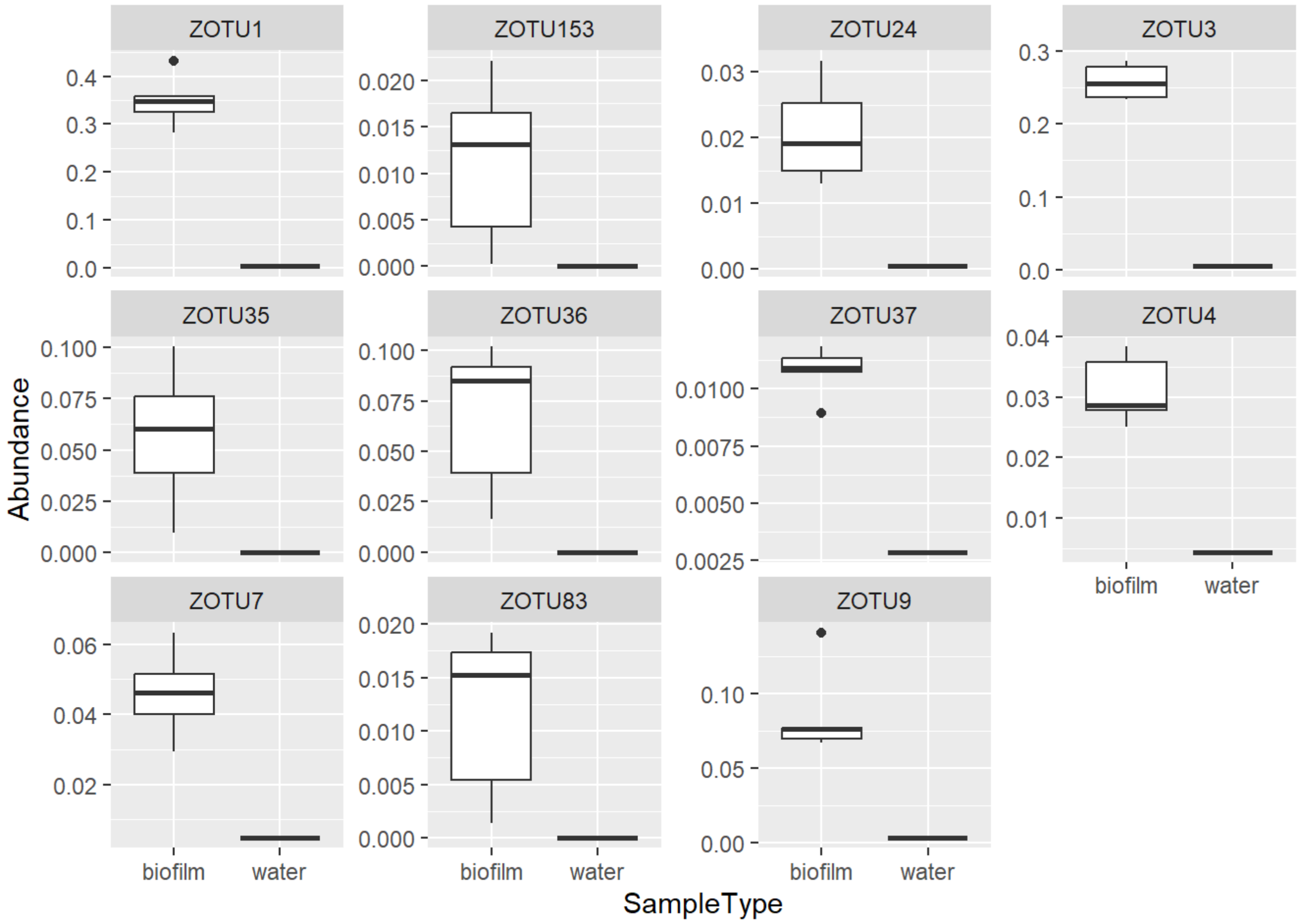


```
# Subset the dataframe to get data for sample 1
sample1_data <- subset(df_BW, SampleType == "biofilm")

# Subset the dataframe to get data for sample 2
sample2_data <- subset(df_BW, SampleType == "water")

# Merge the two data subsets based on the 'Zotu' column
merged_data <- merge(sample1_data, sample2_data, by = "OTU", suffixes = c("_biofilm", "_wate
r"))

# Perform the division of zotu abundances
merged_data$Abundance_div <- merged_data$Abundance_biofilm / merged_data$Abundance_water
```

```
taxInfo <- df_BW %>% select(OTU, Phylum, Class, Order, Family, Genus) %>% distinct() #Species
merged_data <- merged_data %>% left_join(taxInfo, by = "OTU")
merged_data_summar <- merged_data %>% mutate(label = case_when(is.na(Genus) ~ OTU,
                                                               Genus == "uncultured" ~ paste
("Uncult.", Family),
                                                               TRUE ~ Genus)) %>%
group_by(label) %>% summarise(medianRA_div = median(Abundance_div))
```

```
bar_plot <- ggplot(merged_data_summar, aes(x = reorder(label, medianRA_div), y = medianRA_di
v)) +
  geom_bar(stat = "identity", fill = "#4B4A4C") +
  labs(x = NULL,
       y = "Factor difference in relative abundance")+
  theme_classic() + scale_y_log10()+
  theme_few()+
  theme(
    axis.text = element_text(size = 7, color = "black"),
    axis.title = element_text(size = 7, color = "black"),
    axis.ticks = element_line(color = "black", linewidth = 0.25))

# Rotate x-axis labels for better readability (optional)
bar_plot <- bar_plot + theme(axis.text.x = element_text(angle = 45, hjust = 1))

# Print the bar plot
print(bar_plot)
```

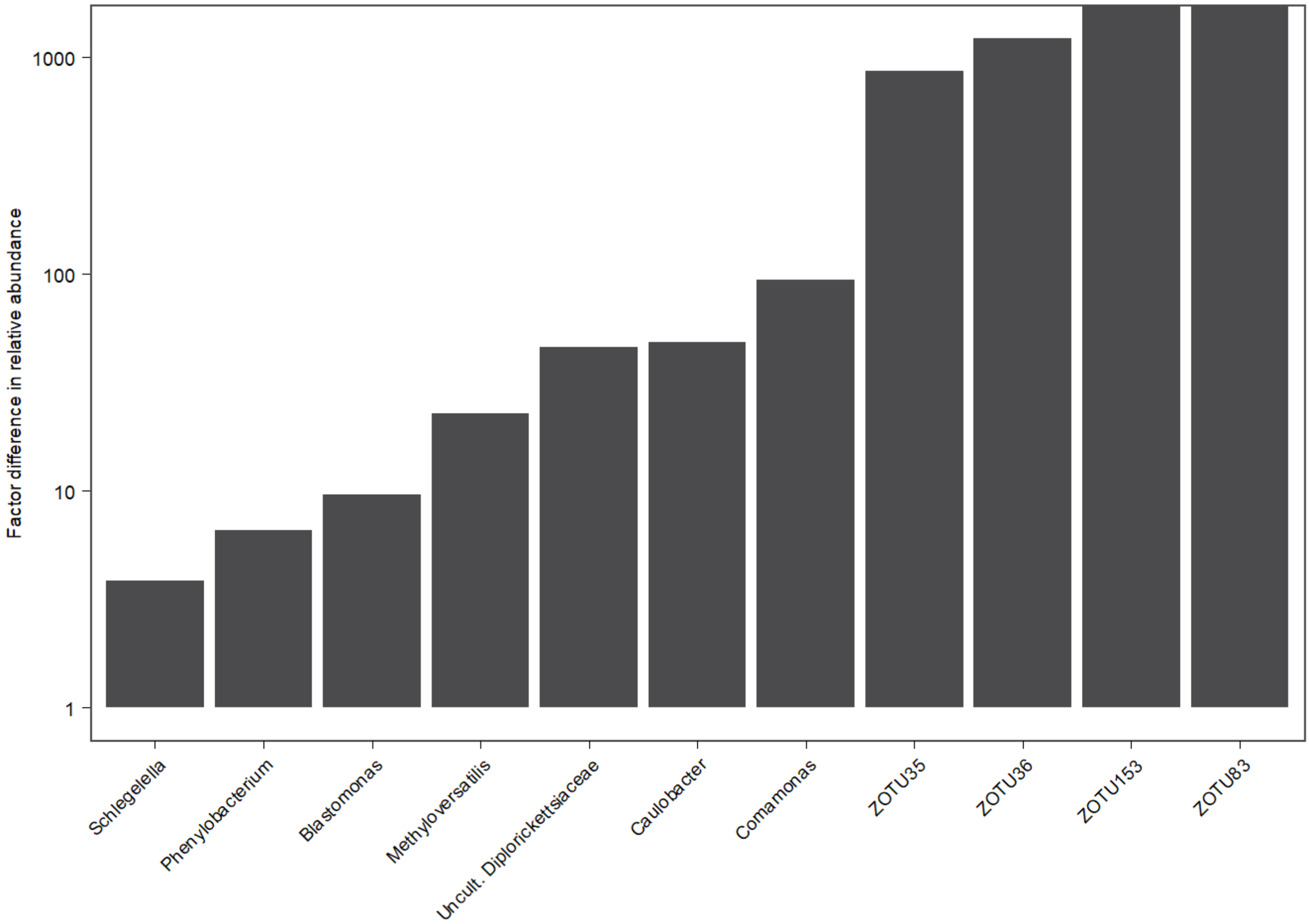


```
ggsave(filename = "bar_plot.pdf", dpi=600, bar_plot, width=50, height = 70, units="mm")
```

Save and load

```
#save.image("2024-01-15_16S_CMargot.RData")
load("2024-01-15_16S_CMargot.RData")
```

Compatibility Check

```
sink("SessionInfo.txt")
  sessionInfo()
```

```
## R version 4.3.1 (2023-06-16 ucrt)
## Platform: x86_64-w64-mingw32/x64 (64-bit)
## Running under: Windows 10 x64 (build 19045)
## 
## Matrix products: default
## 
## 
## locale:
## [1] LC_COLLATE=German_Switzerland.utf8  LC_CTYPE=German_Switzerland.utf8   
## [3] LC_MONETARY=German_Switzerland.utf8 LC_NUMERIC=C                       
## [5] LC_TIME=German_Switzerland.utf8    
## 
## time zone: Europe/Zurich
## tzcode source: internal
## 
## attached base packages:
## [1] stats     graphics  grDevices utils     datasets  methods   base     
## 
## other attached packages:
##  [1] NbClust_3.0.1     TSclust_1.3.1     cluster_2.1.4     pdc_1.0.3        
##  [5] ranacapa_0.1.0    decontam_1.20.0   ggthemes_4.2.4    microbiome_1.22.0
##  [9] phyloseq_1.44.0   vegan_2.6-4       lattice_0.21-8    permute_0.9-7    
## [13] ggpubr_0.6.0      ggplot2_3.4.2     tidyr_1.3.0       dplyr_1.1.2      
## [17] descr_1.1.7      
## 
## loaded via a namespace (and not attached):
##   [1] rstudioapi_0.15.0       jsonlite_1.8.7          magrittr_2.0.3         
##   [4] farver_2.1.1            rmarkdown_2.23          zlibbioc_1.46.0        
##   [7] ragg_1.2.5              vctrs_0.6.3             multtest_2.56.0        
##  [10] RCurl_1.98-1.12         base64enc_0.1-3         rstatix_0.7.2          
##  [13] htmltools_0.5.5         curl_5.0.1              broom_1.0.5            
##  [16] Rhdf5lib_1.22.0         rhdf5_2.44.0            TTR_0.24.3             
##  [19] sass_0.4.7              KernSmooth_2.23-22      bslib_0.5.1            
##  [22] htmlwidgets_1.6.2       plyr_1.8.8              zoo_1.8-12             
##  [25] cachem_1.0.8            misc3d_0.9-1            igraph_1.5.1           
##  [28] lifecycle_1.0.3         iterators_1.0.14        pkgconfig_2.0.3        
##  [31] Matrix_1.6-0            R6_2.5.1                fastmap_1.1.1          
##  [34] GenomeInfoDbData_1.2.10 digest_0.6.31           colorspace_2.1-0       
##  [37] S4Vectors_0.38.1        textshaping_0.3.6       labeling_0.4.2         
##  [40] fansi_1.0.4             abind_1.4-5             mgcv_1.9-0             
##  [43] compiler_4.3.1          proxy_0.4-27            withr_2.5.0            
##  [46] backports_1.4.1         tseries_0.10-54         carData_3.0-5          
##  [49] highr_0.10              Rttf2pt1_1.3.12         ggsignif_0.6.4         
##  [52] MASS_7.3-60             biomformat_1.28.0       locpol_0.8.0           
##  [55] tools_4.3.1             lmtest_0.9-40           ape_5.7-1              
##  [58] quantmod_0.4.24         extrafontdb_1.0         nnet_7.3-19            
##  [61] glue_1.6.2              quadprog_1.5-8          nlme_3.1-163           
##  [64] rhdf5filters_1.12.1     grid_4.3.1              Rtsne_0.16             
##  [67] reshape2_1.4.4          ade4_1.7-22             generics_0.1.3         
##  [70] gtable_0.3.3            class_7.3-22            data.table_1.14.8      
##  [73] car_3.1-2               utf8_1.2.3              XVector_0.40.0         
##  [76] BiocGenerics_0.46.0     foreach_1.5.2           pillar_1.9.0           
##  [79] stringr_1.5.0           splines_4.3.1           survival_3.5-5         
##  [82] tidyselect_1.2.0        Biostrings_2.68.1       knitr_1.43             
##  [85] urca_1.3-3              IRanges_2.34.1          forecast_8.21          
```

```
##  [88] stats4_4.3.1           xfun_0.39               Biobase_2.60.0
##  [91] timeDate_4022.108      stringi_1.7.12          yaml_2.3.7
##  [94] evaluate_0.21          codetools_0.2-19        dtw_1.23-1
##  [97] tcltk_4.3.1            extrafont_0.19          tibble_3.2.1
## [100] cli_3.6.1              xtable_1.8-4            systemfonts_1.0.4
## [103] munsell_0.5.0          jquerylib_0.1.4         Rcpp_1.0.10
## [106] GenomeInfoDb_1.36.1    parallel_4.3.1          fracdiff_1.5-2
## [109] rgl_1.2.1              clv_0.3-2.3             bitops_1.0-7
## [112] xts_0.13.1             scales_1.2.1            purrr_1.0.1
## [115] crayon_1.5.2           rlang_1.1.1             longitudinalData_2.4.5
```

```
sink()
```

18S analysis

Céline Margot

2023-07-03

Introduction

The code below contains all steps performed for the 18S rRNA gene amplicon sequencing analysis and figure production for our paper "Dynamics of drinking water biofilm formation associated with Legionella spp. colonization".

Setup

Data Import

Load the files including a mapfile containing the DNA concentrations before pooling.

```
otufile.zotu <- "p881_run221028_18S_ZOTU_Count_Sintax.txt"
mapfile.zotu <- "18SMapfile2.txt"
treefile.zotu  <- "p881_run221028_18S_ZOTU_MSA.tre"
refseqfile.zotu <- "p881_run221028_18S_ZOTU.fa.gz"

d.zotu <- import_qiime(otufilename=otufile.zotu,
                       mapfilename=mapfile.zotu,
                       treefilename=treefile.zotu)
```

```
## Processing map file...
## Processing otu/tax file...
## Reading file into memory prior to parsing...
## Detecting first header line...
## Header is on line 1
## Converting input file to a table...
## Defining OTU table...
## Parsing taxonomy table...
## Processing phylogenetic tree...
##  p881_run221028_18S_ZOTU_MSA.tre ...
```

```
d.zotu
```

```
## phyloseq-class experiment-level object
## otu_table()   OTU Table:         [ 1238 taxa and 69 samples ]
## sample_data() Sample Data:       [ 69 samples by 17 sample variables ]
## tax_table()   Taxonomy Table:    [ 1238 taxa by 7 taxonomic ranks ]
## phy_tree()    Phylogenetic Tree: [ 1238 tips and 1237 internal nodes ]
```

Control checks

Library Prep positive control: Amoebae genomic DNA (ATCC standard)

```
d.zotu <- prune_taxa(taxa_sums(d.zotu) > 0,d.zotu)
library_positive_ctrls <- subset_samples(d.zotu, ExperimentType=="Amoebae") #  Library prep p
ositive controls = Amoebae genomic DNA (ATCC standard)
library_positive_ctrls  <- prune_taxa(taxa_sums(library_positive_ctrls ) > 0,library_positive
_ctrls )
sample_sums(library_positive_ctrls)
```

```
## Gen-01 Gen-02
## 163865 236384
```

```
taxa_sums(library_positive_ctrls)
```

```
## ZOTU857  ZOTU26  ZOTU10  ZOTU11  ZOTU32 ZOTU109 ZOTU195   ZOTU8  ZOTU54   ZOTU1
##      23       3       4       1       1       1       1       2       2      76
## ZOTU137   ZOTU7  ZOTU17   ZOTU4   ZOTU9 ZOTU528  ZOTU23  ZOTU42 ZOTU174 ZOTU113
##       1       1       2      13       4      41       3       1       1       2
##  ZOTU52  ZOTU36 ZOTU111  ZOTU16   ZOTU2 ZOTU140  ZOTU22   ZOTU3   ZOTU5  ZOTU12
##       2       3       2  117160      61       1       6      41      22  282766
##  ZOTU14
##       2
```

```
plot_composition(library_positive_ctrls, level="Genus")
```

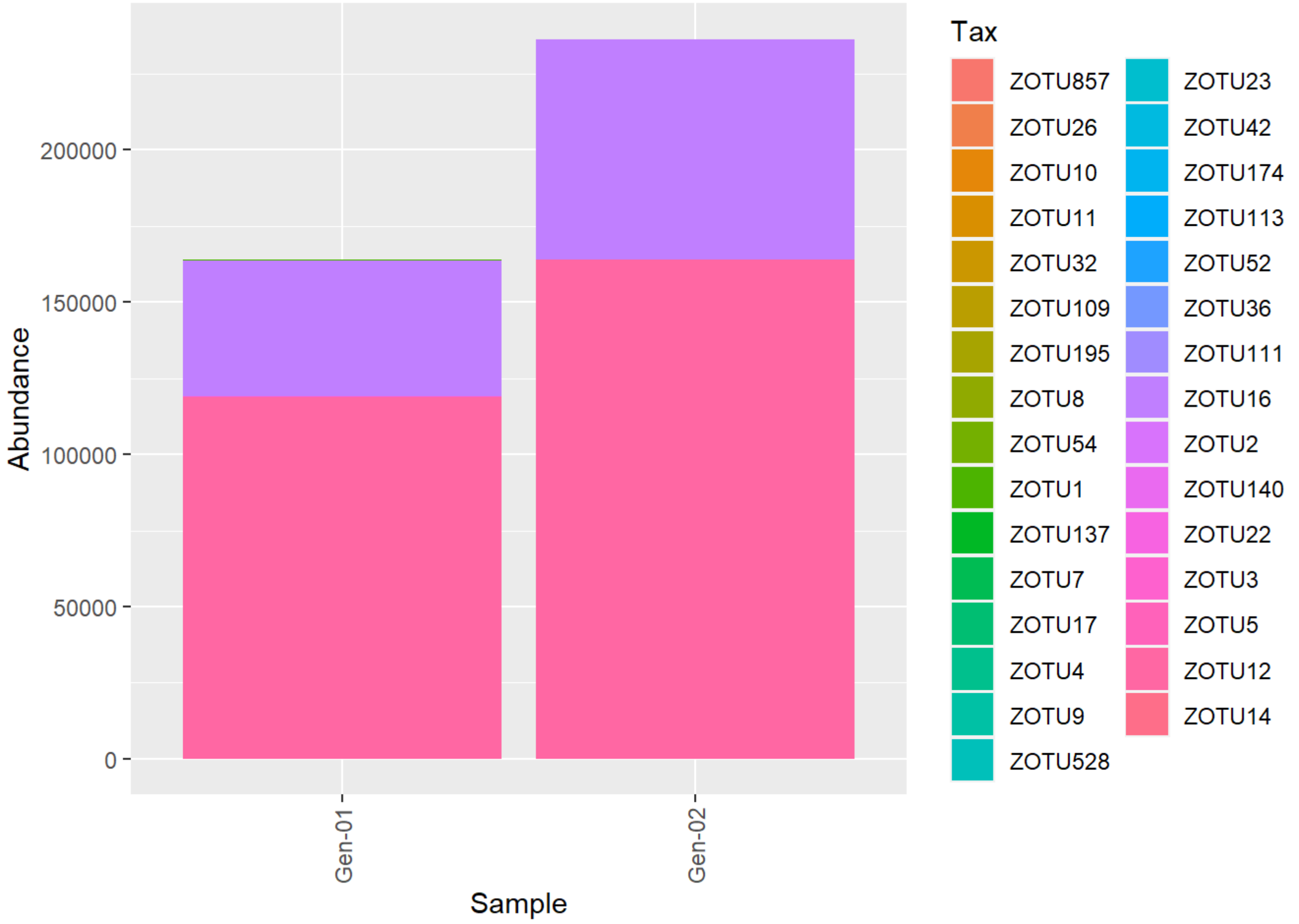

Conclusions: two zOTUs dominate: zOTU12 (Eukaryota:plas) and zOTU16 (Acanthamoeba_sp) - zOTU16 (Acanthamoeba_sp) –> as expected - zOTU12 (Eukaryota:plas) –> contaminant

Library Prep negative controls

```
d.zotu <- prune_taxa(taxa_sums(d.zotu) > 0,d.zotu)
library_negative_ctrls <- subset_samples(d.zotu, SampleType=="control" & SampleCategory=="neg
ative" & Column=="Step2")
library_negative_ctrls  <- prune_taxa(taxa_sums(library_negative_ctrls ) > 0,library_negative
_ctrls )
sample_sums(library_negative_ctrls)
```

```
## NTC-01 NTC-02 NTC-03 NTC-04 NTC-05 NTC-06 NTC-07 NTC-08 NTC-09 NTC-10 NTC-11
##    143    395     58    215     99   4405    413    850     87     68     18
## NTC-12
##     33
```

```
taxa_sums(library_negative_ctrls)
```

```
##    ZOTU57   ZOTU199   ZOTU106  ZOTU1031    ZOTU21    ZOTU87    ZOTU19    ZOTU26
##         1         1         1         1         9         1         4         6
##    ZOTU13    ZOTU28    ZOTU40    ZOTU10    ZOTU37    ZOTU11   ZOTU268   ZOTU635
##        11         7         1        45         7        45         1         1
##   ZOTU104    ZOTU66    ZOTU32    ZOTU86    ZOTU24    ZOTU18   ZOTU109   ZOTU195
##         1         1         5         1        18         8        82         7
##     ZOTU8    ZOTU54   ZOTU321    ZOTU53    ZOTU75    ZOTU70     ZOTU1   ZOTU354
##        58       477         1         3         7         1       635         1
##   ZOTU890   ZOTU350    ZOTU15    ZOTU80    ZOTU46    ZOTU82    ZOTU25   ZOTU136
##        18         1        29         1         2        25        68         1
##    ZOTU39    ZOTU35    ZOTU20     ZOTU6     ZOTU7    ZOTU30    ZOTU17    ZOTU34
##         1         3        38        33        30         6        12         5
##    ZOTU72     ZOTU4     ZOTU9   ZOTU179   ZOTU187   ZOTU102    ZOTU51    ZOTU23
##         2       173        42         1        10         1         2        13
##   ZOTU204    ZOTU42   ZOTU174   ZOTU303   ZOTU126   ZOTU100    ZOTU79    ZOTU27
##       187       694         1       219         1         1         1         4
##    ZOTU52    ZOTU44    ZOTU45   ZOTU290   ZOTU338    ZOTU36   ZOTU107   ZOTU217
##      1865         3         2         1         1         5         2         1
##    ZOTU67    ZOTU73   ZOTU171    ZOTU16    ZOTU31     ZOTU2    ZOTU29    ZOTU33
##       677         1         1        28         3       417        11         6
##    ZOTU43    ZOTU22    ZOTU38     ZOTU3     ZOTU5    ZOTU12    ZOTU58    ZOTU81
##         2        25         4       335       217        74         2         3
##    ZOTU41    ZOTU14
##         1        24
```

```
library_negative_ctrls  <- prune_taxa(taxa_sums(library_negative_ctrls ) > 50,library_negativ
e_ctrls ) #let's only take zOTUs with more than 50 reads for plotting
plot_composition(library_negative_ctrls, level="Genus")
```

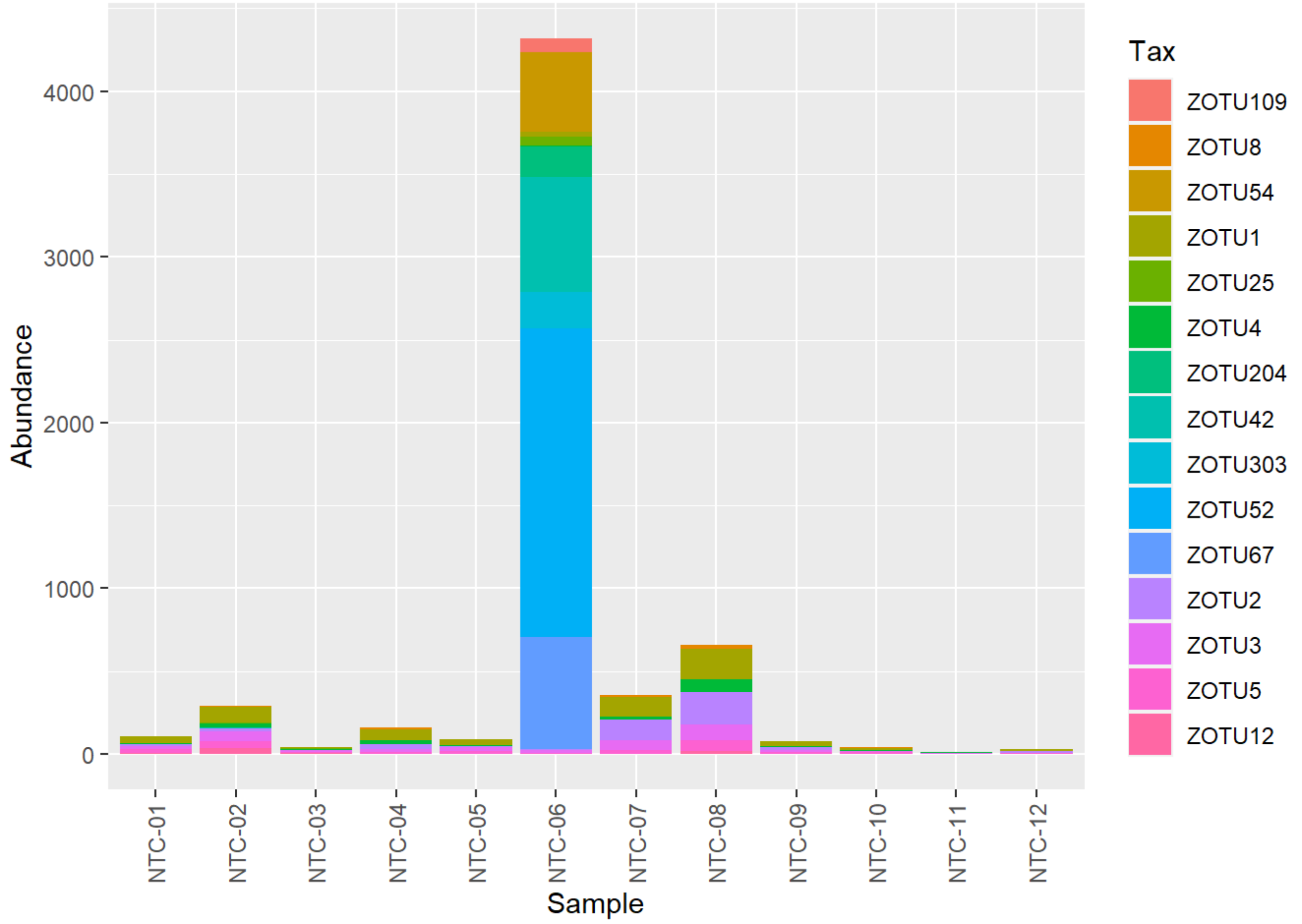

Conclusions: In general low reads number, NTC-06 has highest read count with 4405 reads. Dominant taxa founds accross all negatives: zOTU52 with 1865 reads, mostly presnet in NTC-06. We do find some of our sample zOTUs in the negative controls. However, all negative controls had low DNA concentration before preparing the pool. The samples were then pooled to equimolar concentrations, which means higher volume of negative control was added to the pool that underwent sequencing. Since the DNA concentrations before pooling are low, we assume that contamination in the negative control is low.

Decontamination

```
concentration_values <- sample_data(d.zotu)$Concentration_2 # Access the concentration values from sample_data slot
low_concentration_samples <- concentration_values <= 0 # Identify samples with concentration <= 0
d.zotu <- prune_samples(!low_concentration_samples, d.zotu)# Exclude the identified samples from the phyloseq object
```

This is our base phyloseq for subsequent decontamination analysis. Important is that we still have all types of controls. Depending on the analysis, we will remove the controls by case of decontamination process.

Assign each control to a different category in a new column called “ControlType”

Now that we have a dataset ready to use, let’s first visualize sample read numbers to see the distribution of sequencing sampling depth. For this, we add a column to the dataset which specifies which controls are library prep negatives, which controls are library prep positive, which controls are extraction negative and which controls are extraction positive and finally which control are process control negatives.

```
sample_data(d.zotu)$ControlType <- "NotControl" # all other samples are labelled as "NotControl"

sample_data(d.zotu)$ControlType[sample_data(d.zotu)$SampleCategory == "negative" & sample_data(d.zotu)$ExperimentType == "LibraryPrep"] <- "LibraryPrepNeg"
```

Check NMDS with Jaccard distance

```
ord_x <- ordinate(d.zotu, "NMDS", "jaccard")
```

```
## Square root transformation
## Wisconsin double standardization
## Run 0 stress 0.1631279
## Run 1 stress 0.132571
## ... New best solution
## ... Procrustes: rmse 0.1267838  max resid 0.4818837
## Run 2 stress 0.1502217
## Run 3 stress 0.1279546
## ... New best solution
## ... Procrustes: rmse 0.06241967  max resid 0.2239751
## Run 4 stress 0.1317369
## Run 5 stress 0.3993416
## Run 6 stress 0.1409967
## Run 7 stress 0.1395
## Run 8 stress 0.149971
## Run 9 stress 0.1393093
## Run 10 stress 0.127129
## ... New best solution
## ... Procrustes: rmse 0.06209339  max resid 0.2741109
## Run 11 stress 0.1573007
## Run 12 stress 0.1930777
## Run 13 stress 0.1504164
## Run 14 stress 0.15196
## Run 15 stress 0.132978
## Run 16 stress 0.1528731
## Run 17 stress 0.1486539
## Run 18 stress 0.1279543
## Run 19 stress 0.1533659
## Run 20 stress 0.1314093
## *** Best solution was not repeated -- monoMDS stopping criteria:
##      1: no. of iterations >= maxit
##     19: stress ratio > sratmax
```

```
p_ord_x= plot_ordination(d.zotu, ord_x, type="samples", color="ControlType")+ geom_point(size=2)  +theme_few()
```

Inspect library sizes

Let’s put the data in a dataframe an then plot library sized

```
df_Euka <- as.data.frame(sample_data(d.zotu)) # Put sample_data into a ggplot-friendly data.f
rame
df_Euka$LibrarySize <- sample_sums(d.zotu)
df_Euka <- df_Euka[order(df_Euka$LibrarySize),]
df_Euka$Index <- seq(nrow(df_Euka))
ggplot(data=df_Euka, aes(x=Index, y=LibrarySize, color=ControlType)) + geom_point()
```

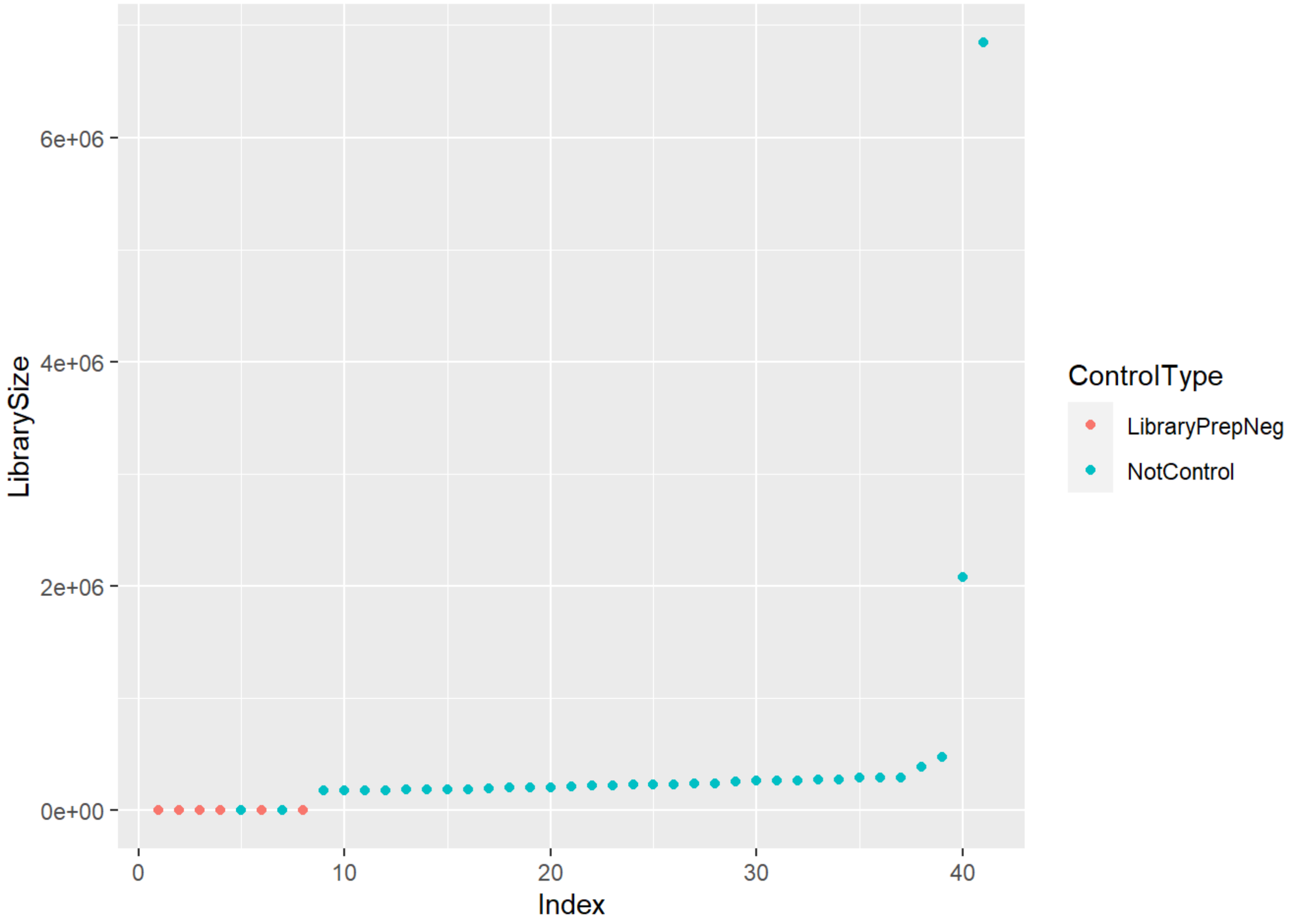

We can see that the negative controls are very low which is very good. We have 6 of them that are > or = to 0 which means we can use them in the combined method for the decontamination process. We also see that 2 samples have way more reads than the rest and that two samples which are not negative controls a very low read count.

To solve these two isses: 1) Identify samples with low read count, check if low concentration before pooling, remove samples with low read count.

```
sample_sums(d.zotu)
```

```
##  A-1-02  A-1-03  A-1-04  A-1-05  A-1-06  A-1-07  A-1-08  A-1-11  A-1-12  A-1-13
##  263889  274041  206050  241101 2080421  233219  254573  206015  202393  288274
##  A-1-14  A-1-15  A-1-16  A-1-17  B-1-01  B-1-03  B-1-04  B-1-05  B-1-07  B-1-11
##  479250     789  211732  188258  181857  391161  182537  232514 6844032  240268
##  B-1-12  B-1-13  B-1-14  B-1-15  B-1-17  B-1-18  C-1-03  C-1-04  C-1-05  C-1-07
##  183201  268435  232343  178902  221783     128  271480  189921  224863  268875
##  C-1-11  C-1-12  C-1-13  C-1-14  C-1-15  NTC-04  NTC-05  NTC-06  NTC-10  NTC-11
##  295912  190644  293695  193695  175422     215      99    4405      68      18
##  NTC-12
##      33
```

Samples B-1-18 and A-1-15 have low sequencing depth, and low concentration before pooling. Let's remove these samples from the analysis.

```
d.zotu <- subset_samples(d.zotu,  SampleID != "A-1-15" & SampleID != "B-1-18")# remove sample
s with low dna concentration after library preparation and low sequencing depth.
```

2. Since two samples have a very high read count, we can rarefy our samples to even sampling depth later.

Decontamination

Remove 0s

```
RLE <- prune_taxa(taxa_sums(d.zotu)>0, d.zotu)
RLE <- prune_samples(sample_sums(d.zotu)>0, d.zotu)
```

Compare the frequency, prevalence and combined methods on the whole dataset, using NTC controls as negatives and a threshold of 0.1

```
library(decontam)
```

```
sample_data(d.zotu)$is.neg <- sample_data(d.zotu)$ControlType == "LibraryPrepNeg"
# Frequency-based contaminant classification
RLE_f <- isContaminant((d.zotu), conc="Concentration_2", threshold=0.1, detailed=TRUE, normal
ize=TRUE, method='frequency')
# Prevalence-based contaminant classification
RLE_p <- isContaminant((d.zotu), neg="is.neg", threshold=0.1, detailed=TRUE, normalize=TRUE,
method='prevalence')
# Combined contaminant classification
RLE_c <- isContaminant((d.zotu), conc="Concentration_2", neg="is.neg", threshold=0.1, detaile
d=TRUE, normalize=TRUE, method='combined')
```

Comparison of decontamination methods

```
probcols <- data.frame(row.names=rownames(RLE_f),prob.f=RLE_f$p.freq, prob.p=RLE_p$p.prev, pr
ob.c = RLE_c$p)
#TAX2 <- cbind(TAX2[,colnames(TAXannotate)], probcols[TAX2$Id,])
# Crude comparison of frequency and prevalence contaminant assignment
table(probcols$prob.f<0.1, probcols$prob.p<0.1)
```

```
##
##         FALSE
##   FALSE   790
##   TRUE     22
```

```
table(probcols$prob.f<0.1, probcols$prob.c<0.1)
```

```
##
##         FALSE TRUE
##   FALSE   788    0
##   TRUE     20    2
```

```
table(probcols$prob.p<0.1, probcols$prob.c<0.1)
```

```
##
##         FALSE TRUE
##   FALSE   808    2
```

```
TAX=data.frame(tax_table(d.zotu))
TAXannotate <- data.frame(tax_table(d.zotu))
rownames(TAXannotate) <- TAXannotate$Id
#TAXannotate <-TAXannotate[taxa_names(bw_decontam),]

TAX2 <-cbind(TAX, probcols)
tt.class <- probcols[!is.na(TAX2$prob.c),] # 775 classified by decontam, aka "All ASVs"
TAXann <- rbind(cbind(tt.class, Score=tt.class$prob.f, Method="Frequency"),
                cbind(tt.class, Score=tt.class$prob.p, Method="Prevalence"),
                cbind(tt.class, Score=tt.class$prob.c, Method="Combined"))
TAXann$Method <- factor(TAXann$Method, levels=c("Frequency", "Prevalence", "Combined"))

histo <- ggplot(TAXann, aes(x=Score))
histo <- histo + geom_histogram() + labs(x = 'decontam Score', y='Number ASVs') +
  facet_wrap(~Method, nrow=1) +
  theme(legend.position = "bottom")
histo
```

```
## `stat_bin()` using `bins = 30`. Pick better value with `binwidth`.
```

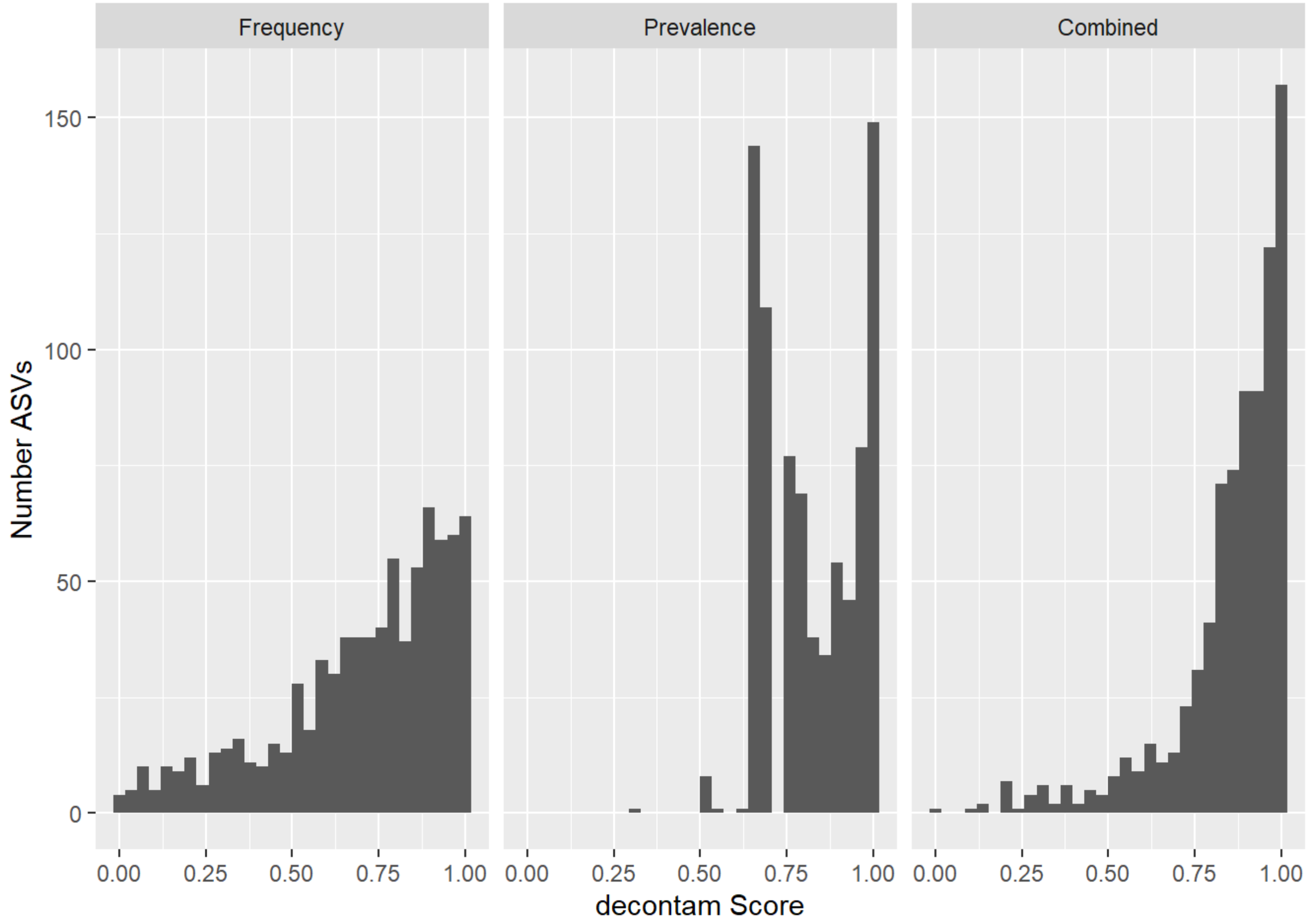

Suggest to use the combined method with a threshold of 0.5.

Analysis using a decontamination of 0.5 on the whole dataset, and the method “combined”. Negative control is the extraction control

```
sample_data(d.zotu)$is.neg <- sample_data(d.zotu)$ControlType == "LibraryPrepNeg"
contaminants <- isContaminant(d.zotu, method="combined", threshold=0.5, neg="is.neg", conc="C
oncentration_2")
table(contaminants$contaminant)
```

```
##
## FALSE  TRUE
##  1196    41
```

41 out of 1196 sequences were identified as contaminants.

Let’s remove the contaminants from our phyloseq object

```
noncontam <- prune_taxa(!contaminants$contaminant, d.zotu)
```

Let’s now try to plot a few of our main graphs

First one in line, the bray curtis graph of the temporal data.

Save and load

```
#save.image("2023-11-30_18SDecontaminated.Rdata")
load("2023-11-30_18SDecontaminated.Rdata")
```

subsetting the temporal data

```
temporal <- subset_samples(noncontam, SampleCategory=="temporal")
```

Get total read counts for samples

```
library(data.table)
```

```
##
## Attache Paket: 'data.table'
```

```
## Die folgenden Objekte sind maskiert von 'package:dplyr':
##
##     between, first, last
```

```
dat_tot <- data.table(as(sample_data(temporal), "data.frame"), TotalReads = sample_sums(tempo
ral), keep.rownames = TRUE)
```

Filtering function

```
temporal_relabund <- transform(temporal, "compositional")

limiter <- function(x){
  x >= 0.01
}

f1=filterfun_sample(limiter)
wh1=genefilter_sample(temporal_relabund,f1,A=1)
z=prune_taxa(wh1,temporal_relabund)
z
```

```
## phyloseq-class experiment-level object
## otu_table()   OTU Table:         [ 30 taxa and 33 samples ]
## sample_data() Sample Data:       [ 33 samples by 19 sample variables ]
## tax_table()   Taxonomy Table:    [ 30 taxa by 7 taxonomic ranks ]
## phy_tree()    Phylogenetic Tree: [ 30 tips and 29 internal nodes ]
```

Plot composition at the Kingdom level

```
#compo_Decontam_bw_temporal_Kingdom <- z %>%
  #aggregate_taxa(level = "Kingdom") %>%
  #transform(transform = "compositional")

custom.col=c("#8D2B36",  "#C4AA3F",   "#658782")

#compo_Decontam_bw_temporal_Kingdom <-aggregate_rare(compo_Decontam_bw_temporal_Kingdom, leve
l = "Kingdom", detection = 0.0, prevalence = 0.0)

kingdom <- aggregate_taxa(z, "Kingdom")

barplot_18S_all <- plot_composition(kingdom, level="Kingdom", group_by = "WeekOfSampling") +
  scale_fill_manual(values=custom.col) +
  labs(x = "Weeks of incubation",
       y = "Relative abundance")+
  theme(text = element_text(size = 18))+
  theme_classic()+
  theme(
  axis.text = element_text(size = 7),
  axis.title = element_text(size = 7),
  legend.text = element_text(size = 7),
  legend.title= element_text(size = 7),
  legend.key.size = unit(4, "mm"),
  axis.text.x = element_blank(), axis.ticks.x = element_blank()
  )
barplot_18S_all
```

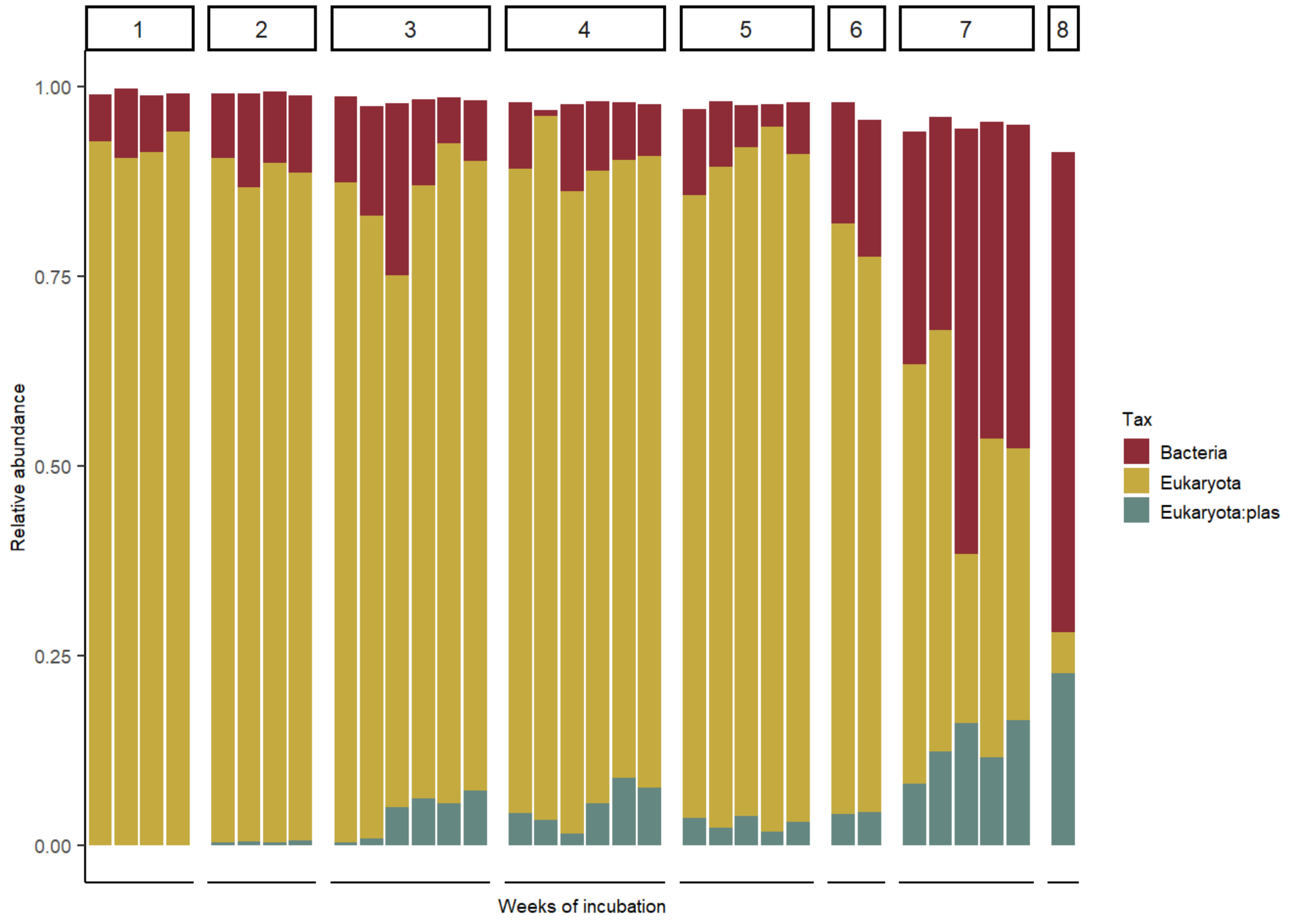

```
ggsave(filename = "barplot_18S_all.png", barplot_18S_all, width = 190, height = 90, units =
"mm")
```

Plot protisan composition

```
custom.col=c("#8D2B36",  '#d3706c',  "#C4AA3F", "#658782",  "#24604D")
Euka <- subset_taxa(z, Kingdom=="Eukaryota" & !(Phylum=="NA") & !(Phylum=="Metazoa") ) # remo
ve unassigned zOTUs and multicellular organisms
Euka_tranformed <-  transform(Euka, transform = "compositional")
p_Euka_genus <- plot_composition(Euka_tranformed ,level="OTU",
                      group_by = "WeekOfSampling", otu.sort = "abundance") +
  scale_fill_manual(values=custom.col) +
  labs(x= "Samples by week of sampling",
       y = "Relative abundance")+
  theme(text = element_text(size = 18))+
  theme_few()+
  theme(
  axis.text = element_text(size = 7),
  axis.title = element_text(size = 7),
  legend.text = element_text(size = 7),
  #legend.title= element_text(size = 7),
  #legend.key.size = unit(4, "mm"),
  axis.text.x = element_blank(), axis.ticks.x = element_blank()
  )+
  theme(
    axis.text = element_text(size = 7, color = "black"),
    axis.title = element_text(size = 7, color = "black"),
    axis.ticks = element_line(color = "black", linewidth = 0.25),
    legend.position = "none"
  )

p_Euka_genus
```

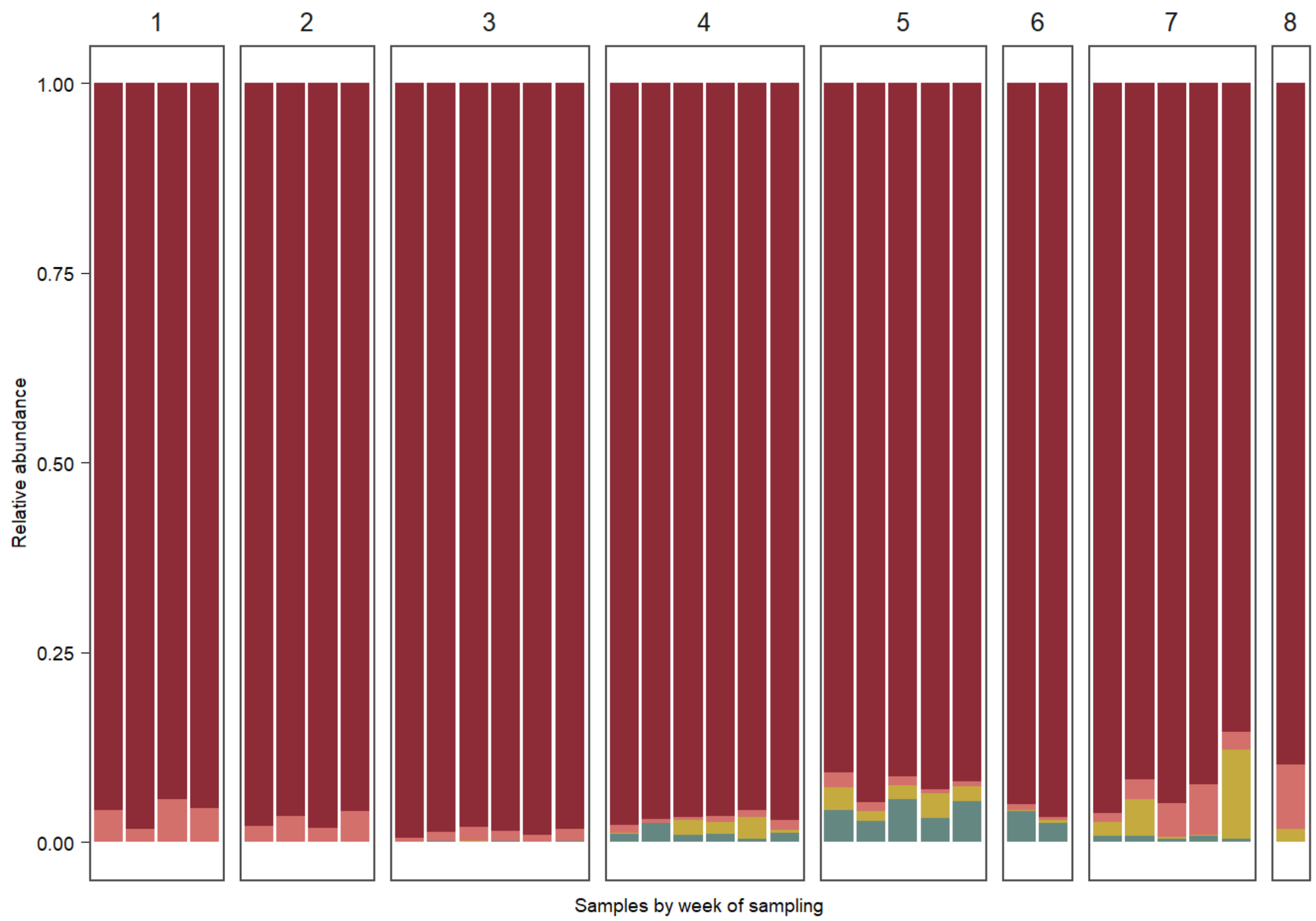


```
ggsave(filename = "p_Euka_genus_withoutL.tiff", p_Euka_genus ,dpi=600, width=90, height =55,
units="mm")
```

Calculate Percentage of kept reads for each samples

```
dat_fil <- data.table(as(sample_data(Euka), "data.frame"), KeptReads = sample_sums(Euka), kee
p.rownames = TRUE)
dat_fil <- dat_fil %>% select(SampleID, KeptReads)
```

```
library(data.table)

dat_tot <- data.table(as(sample_data(z), "data.frame"), TotalReads = sample_sums(z), keep.row
names = TRUE)


dat_fil <- data.table(as(sample_data(Euka_tranformed), "data.frame"), KeptReads = sample_sums
(Euka_tranformed), keep.rownames = TRUE)

dat_tot <- dat_tot %>% select(SampleID, TotalReads)
dat_fil <- dat_fil %>% select(SampleID, KeptReads)

dat <- dat_tot %>% left_join(dat_fil) %>% mutate(PercKept = KeptReads/TotalReads*100)
```

```
## Joining with `by = join_by(SampleID)`
```

Save and load

```
#save.image("2024-01-12_18S_CMargot.RData")
load("2024-01-12_18S_CMargot.RData")
```

Compatibility Check

```
sink("SessionInfo.txt")
  sessionInfo()
```

```
## R version 4.3.1 (2023-06-16 ucrt)
## Platform: x86_64-w64-mingw32/x64 (64-bit)
## Running under: Windows 10 x64 (build 19045)
##
## Matrix products: default
##
##
## locale:
## [1] LC_COLLATE=German_Switzerland.utf8  LC_CTYPE=German_Switzerland.utf8
## [3] LC_MONETARY=German_Switzerland.utf8 LC_NUMERIC=C
## [5] LC_TIME=German_Switzerland.utf8
##
## time zone: Europe/Zurich
## tzcode source: internal
##
## attached base packages:
## [1] stats     graphics  grDevices utils     datasets  methods   base
##
## other attached packages:
##  [1] data.table_1.14.8 ranacapa_0.1.0    decontam_1.20.0   ggthemes_4.2.4
##  [5] microbiome_1.22.0 phyloseq_1.44.0   vegan_2.6-4       lattice_0.21-8
##  [9] permute_0.9-7     ggpubr_0.6.0      ggplot2_3.4.2     tidyr_1.3.0
## [13] dplyr_1.1.2       descr_1.1.7
##
## loaded via a namespace (and not attached):
##  [1] bitops_1.0-7            rlang_1.1.1             magrittr_2.0.3
##  [4] ade4_1.7-22             compiler_4.3.1          mgcv_1.9-0
##  [7] systemfonts_1.0.4       vctrs_0.6.3             reshape2_1.4.4
## [10] stringr_1.5.0           pkgconfig_2.0.3         crayon_1.5.2
## [13] fastmap_1.1.1           backports_1.4.1         XVector_0.40.0
## [16] labeling_0.4.2          utf8_1.2.3              rmarkdown_2.23
## [19] ragg_1.2.5              purrr_1.0.1             xfun_0.39
## [22] zlibbioc_1.46.0         cachem_1.0.8            GenomeInfoDb_1.36.1
## [25] jsonlite_1.8.7          biomformat_1.28.0       highr_0.10
## [28] rhdf5filters_1.12.1     Rhdf5lib_1.22.0         broom_1.0.5
## [31] parallel_4.3.1          cluster_2.1.4           R6_2.5.1
## [34] bslib_0.5.1             stringi_1.7.12          car_3.1-2
## [37] jquerylib_0.1.4         Rcpp_1.0.10             iterators_1.0.14
## [40] knitr_1.43              IRanges_2.34.1          Matrix_1.6-0
## [43] splines_4.3.1           igraph_1.5.1            tidyselect_1.2.0
## [46] rstudioapi_0.15.0       abind_1.4-5             yaml_2.3.7
## [49] codetools_0.2-19        tibble_3.2.1            plyr_1.8.8
## [52] Biobase_2.60.0          withr_2.5.0             evaluate_0.21
## [55] Rtsne_0.16              survival_3.5-5          Biostrings_2.68.1
## [58] pillar_1.9.0            carData_3.0-5           foreach_1.5.2
## [61] stats4_4.3.1            generics_0.1.3          RCurl_1.98-1.12
## [64] S4Vectors_0.38.1        munsell_0.5.0           scales_1.2.1
## [67] xtable_1.8-4            glue_1.6.2              tools_4.3.1
## [70] ggsignif_0.6.4          rhdf5_2.44.0            grid_4.3.1
## [73] ape_5.7-1               colorspace_2.1-0        nlme_3.1-163
## [76] GenomeInfoDbData_1.2.10 cli_3.6.1               textshaping_0.3.6
## [79] fansi_1.0.4             gtable_0.3.3            rstatix_0.7.2
## [82] sass_0.4.7              digest_0.6.31           BiocGenerics_0.46.0
```

```
## [85] farver_2.1.1           htmltools_0.5.5        multtest_2.56.0
## [88] lifecycle_1.0.3        MASS_7.3-60
```

```
sink()
```
